# Enantiopure Turbo Chirality Targets in Tri-Propeller Blades: Design, Asymmetric Synthesis, and Computational Analysis

**DOI:** 10.3390/molecules30030603

**Published:** 2025-01-29

**Authors:** Yu Wang, Ting Xu, Ankit Pandey, Shengzhou Jin, Jasmine X. Yan, Qingkai Yuan, Sai Zhang, Jia-Yin Wang, Ruibin Liang, Guigen Li

**Affiliations:** 1 School of Chemistry and Chemical Engineering, Nanjing University, Nanjing 210093, China; junfu23wang@163.com (Y.W.);; 2 Department of Chemistry and Biochemistry, Texas Tech University, Lubbock, TX 79409-1061, USA; 3 School of Pharmacy, Continuous Flow Engineering Laboratory of National Petroleum and Chemical Industry, Changzhou University, Changzhou 213164, China; zhangsai@cczu.edu.cn (S.Z.);

**Keywords:** asymmetric synthesis, enantiopure turbo chirality, propeller chirality, phosphine oxide, propeller blades

## Abstract

Enantiopure turbo chirality in small organic molecules, without other chiral elements, is a fascinating topic that has garnered significant interest within the chemical and materials science community. However, further research into and application of this concept have been severely limited by the lack of effective asymmetric tools. To date, only a few enantiomers of turbo chiral targets have been isolated, and these were obtained through physical separation using chiral HPLC, typically on milligram scales. In this work, we report the first asymmetric approach to enantiopure turbo chirality in the absence of other chiral elements such as central and axial chirality. This is demonstrated by assembling aromatic phosphine oxides, where three propeller-like groups are anchored to a P(O) center via three axes. Asymmetric induction was successfully carried out using a chiral sulfonimine auxiliary, with absolute configurations and conformations unambiguously determined by X-ray diffraction analysis. The resulting turbo frameworks exhibit three propellers arranged in either a clockwise (*P*,*P*,*P*) or counterclockwise (*M*,*M*,*M*) configuration. In these arrangements, the bulkier sides of the aromatic rings are oriented toward the oxygen atom of the P=O bond rather than in the opposite direction. Additionally, the orientational configuration is controlled by the sulfonimine auxiliary as well, showing that one of the Naph rings is pushed away from the auxiliary group (-CH_2_-NHSO_2_-*t*Bu) of the phenyl ring. Computational studies were conducted on relative energies for the rotational barriers of a turbo target along the P=O axis and the transition pathway between two enantiomers, meeting our expectations. This work is expected to have a significant impact on the fields of chemistry, biomedicine, and materials science in the future.

## 1. Introduction

The significance of molecular chirality has been widely recognized since its discovery by Pasteur over a century ago [1]. Interest in chirality research intensified after the first optical amino acid, tyrosine, was identified [2,3], followed by the characterization of right- and left-handed α-helices in proteins [4,5] and the double helix structure of DNA [5,6]. These early breakthroughs revolutionized the fields of biology, medicine, chemistry, and materials science [7,8,9,10,11,12,13,14]. In chemical sciences, substantial progress has been made in controlling molecular chirality [15,16,17,18,19,20,21,22,23,24,25,26,27,28,29,30,31,32], with several Nobel Prizes awarded for advancements in this area [33,34].

**Figure 1:**
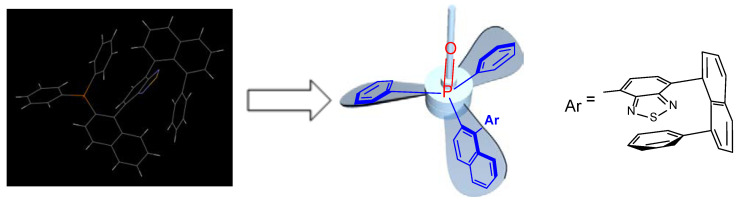
Turbo chirality in multilayer frameworks.

Molecular chirality in chemistry is categorized in various ways, including central [35,36,37,38,39,40,41,42,43,44,45,46,47,48,49,50,51,52,53,54,55,56,57,58,59,60], axial [61,62,63,64,65,66,67,68,69,70,71,72,73,74,75,76,77,78,79,80,81], spiral [15,82,83], sandwich (both metallic [84,85] and organo [86,87,88,89,90,91]), turbo or propeller chirality [92], and orientational chirality [93,94,95,96] in small molecules. Other classifications include multilayer chirality (rigid helical [11,97] and flexible folding [98,99]), as well as topological and inherent chirality in macromolecules and polymers [100,101]. Recently, our lab has focused on establishing new chirality elements and advancing asymmetric control strategies. For instance, we have uniquely characterized orientational chirality, defined by C(sp^2^)-C(sp^3^) or C(sp)-C(sp^3^) axes anchored by chiral centers and remote blockers [93,94,95,96]. The chiral model for this element shows only three major energy barriers, in contrast to the six barriers typically observed in classic Felkin–Ahn or Cram models. Additionally, we developed a multilayer folding chirality based on a P=O scaffold, which consists of three parallel aromatic rings (top, middle, and bottom layers). This folding chiral framework can be controlled via asymmetric catalysis to facilitate single C-C bond formation, using newly designed chiral phosphine amide catalysts [86]. An X-ray diffraction analysis of the P=O-based multilayer framework confirmed the presence of a pro-phosphorus chiral center and a turbo or propeller chiral pattern (Figure 1). The concurrent aromatic–aromatic interactions led to the differentiation of the two phenyl groups on the P=O unit and induced the turbo chiral arrangement across the three aromatic rings.

**Figure 2:**
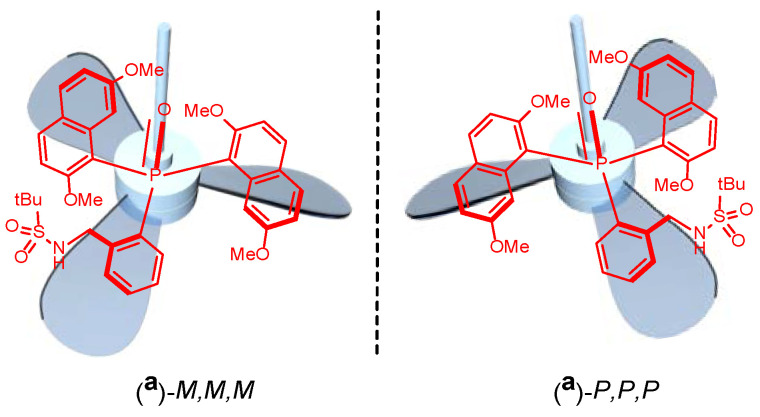
Enantiopure turbo chiral targets.

Although the new chiral elements described above exhibit either center or planar chirality, they inspired us to pursue enantiopure turbo chirality devoid of other chiral elements. A review of the literature revealed that only a few enantiomers of turbo chiral compounds have been isolated, primarily through physical separation using chiral HPLC, typically on milligram scales [102,103]. The further exploration and application of this intriguing form of chirality have been significantly constrained by the lack of effective asymmetric synthetic methods. To the best of our knowledge, no reports have yet described the asymmetric synthesis of turbo chiral targets in the absence of other chiral elements [104,105,106,107,108,109]. To address this challenge, we chose phosphine oxides as our starting point, based on our experience with their synthesis and their ability to form high-quality crystals suitable for X-ray diffraction analysis. In this report, we present our preliminary findings on the first synthesis of enantiopure turbo chiral frameworks, as illustrated in Figure 2.

## 2. Results and Discussion

The design and synthesis in this work consist of assembling the turbo target of *N*-(2-(bis(2,7-dimethoxynaphthalen-1-yl)phosphoryl)benzyl)-2-methylpropane-2-sulfonamide. Initially, we focused on the 1-substituted-(2,7-dimethoxynaphthalene) scaffold, widely used for axial chirality control [110,111,112,113] due to the steric interactions between the hydrogen and methoxy groups at positions 1 and 2 of the naphthalene ring. Introducing two such groups would lead to bulkier stereochemical surroundings which benefit the stability of atropoisomeric chiral turbo targets. The turbo design with the *N*-(2-benzyl)-2-methylpropane-2-sulfonamide group was based on three key factors: the non-symmetrical structure, the ease of attaching chiral auxiliaries, and the potential for forming P-C(sp^2^) bonds under well-established catalytic conditions. In regard to auxiliary selection, the chiral sulfinyl auxiliary was chosen since it has been successfully utilized for controlling our orientational chirality [114,115], and it is readily oxidized to a -SO_2_tBu functionality to eliminate chiral centers in the final step. The synthesis involved two main building blocks: (*R*)- or (*S*)-*N*-(2-bromobenzyl)-2-methylpropane-2-sulfinamide (1) and bis(2,7-dimethoxynaphthalen-1-yl)phosphine oxide (2).

**Scheme 1:**
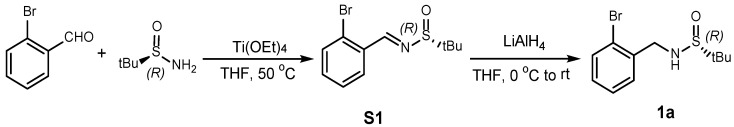
Synthesis of the building block **1a**.

**Scheme 2:**
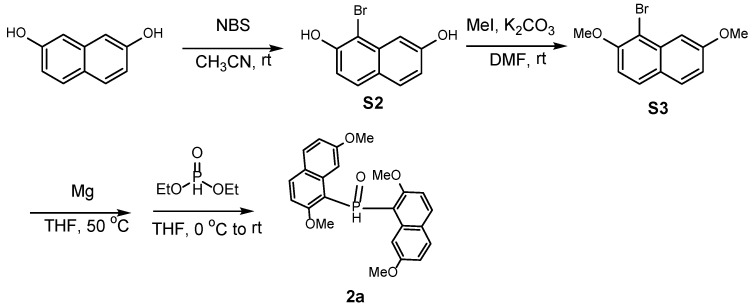
Synthesis of the building block **2a**.

**Scheme 3:**
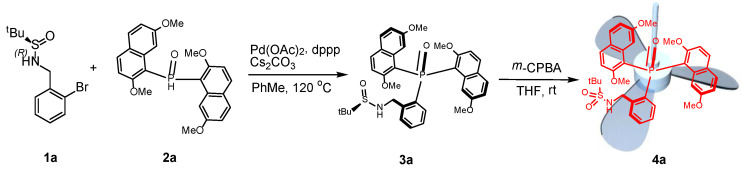
Assembly of the final turbo target.

The synthesis of building block **1a** started with the treatment of a commercial starting material, 2-bromobenzaldehyde, with *tert*-butanesulfinamide in the presence of titanium tetraethoxide to give (*R*,*E*)-*N*-(2-bromobenzylidene)-2-methylpropane-2-sulfinamide (**S1**, 92%) [69,70]. This chiral imine precursor was reduced to (*R*)- or (*S*)-*N*-(2-bromobenzyl)-2-methylpropane-2-sulfinamide by using LiAlH_4_ (0.5 equiv) in THF at 0 °C to r.t. for 10 min in a chemical yield of 88%. In this step, the crude chiral imine precursor was directly subjected to the reduction reaction without purification. The synthesis of the second building block started with the bromination reaction of 2,7-dihydroxynaphthalene with NBS in a yield of 93%. The resulting 1-bromonaphthalene-2,7-diol (**S2**) was subjected to demethylation with methyl iodide (for others, alkyl bromides were employed) in the presence of potassium carbonate to give 1-bromo-2,7-dimethoxynaphthalene (**S3**) in a 94% yield. 1-Bromo-2,7-dimethoxynaphthalene was then treated with magnesium turnings to afford the corresponding Grignard reagent, which was then reacted with diethyl phosphite to obtain bis(2,7-dimethoxynaphthalen-1-yl)phosphine oxide **2a**. Under a catalytic system involving Pd(OAc)_2_ (10 mol%), dppp (20 mol%), and Cs_2_CO_3_ (2.0 equiv) in toluene solution protected by argon gas, the reaction was completed at 120 °C in 16 h to give (*R*)-*N*-(2-(bis(2,7-dimethoxynaphthalen-1-yl)phosphoryl)benzyl)-2-methylpropane-2-sulfinamide (**3a**) in a complex mixture which is extremely difficult to purify. This crude product was directly subjected to oxidation with *m*-CPBA in THF to afford the final chiral turbo product, *N*-(2-(bis(2,7-dimethoxynaphthalen-1-yl)phosphoryl)benzyl)-2-methylpropane-2-sulfonamide (**4a**), in a chemical yield of 80% for these two steps (an overall yield of 44% from **2a**) (Scheme 1, Scheme 2 and Scheme 3).

**Figure 3:**
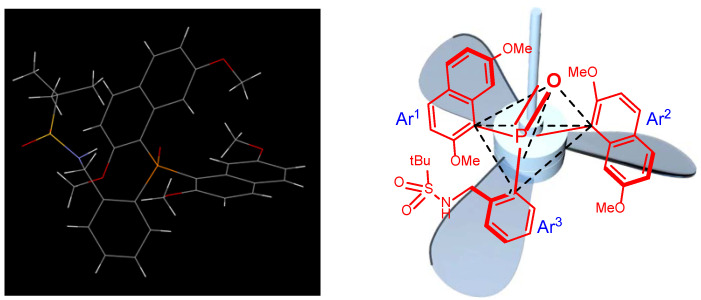
Turbo chirality represented by phosphine oxide target (**4a**).

The absolute configuration of the resulting turbo chirality has been unambiguously determined by X-ray diffraction analysis (Figure 3). The (*S*)-2-methylpropane-2-sulfonimine auxiliary leads to the asymmetric formation of turbo chiral target (**5a**) in a *P*,*P*,*P* configuration, as clearly indicated by the arrangement of three aromatic rings. Interestingly, a chiral nitrogen center is also observed in this linearly connected amino functionality which is anchored by the electron-withdrawing sulfonyl group (tBuSO_2_-). The lone-pair electrons on nitrogen are directed between two S=O bonds equally. Surrounding the tetrahedron unit centered by phosphorus (black broken line in Figure 3), the more stereochemically bulkier sides of the three aromatic rings are directed closer to the oxygen atom of P=O instead of in the opposite direction. The three bond angles of the Csp^2^(Ar)-P and P=O bonds are measured as 108.62° [Csp^2^(Ar^1^)], 113.34° [Csp^2^(Ar^2^)], and 107.06° [Ar^3^]. The differences in these bond angles lead to notable differences in the bond distances of the three Csp^2^(Ar)-P bonds, with values of 1.829 Å [Csp^2^(Ar^1^)], 1.825 Å [Csp^2^(Ar^2^)], and 1.822 Å [Csp^2^(Ar^3^)]. Accordingly, the distances between the oxygen on the P=O bond and these three sp^2^ carbons are determined to be 2.698 Å [Csp^2^(Ar^1^)], 2.770 Å [Csp^2^(Ar^2^)], and 2.666 Å [Csp^2^(Ar^3^)]. More importantly, the present turbo chiral framework clearly shows three dihedral angles of P=O with aromatic rings, with values of 48.70° [Csp^2^(Ar^1^)], 7.25° [Csp^2^(Ar^2^)], and 59.33° [Csp^2^(Ar^3^)].

**Figure 4:**
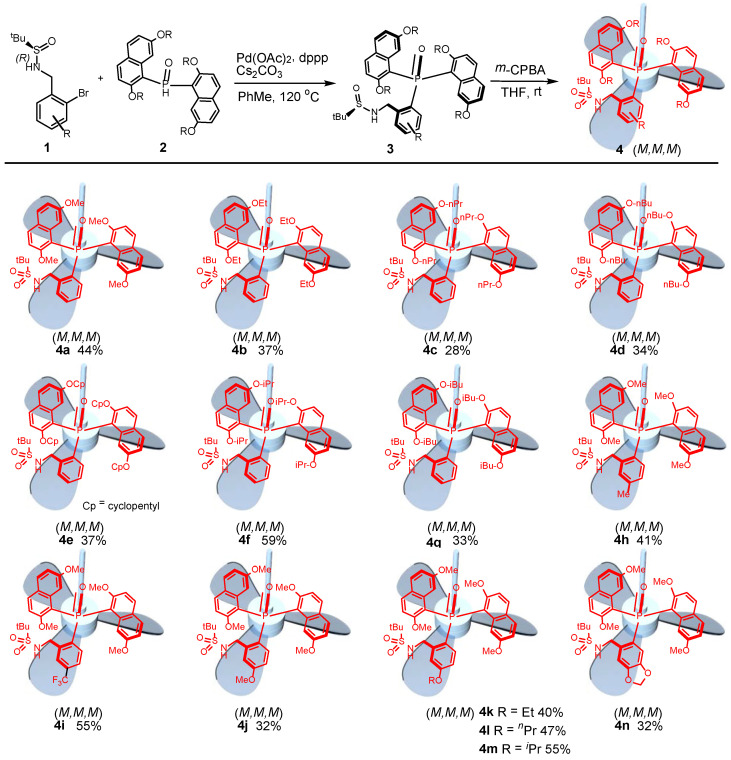
Synthetic results of P(O)–turbo chiral targets **I**.

**Figure 5:**
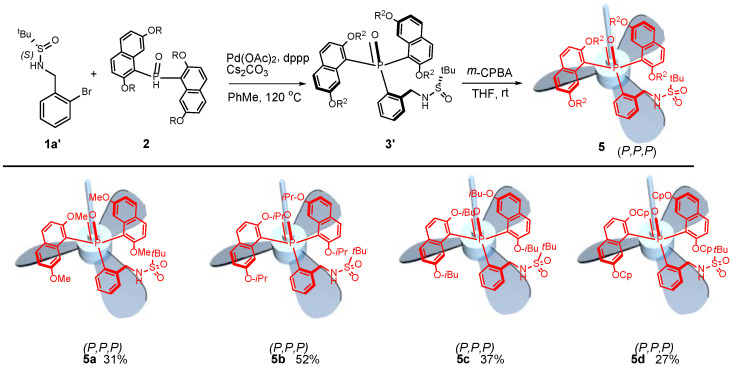
Synthetic results of P(O)–turbo chiral targets **II**.

With this fully characterized turbo framework (**4a**) on hand, we then expanded the design and asymmetric synthesis of a series of other turbo targets with both *P*,*P*,*P* and *M*,*M*,*M* configurations (Figure 4 and Figure 5). For *P*,*P*,*P* configuration target synthesis, alkoxy groups on the 2,7-positions of the naphthalene ring were considered to enhance the steric effects compared to the smallest MeO-group. This would help to increase the atropoisomeric barriers of the three P-C(sp^2^) bonds, benefiting the stability of the turbo chiral compounds. Obviously, at first, primary alkyl groups, such as Et, *n*-Pr, and *n*-Bu, should be employed to replace the smallest Me counterpart. As shown in Figure 4, the corresponding products were obtained in yields of 37%, 28%, and 34% for cases **4b**, **4c**, and **4d**, respectively. Next, secondary and tertiary alkyl groups, cyclopentyl (Cp), *i*-Pr, and *i*-Bu, were employed to further increase the steric effect for the higher stability of the chiral turbo targets. Chemical yields of 37%, 59%, and 33% were obtained for cases **4e**, **4f**, and **4g**, respectively. For nearly all of these cases except for case **4f**, chemical yields are lower than that of MeO case (44%), which may be caused by higher steric effects. The substrate scope was also examined for (*R*)-*N*-(2-bromobenzyl)-2-methylpropane-2-sulfinamide by introducing various groups in the phenyl ring. The methyl-substituted case (**4h**) gave almost an identical yield (41%) to the initial case. The electron-withdrawing group of CF_3_ (**4i**) afforded a higher yield of 55% than that of the initial case. Five strong electron-donating cases (**4j**, **4k**–**4m**, and **4n**) all work well for the present asymmetric synthesis. Cases **4j** and **4n** gave the same chemical yield of 32%, whereas the yields of cases **4k**–**4m** were arranged from 40% to 55%.

For *M*,*M*,*M* configuration target synthesis, the opposite chiral auxiliary of (*S*)-*N*-(2-bromobenzyl)-2-methylpropane-2-sulfinamide was utilized by varying alkoxy groups on the 2,7-positions of the naphthalene ring. As shown in Figure 5, the four turbo targets cover three types of alkyl groups, including primary (Me) and secondary (*i-*Pr, *i*-Bu, and Cp) groups. Similar ranges of chemical yields to those of the *P*,*P*,*P* configuration cases were obtained (31%, 52%, 37%, and 27% for case **5a**, **5b**, **5c**, and **5d**, respectively). Interestingly, compared to the initial case of MeO, nearly all cases afforded lower chemical yields due to the steric effect, as anticipated. However, three *i*-Pr cases all gave higher yields (52–59%) which might be caused by the aggregation-induced factor [18,19] due to the existence of a *i*-Pr group, benefiting products’ packing or aggregation. For all of the above-mentioned synthesis, precursors **3’** were extremely difficult to isolate and purify due to the formation of complex products. They were directly subjected to the oxidation step to afford the final products, which can be more easily purified for the generation of good-quality crystals for X-ray structural analysis.

### Computational Study

**Figure 6:**
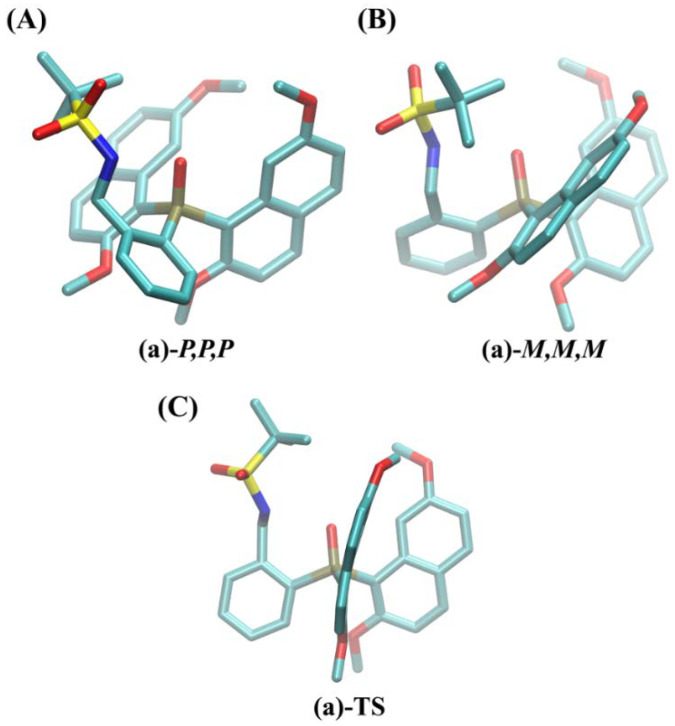
Structures of the stationary points along the pathway (minima and transition states) connecting the two enantiomers. (**A**) The (a)-P,P,P enantiomer. (**B**) The (a)-M,M,M enantiomer. (**C**) The transition state. The C, N, O, S, and P atoms are colored in cyan, blue, red, yellow, and brown, respectively.

We conducted density functional theory (DFT) calculations for the turbo chirality based on the computation methods discussed below to determine the minimum energy pathway (MEP) connecting (a)-*P*,*P*,*P* (Enantiomer 1) and (a)-*M*,*M*,*M* (Enantiomer 2). Enantiomer 1 was obtained from the crystal structure. Enantiomer 2 was created as the mirror image of Enantiomer 1 across the x-z plane, generating its mirror image. This reflected structure was then superposed on Enantiomer 1 using the central S, N, and C atoms (Figure 6) as reference atoms, which led to the M,M,M turbo chirality for the three substituent groups of the phosphine oxide. These two initial structures were optimized using DFT with the B3LYP functional [116,117,118,119], incorporating D3 dispersion corrections [120] and the def2-SVP basis set [121] (B3LYP-D3/def2-SVP).

The geodesic interpolation method was employed to generate an initial guess of the MEP connecting the two optimized enantiomers. The initial path contained 20 images and was subsequently optimized using the nudged elastic band (NEB) method while the two endpoints were frozen. The climbing image from the NEB search served as an initial guess for the more refined optimization of the transition state (TS) structures using the dimer method [122]. Both NEB and dimer method calculations were performed using the B3LYP-D3/def2-SVP approach. The Hessians at the optimized minima and TSs were evaluated using the same quantum mechanical method, and normal mode analysis confirmed the identities of the TSs and minima: each TS had a single imaginary-frequency mode, while each minimum had none.

The energies of the previously optimized stationary points (two minima and one TS) were recalculated at the B3LYP-D3/def2-TZVP level of theory. Zero-point energy (ZPE) corrections, derived from normal mode analysis, were added to the B3LYP-D3/def2-TZVP energies. All geometry optimizations, reaction pathway searches, and ZPE corrections were conducted using the TURBOMOLE [123] software package, which was interfaced with the DL-FIND code [124] within the Chemshell software package [125].

Transition state theory (Equation (1)) was applied to estimate the reaction rate for the interconversion between the two enantiomers:

(1) $$k_{TST}=\frac{k_{B}T}{h}\cdot e^{-\beta ∆E},$$

where ∆E is the free energy barrier, β equals $1/(k_{B}T)$, $k_{B}$ is the Boltzmann’s constant, *T* is the temperature (298.0 K), and h is the Planck constant.

Through geometry optimization and a reaction path search at the DFT level of theory, we identified the structures of the minima corresponding to the two enantiomers ((a)-*M*,*M*,*M*) and ((a)-*P*,*P*,*P*) and the transition state (TS) along their interconversion pathway (Figure 6). The calculated energy barrier for the transition between the two enantiomers is 26.38 kcal/mol. Obtained using transition state theory, the estimated rate for the transition is $2.78\times 10^{-7}\;s^{-1}$ at room temperature (298 K). The inverse of this rate, which represents the lifetime of the reactant, is approximately $3.59\times 10^{6}\;s$ (or about 41.6 days). These results suggest that the high energy barrier can effectively kinetically stabilize each enantiomer. The elevated energy of the TS is attributed to the steric repulsion between the bulky *t*-butyl group and the two methoxy groups (Figure 6C), one from each of the two naphthalene rings.

## 3. Materials and Methods

### 3.1. General Methods

Unless otherwise stated, all reactions used magnetically stirred mixtures and were conducted in oven-dried glassware in anhydrous solvents under Ar, applying standard Schlenk techniques. Solvents and liquid reagents, as well as solutions of solid or liquid reagents, were added via syringes and stainless steel or polyethylene cannulas through rubber septa or through a weak Ar counter-flow. Solvents were removed under reduced pressure at 40–65 °C using a rotavapor. All given yields are the isolated yields of chromatographically and NMR spectroscopically assessed materials. All commercially available chemicals were used as received without further purification.

^1^H and ^13^C NMR spectra were recorded in CDCl_3_ on 400 MHz instruments with TMS as an internal standard. For the referencing of the ^1^H NMR spectra, the residual solvent signal (δ = 7.26 ppm for CDCl_3_) was used. In the case of the ^13^C NMR spectra, the signal of the solvent (δ = 77.0 ppm for CDCl_3_) was used. Chemical shifts (δ) were reported in ppm with respect to TMS. Data are represented as follows: chemical shift, multiplicity (s = singlet; d = doublet; t = triplet; m = multiplet), coupling constant (*J*, Hz), and integration. Optical rotations were measured with a Rudolph Research Analytical APIV/2W Polarimeter (Hackettstown, NJ, USA) at the indicated temperature with a sodium lamp. Measurements were performed in a 2 mL vessel with a concentration unit of g/100 mL in the corresponding solvents.

### 3.2. Chemical Synthesis

#### 3.2.1. General Procedure A for the Synthesis of Sulfonamides **1a**, **1a’**, **1b**, **1c**, and **1h**

Substrate **1** was prepared according to the reported procedures and the related literature [126,127]. Titanium tetraethoxide (2.22 g, 2.0 equiv, 10 mmol) was slowly added to a solution of (*R*)-*tert*-butanesulfinamide (0.61 g, 1.0 equiv, 5 mmol) and 2-bromobenzaldehyde (1.0 equiv, 5 mmol) in dry THF (20 mL) at 50 °C. After finishing (noticed by using TLC), 1 mL of saturated aq was added to the reaction mixture. The NaHCO_3_ solution and precipitated product were collected by filtration and washed thoroughly with ethyl acetate. The filtrate was concentrated under reduced pressure and used without further purification in the next step.

The resulting crude aldimine was then dissolved in THF (0.20 M). After the solution was cooled to 0 ºC with an ice bath, a solution of LiAlH_4_ (2.5 mL, 0.5 equiv, 2.5 mmol) in THF (1.0 M) was added dropwise, and the reaction mixture was allowed to warm to room temperature. After being stirred for 10 min and monitored by TLC, the reaction was quenched with a saturated solution of NH_4_Cl (20 mL) and extracted with EtOAc (2 × 20 mL). The combined organic layers were washed with brine, dried over MgSO_4_, and filtered. The residue was purified after concentration on silica gel (*n*-hexane/EA = 3:1) to obtain the pure sulfonamide **1**.

*(R)-N-(2-Bromobenzyl)-2-methylpropane-2-sulfinamide* (**1a**). General procedure A was employed, and the product was purified by silica gel (200–300 mesh) column chromatography using petroleum ether/ethyl acetate (3:1 *v*/*v*) as eluent to obtain **1a** as a white solid. ^1^H NMR (400 MHz, CDCl_3_): *δ* 7.56 (dd, *J* = 7.9, 1.3 Hz, 1H), 7.42 (dd, *J* = 7.6, 1.7 Hz, 1H), 7.30 (td, *J* = 7.5, 1.3 Hz, 1H), 7.16 (td, *J* = 7.6, 1.8 Hz, 1H), 4.50–4.29 (m, 2H), 3.64 (t, *J* = 6.6 Hz, 1H), 1.23 (s, 9H) ppm; ^13^C NMR (101 MHz, CDCl_3_): *δ* 137.9, 133.0, 130.2, 129.3, 127.6, 123.9, 56.1, 49.7, 22.6 ppm. HRMS (ESI): *m*/*z* calcd. for C_11_H_16_BrNOS [M + Na]^+^ 312.0029, found 312.0040.

*(S)-N-(2-Bromobenzyl)-2-methylpropane-2-sulfinamide* (**1a’**). General procedure A was employed using (*S*)-*tert*-butanesulfinamide, which was purified by silica gel (200–300 mesh) column chromatography using petroleum ether/ethyl acetate (3:1 *v*/*v*) as eluent to obtain **1a’** as a white solid. ^1^H NMR (400 MHz, CDCl_3_): *δ* 7.56 (dd, *J* = 7.9, 1.2 Hz, 1H), 7.42 (dd, *J* = 7.6, 1.7 Hz, 1H), 7.30 (td, *J* = 7.5, 1.3 Hz, 1H), 7.16 (td, *J* = 7.7, 1.7 Hz, 1H), 4.49–4.30 (m, 2H), 3.64 (t, *J* = 6.4 Hz, 1H), 1.23 (s, 9H) ppm; ^13^C NMR (101 MHz, CDCl_3_): *δ* 137.9, 133.0, 130.2, 129.3, 127.7, 123.9, 56.1, 49.8, 22.6 ppm. HRMS (ESI): *m*/*z* calcd. for C_11_H_16_BrNOS [M + H]^+^ 290.0209, found 290.0211.

*(R)-N-(2-Bromo-5-methylbenzyl)-2-methylpropane-2-sulfinamide* (**1b**). General procedure A was employed, and the product was purified by silica gel (200–300 mesh) column chromatography using petroleum ether/ethyl acetate (3:1 *v*/*v*) as eluent to obtain **1b** as a white solid. ^1^H NMR (400 MHz, CDCl_3_): *δ* 7.42 (d, *J* = 8.1 Hz, 1H), 7.21 (d, *J* = 2.3 Hz, 1H), 7.00–6.92 (m, 1H), 4.43–4.25 (m, 2H), 3.63 (t, *J* = 6.4 Hz, 1H), 2.31 (s, 3H), 1.23 (s, 9H) ppm; ^13^C NMR (101 MHz, CDCl_3_): *δ* 137.6, 137.4, 132.7, 131.1, 130.0, 120.5, 56.0, 49.7, 22.6, 20.8 ppm. HRMS (ESI): *m*/*z* calcd. for C_12_H_18_BrNOS [M + Na]^+^ 326.0185, found 326.0184.

*(R)-N-(2-Bromo-5-(trifluoromethyl)benzyl)-2-methylpropane-2-sulfinamide* (**1c**). General procedure A was employed, and the product was purified by silica gel (200–300 mesh) column chromatography using petroleum ether/ethyl acetate (3:1 *v*/*v*) as eluent to obtain **1c** as a white solid. ^1^H NMR (400 MHz, CDCl_3_): *δ* 7.72–7.68 (m, 2H), 7.42 (dd, *J* = 8.5, 2.1 Hz, 1H), 4.53–4.36 (m, 2H), 3.70 (t, *J* = 6.6 Hz, 1H), 1.26 (s, 9H) ppm; ^13^C NMR (101 MHz, CDCl_3_): *δ* 139.1, 133.5, 130.3, 130.0, 127.5, 126.6 (q, *J* = 4.0 Hz), 125.8 (q, *J* = 3.7 Hz), 125.0, 122.3, 56.3, 49.1, 22.5 ppm; ^19^F NMR (376 MHz, CDCl_3_) *δ* −62.8 ppm. HRMS (ESI): *m*/*z* calcd. for C_12_H_15_BrF_3_NOS [M + Na]^+^ 379.9903, found 379.9898.

*(R)-N-(2-Bromo-5-methoxybenzyl)-2-methylpropane-2-sulfinamide* (**1d**). General procedure A was employed, and the product was purified by silica gel (200–300 mesh) column chromatography using petroleum ether/ethyl acetate (3:1 *v*/*v*) as eluent to obtain **1d** as a white solid. ^1^H NMR (400 MHz, CDCl_3_): *δ* 7.43 (d, *J* = 8.7 Hz, 1H), 7.00 (d, *J* = 3.0 Hz, 1H), 6.72 (dd, *J* = 8.7, 3.0 Hz, 1H), 4.44–4.26 (m, 2H), 3.79 (s, 3H), 3.68 (t, *J* = 6.6 Hz, 1H), 1.24 (s, 9H). ppm; ^13^C NMR (101 MHz, CDCl_3_): *δ* 159.1, 138.8, 133.5, 115.8, 114.8, 113.9, 56.1, 55.5, 49.8, 22.6 ppm. HRMS (ESI): *m*/*z* calcd. for C_12_H_18_BrNO_2_S [M + Na]^+^ 342.0134, found 324.0135.

*(R)-N-((6-Bromobenzo[d][1,3]dioxol-5-yl)methyl)-2-methylpropane-2-sulfinamide* (**1h**). General procedure A was employed, and the product was purified by silica gel (200–300 mesh) column chromatography using petroleum ether/ethyl acetate (3:1 *v*/*v*) as eluent to obtain **1h** as a white solid. ^1^H NMR (400 MHz, CDCl_3_): *δ* 7.00 (s, 1H), 6.90 (s, 1H), 5.97 (q, *J* = 1.4 Hz, 2H), 4.39–4.19 (m, 2H), 3.63–3.56 (m, 1H), 1.23 (s, 9H) ppm; ^13^C NMR (101 MHz, CDCl_3_): *δ* 147.9, 147.5, 131.0, 114.3, 112.9, 110.0, 101.8, 56.0, 49.6, 22.6 ppm. HRMS (ESI): *m*/*z* calcd. for C_12_H_16_BrNO_3_S [M + H]^+^ 334.0108, found 334.0104.

#### 3.2.2. General Procedure B for the Synthesis of Sulfonamides **1e**–**1g**

According to the reported procedures and the related literature [128], to a solution of 2-bromo-5-hydroxybenzaldehyde (2.01 g, 10 mmol, 1.0 equiv) in DMF (30 mL) were added alkyl bromide (15 mmol, 1.5 equiv) and K_2_CO_3_ (2.77 g, 20 mmol, 2.0 equiv) at room temperature. The reaction mixture was stirred at 50 °C for 4 h. The solution was diluted with water (100 mL) and extracted with EtOAc (3 × 30 mL). The combined organic layer was washed with water (3 × 20 mL) and dried over MgSO_4_. After filtration, the solvent was removed and the crude product was used without further purification in the next step.

Titanium tetraethoxide (2.22 g, 2.0 equiv, 10 mmol) was slowly added to a solution of *tert*-butanesulfinamide (0.61 g, 1.0 equiv, 5 mmol) and 2-bromobenzaldehyde (1.0 equiv, 5 mmol) in dry THF (20 mL) at 50 °C. After finishing (noticed by using TLC), 1 mL of saturated aq was added to the reaction mixture. The NaHCO_3_ solution and precipitated product were collected by filtration and washed thoroughly with EtOAc. The filtrate was concentrated under reduced pressure and used without further purification in the next step.

The resulting crude aldimine was then dissolved in THF (0.20 M). After the solution was cooled to 0 ºC with an ice bath, a solution of LiAlH_4_ (2.5 mL, 0.5 equiv, 2.5 mmol) in THF (1.0 M) was added dropwise, and the reaction mixture was allowed to warm to room temperature. After being stirred for 10 min and monitored by TLC, the reaction was quenched with a saturated solution of NH_4_Cl (20 mL) and extracted with EtOAc (2 × 20 mL). The combined organic layers were washed with brine, dried over MgSO_4_, and filtered. The residue was purified after concentration on silica gel (*n*-hexane/EA = 3:1) to obtain pure sulfonamide **1**.

*(R)-N-(2-Bromo-5-ethoxybenzyl)-2-methylpropane-2-sulfinamide* (**1e**). General procedure B was employed, and the product was purified by silica gel (200–300 mesh) column chromatography using petroleum ether/ethyl acetate (3:1 *v*/*v*) as eluent to obtain **1e** as a white solid. ^1^H NMR (400 MHz, CDCl_3_): *δ* 7.41 (d, *J* = 8.8 Hz, 1H), 6.98 (d, *J* = 3.0 Hz, 1H), 6.70 (dd, *J* = 8.7, 3.0 Hz, 1H), 4.42–4.24 (m, 2H), 4.00 (q, *J* = 7.0 Hz, 2H), 3.66 (t, *J* = 6.6 Hz, 1H), 1.40 (t, *J* = 7.0 Hz, 3H), 1.24 (s, 9H) ppm; ^13^C NMR (101 MHz, CDCl_3_): *δ* 158.4, 138.7, 133.4, 116.3, 115.4, 113.7, 63.7, 56.1, 49.8, 22.6, 14.6 ppm. HRMS (ESI): *m*/*z* calcd. for C_13_H_20_BrNO_2_S [M + Na]^+^ 356.0291, found 356.0284.

*(R)-N-(2-bromo-5-propoxybenzyl)-2-methylpropane-2-sulfinamide* (**1f**). General procedure B was employed, and the product was purified by silica gel (200–300 mesh) column chromatography using petroleum ether/ethyl acetate (3:1 *v*/*v*) as eluent to obtain **1f** as a white solid. ^1^H NMR (400 MHz, CDCl_3_): *δ* 7.41 (d, *J* = 8.7 Hz, 1H), 6.98 (d, *J* = 3.0 Hz, 1H), 6.70 (dd, *J* = 8.7, 3.0 Hz, 1H), 4.39 (dd, *J* = 14.5, 5.7 Hz, 1H), 4.27 (dd, *J* = 14.5, 7.5 Hz, 1H), 3.89 (t, *J* = 6.5 Hz, 2H), 3.72–3.62 (m, 1H), 1.84–1.75 (m, 2H), 1.24 (s, 9H), 1.03 (t, *J* = 7.4 Hz, 3H) ppm; ^13^C NMR (101 MHz, CDCl_3_): *δ* 158.6, 138.7, 133.4, 116.4, 115.4, 113.6, 69.8, 56.1, 49.8, 22.6, 22.4, 10.4 ppm. HRMS (ESI): *m*/*z* calcd. for C_14_H_22_BrNO_2_S [M + Na]^+^ 370.0447, found 370.0454.

*(R)-N-(2-bromo-5-isopropoxybenzyl)-2-methylpropane-2-sulfinamide* (**1g**). General procedure B was employed, and the product was purified by silica gel (200–300 mesh) column chromatography using petroleum ether/ethyl acetate (3:1 *v*/*v*) as eluent to obtain **1g** as a white solid. ^1^H NMR (400 MHz, CDCl_3_): *δ* 7.40 (d, *J* = 8.7 Hz, 1H), 6.97 (d, *J* = 3.0 Hz, 1H), 6.69 (dd, *J* = 8.7, 3.0 Hz, 1H), 4.51 (hept, *J* = 6.0 Hz, 1H), 4.42–4.23 (m, 2H), 3.72–3.63 (m, 1H), 1.32 (d, *J* = 6.0 Hz, 6H), 1.24 (s, 9H) ppm; ^13^C NMR (101 MHz, CDCl_3_): *δ* 157.4, 138.7, 133.5, 117.6, 116.6, 113.5, 70.3, 56.1, 49.8, 22.6, 21.9, 21.8 ppm. HRMS (ESI): *m*/*z* calcd. for C_14_H_22_BrNO_2_S [M + Na]^+^ 370.0447, found 370.0443.

#### 3.2.3. General Procedure for the Synthesis of Phosphine Oxides **2**

Substrates **2** were prepared according to the reported procedures and the related literature [129,130]. To a solution of 2,7-dihydroxynaphthalene (3.21 g, 20 mmol, 1.0 equiv) in MeCN (50 mL) was added NBS (3.92 g, 22 mmol, 1.1 equiv). After being stirred for 8 h at room temperature, the reaction mixture was diluted with EtOAc, and the organic material was extracted with EtOAc. The combined organic layers were washed with brine and dried over MgSO_4_. After filtration, the filtrate was concentrated under reduced pressure. The residue was purified by column chromatography on silica gel (*n*-hexane/EtOAc = 4/1) to provide 1-bromonaphthalene-2,7-diol.

To a solution of 1-bromonaphthalene-2,7-diol (2.39 g, 10 mmol, 1.0 equiv) in DMF (30 mL) were added alkyl bromide (40 mmol, 4.0 equiv) and K_2_CO_3_ (5.53 g, 40 mmol, 4.0 equiv) at room temperature. The reaction mixture was stirred at 40 °C for 8 h. The solution was diluted with water (100 mL) and extracted with EtOAc (3 × 30 mL). The combined organic layer was washed with water (3 × 20 mL) and dried over MgSO_4_. After filtration, the solvent was removed by evaporation, and the resulting mixture was purified by column chromatography on silica gel (*n*-hexane) to afford the corresponding bromo-aryl ether.

Under an argon atmosphere, a dried flask with a reflux condenser was charged with magnesium turnings (0.79 g, 33 mmol, 3.3 equiv) and THF (5 mL). Then, a solution of the corresponding bromo-aryl ether (30 mol, 3.0 equiv) in 20 mL of THF was added dropwise, and the Grignard reagent formation was started by heating at 60 °C. After the reaction had started, the reaction mixture was maintained for 2 h at 60 °C. Under an argon atmosphere, a second dried flask was charged with NaH (60% in mineral oil, 0.48 g, 12 mmol, 1.2 equiv) and THF. Then, this mixture was cooled in an ice bath, and at 0 °C, diethyl phosphite (1.38 g, 10 mmol, 1.0 equiv) was added dropwise over 15 min. Afterward, the reaction mixture was stirred for 30 min at 0 °C, and then, the freshly prepared Grignard reagent was added dropwise. After addition, the mixture was stirred for 16 h at room temperature and then quenched with a saturated aqueous NH_4_Cl solution. The aqueous layer was extracted with EtOAc (3 × 20 mL), and the combined organic layers were dried over MgSO_4_ and filtered. The residue was purified after concentration on silica gel (*n*-hexane/EA) to obtain pure phosphine oxide **2**.

*Bis(2,7-dimethoxynaphthalen-1-yl)phosphine oxide* (**2a**). The general procedure was employed, and the product was purified by silica gel (200–300 mesh) column chromatography using petroleum ether/ethyl acetate (1:1 *v*/*v*) as eluent to obtain **2a** as a white solid. ^1^H NMR (400 MHz, CDCl_3_): *δ* 9.96 (s, 0.5H), 8.62 (s, 0.5H), 8.44 (d, *J* = 2.4 Hz, 2H), 7.82 (d, *J* = 9.0 Hz, 2H), 7.63 (dd, *J* = 9.0, 1.6 Hz, 2H), 6.99–6.95 (m, 4H), 3.66 (s, 6H), 3.62 (s, 6H) ppm; ^13^C NMR (101 MHz, CDCl_3_): *δ* 160.5 (d, *J* = 2.8 Hz), 159.1, 137.0 (d, *J* = 5.5 Hz), 134.2 (d, *J* = 2.1 Hz), 129.9, 124.5 (d, *J* = 9.4 Hz), 117.1, 113.2, 112.1, 109.8 (d, *J* = 6.6 Hz), 103.2 (d, *J* = 6.8 Hz), 58.2, 56.3, 55.1, 18.3 ppm; ^31^P NMR (162 MHz, CDCl_3_) *δ* 5.1 ppm. HRMS (ESI): *m*/*z* calcd. for C_24_H_23_O_5_P [M + Na]^+^ 445.1176, found 445.1177.

*Bis(2,7-diethoxynaphthalen-1-yl)phosphine oxide* (**2b**). The general procedure was employed, and the product was purified by silica gel (200–300 mesh) column chromatography using petroleum ether/ethyl acetate (1:1 *v*/*v*) as eluent to obtain **2b** as a white solid. ^1^H NMR (400 MHz, CDCl_3_): *δ* 9.95 (s, 0.5H), 8.60 (s, 0.5H), 8.42 (d, *J* = 2.5 Hz, 2H), 7.80 (d, *J* = 9.0 Hz, 2H), 7.62 (dd, *J* = 9.0, 1.6 Hz, 2H), 6.97 (d, *J* = 2.4 Hz, 1H), 6.96–6.93 (m, 2H), 6.92 (d, *J* = 5.1 Hz, 1H), 4.08–4.00 (m, 2H), 3.95–3.83 (m, 4H), 3.56–3.48 (m, 2H), 1.26 (t, *J* = 7.0 Hz, 6H), 1.11 (t, *J* = 7.0 Hz, 6H) ppm; ^13^C NMR (101 MHz, CDCl_3_): *δ* 159.7 (d, *J* = 2.9 Hz), 158.4, 137.2 (d, *J* = 5.6 Hz), 134.0 (d, *J* = 1.9 Hz), 129.8, 124.4 (d, *J* = 9.5 Hz), 117.3, 112.6 (d, *J* = 105.1 Hz), 110.3 (d, *J* = 6.7 Hz), 103.9 (d, *J* = 6.5 Hz), 64.8, 63.4, 14.5, 14.3 ppm; ^31^P NMR (162 MHz, CDCl_3_) *δ* 6.1 ppm. HRMS (ESI): *m*/*z* calcd. for C_28_H_31_O_5_P [M + H]^+^ 479.1892, found 479.1983.

*Bis(2,7-dipropoxynaphthalen-1-yl)phosphine oxide* (**2c**). The general procedure was employed, and the product was purified by silica gel (200–300 mesh) column chromatography using petroleum ether/ethyl acetate (3:1 *v*/*v*) as eluent to obtain **2c** as a white solid. ^1^H NMR (400 MHz, CDCl_3_): *δ* 9.94 (s, 0.5H), 8.59 (s, 0.5H), 8.40 (s, 2H), 7.80 (d, *J* = 9.0 Hz, 2H), 7.62 (d, *J* = 8.9 Hz, 2H), 6.99–6.91 (m, 4H), 3.98–3.92 (m, 2H), 3.87–3.81 (m, 2H), 3.76–3.70 (m, 2H), 3.35–3.30 (m, 2H), 1.70–1.61 (m, 4H), 1.61–1.52 (m, 4H), 0.92 (t, *J* = 7.4 Hz, 6H), 0.86 (t, *J* = 7.4 Hz, 6H) ppm; ^13^C NMR (101 MHz, CDCl_3_): *δ* 159.9 (d, *J* = 2.9 Hz), 158.6, 137.2 (d, *J* = 5.5 Hz), 134.0, 129.8, 124.4 (d, *J* = 9.4 Hz), 117.4, 112.6 (d, *J* = 104.8 Hz), 110.2 (d, *J* = 6.8 Hz), 103.8 (d, *J* = 6.5 Hz), 70.8, 69.3, 22.44, 22.37, 10.5, 10.4 ppm; ^31^P NMR (162 MHz, CDCl_3_) *δ* 5.9 ppm. HRMS (ESI): *m*/*z* calcd. for C_32_H_39_O_5_P [M + Na]^+^ 557.2428, found 557.2415.

*Bis(2,7-dibutoxynaphthalen-1-yl)phosphine oxide* (**2d**). The general procedure was employed, and the product was purified by silica gel (200–300 mesh) column chromatography using petroleum ether/ethyl acetate (3:1 *v*/*v*) as eluent to obtain **2d** as a white solid. ^1^H NMR (400 MHz, CDCl_3_): *δ* 9.90 (s, 0.5H), 8.55 (s, 0.5H), 8.37 (s, 2H), 7.81 (d, *J* = 8.9 Hz, 2H), 7.61 (dd, *J* = 8.9, 1.5 Hz, 2H), 6.95–6.93 (m, 4H), 4.01–3.95 (m, 2H), 3.90–3.84 (m, 2H), 3.79–3.74 (m, 2H), 3.41–3.35 (m, 2H), 1.64–1.57 (m, 4H), 1.52–1.45 (m, 4H), 1.40–1.34 (m, 4H), 1.29–1.23 (m, 4H), 0.90 (t, *J* = 7.4 Hz, 6H), 0.84 (t, *J* = 7.3 Hz, 6H) ppm; ^13^C NMR (101 MHz, CDCl_3_): *δ* 159.9 (d, *J* = 2.9 Hz), 158.7, 137.2 (d, *J* = 5.6 Hz), 134.0 (d, *J* = 2.0 Hz), 129.8, 124.4 (d, *J* = 9.4 Hz), 117.4, 112.5 (d, *J* = 105.1 Hz), 110.1 (d, *J* = 6.6 Hz), 103.7 (d, *J* = 6.4 Hz), 69.0, 67.4, 31.2, 31.1, 19.13, 19.11, 13.79, 13.75 ppm; ^31^P NMR (162 MHz, CDCl_3_) *δ* 6.0 ppm. HRMS (ESI): *m*/*z* calcd. for C_36_H_47_O_5_P [M + H]^+^ 591.3234, found 591.3205.

*Bis(2,7-diisopropoxynaphthalen-1-yl)phosphine oxide* (**2e**). The general procedure was employed, and the product was purified by silica gel (200–300 mesh) column chromatography using petroleum ether/ethyl acetate (3:1 *v*/*v*) as eluent to obtain **2e** as a white solid. ^1^H NMR (400 MHz, CDCl_3_): *δ* 9.88 (s, 0.5H), 8.53 (s, 0.5H), 8.44 (s, 2H), 7.78 (d, *J* = 9.0 Hz, 2H), 7.61 (dd, *J* = 9.0, 1.6 Hz, 2H), 6.96–6.91 (m, 4H), 4.61 (p, *J* = 6.1 Hz, 2H), 4.26 (p, *J* = 6.5 Hz, 2H), 1.26 (d, *J* = 6.0 Hz, 6H), 1.13 (d, *J* = 6.1 Hz, 6H), 1.04 (d, *J* = 6.0 Hz, 6H), 0.89 (d, *J* = 6.1 Hz, 6H). ppm; ^13^C NMR (101 MHz, CDCl_3_): *δ* 158.7 (d, *J* = 3.0 Hz), 157.3, 137.4 (d, *J* = 5.8 Hz), 133.7 (d, *J* = 1.9 Hz), 129.8, 124.2 (d, *J* = 9.4 Hz), 118.1, 113.3 (d, *J* = 105.7 Hz), 111.0 (d, *J* = 6.7 Hz), 104.7 (d, *J* = 6.6 Hz), 71.0, 69.5, 21.6, 21.5 ppm; ^31^P NMR (162 MHz, CDCl_3_) *δ* 6.0 ppm. HRMS (ESI): *m*/*z* calcd. for C_32_H_39_O_5_P [M + H]^+^ 535.2608, found 535.2604.

*Bis(2,7-diisobutoxynaphthalen-1-yl)phosphine oxide* (**2f**). The general procedure was employed, and the product was purified by silica gel (200–300 mesh) column chromatography using petroleum ether/ethyl acetate (3:1 *v*/*v*) as eluent to obtain **2f** as a white solid. ^1^H NMR (400 MHz, CDCl_3_): *δ* 9.91 (s, 0.5H), 8.55 (s, 0.5H), 8.35 (s, 2H), 7.81 (d, *J* = 8.9 Hz, 2H), 7.61 (dd, *J* = 9.0, 1.6 Hz, 2H), 6.98–6.93 (m, 4H), 3.76 (dd, *J* = 8.8, 6.5 Hz, 2H), 3.66 (dd, *J* = 8.8, 6.3 Hz, 2H), 3.52 (dd, *J* = 9.0, 6.2 Hz, 2H), 3.17–2.98 (m, 2H), 1.95–1.82 (m, 4H), 0.91–0.87 (m, 18H), 0.84 (d, *J* = 6.7 Hz, 6H) ppm; ^13^C NMR (101 MHz, CDCl_3_): *δ* 13C NMR (101 MHz, CDCl3) δ 160.0 (d, *J* = 3.0 Hz), 158.8, 137.1, 134.1 (d, *J* = 1.8 Hz), 129.7, 124.3 (d, *J* = 9.4 Hz), 117.6, 113.0, 110.1 (d, *J* = 6.6 Hz), 103.6 (d, *J* = 6.3 Hz), 75.69, 73.91, 28.27, 28.21, 19.24, 19.21, 19.15, 19.11 ppm; ^31^P NMR (162 MHz, CDCl_3_) *δ* 5.8 ppm. HRMS (ESI): *m*/*z* calcd. for C_36_H_47_O_5_P [M + H]^+^ 591.3234, found 591.3175.

*Bis(2,7-bis(cyclopentyloxy)naphthalen-1-yl)phosphine* oxide (**2g**). The general procedure was employed, and the product was purified by silica gel (200–300 mesh) column chromatography using petroleum ether/ethyl acetate (3:1 *v*/*v*) as eluent to obtain **2g** as a white solid. ^1^H NMR (400 MHz, CDCl_3_): *δ* 9.72 (s, 0.5H), 8.64–8.11 (m, 2H + 0.5H), 7.79 (d, *J* = 8.9 Hz, 2H), 7.60 (d, *J* = 8.9 Hz, 2H), 6.96 (dd, *J* = 9.1, 5.0 Hz, 2H), 6.90 (dd, *J* = 8.8, 2.4 Hz, 2H), 4.80 (t, *J* = 6.0 Hz, 2H), 4.42 (s, 2H), 1.87–1.63 (m, 16H), 1.54–1.21 (m, 16H) ppm; ^13^C NMR (101 MHz, CDCl_3_): *δ* 158.9 (d, *J* = 2.9 Hz), 157.6, 133.7 (d, *J* = 1.9 Hz), 129.7, 124.1 (d, *J* = 9.5 Hz), 117.9, 110.7 (d, *J* = 6.9 Hz), 105.0 (d, *J* = 6.7 Hz), 80.4, 79.0, 32.74, 32.67, 32.60, 32.56, 24.1, 23.88, 23.85 ppm; ^31^P NMR (162 MHz, CDCl_3_) *δ* 6.9 ppm. HRMS (ESI): *m*/*z* calcd. for C_40_H_47_O_5_P [M + Na]^+^ 661.3054, found 661.3039.

### 3.3. General Procedure for the Synthesis of Products ***4*** and ***5***

To a 10 mL Schlenk tube were added sulfonamide **1** (0.12 mmol, 1.2 equiv), phosphine oxide **2** (0.1 mmol, 1.0 equiv), Pd(OAc)_2_ (2.2 mg, 10 mol%), dppp (8.2 mg, 20 mol%), Cs_2_CO_3_ (65.2 mg, 0.2 mmol, 2.0 equiv), and toluene (2 mL). The mixture was stirred at 120 °C for 24 h under argon. After cooling to room temperature, 3 mL H_2_O was added to the mixture and extracted with EtOAc. The combined organic layers were dried over MgSO_4_ and filtered. After prep TLC, the crude product **3** was dissolved in THF, and *m*-CPBA (0.15 mmol, 1.5 equiv) was subsequently added at room temperature. After being stirred for 20 min and monitored by TLC, the reaction was quenched with a saturated solution of NaHSO_3_ (5 mL) and extracted with EtOAc. The combined organic layers were washed with brine, dried over MgSO_4_, and filtered. The residue was purified after concentration on silica gel (n-hexane/EA = 2:1) to obtain the desired product **4**. And the products of the opposite configuration **5** were obtained by the same procedure.

*N-(2-(Bis(2,7-dimethoxynaphthalen-1-yl)phosphoryl)benzyl)-2-methylpropane-2-sulfonamide* (**4a**). The general procedure was employed, and the product was purified by silica gel (200–300 mesh) column chromatography using petroleum ether/ethyl acetate (2:1 *v*/*v*) as eluent to obtain **4a** as a white solid (28.5 mg, 44% yield). mp: 115–117 °C; ${\left[\alpha \right]}_{D}^{25}$ = 1.8 (*c* = 0.1, CH_2_Cl_2_). ^1^H NMR (400 MHz, CDCl_3_): *δ* 8.66 (s, 1H), 8.33 (s, 1H), 7.87 (dd, *J* = 15.5, 8.9 Hz, 2H), 7.68 (d, *J* = 9.0 Hz, 1H), 7.62 (t, *J* = 7.7 Hz, 2H), 7.42 (t, *J* = 7.5 Hz, 1H), 7.30 (dd, *J* = 15.5, 7.7 Hz, 1H), 7.19–7.14 (m, 2H), 7.04–6.96 (m, 2H), 6.95–6.88 (m, 2H), 4.61 (dd, *J* = 13.8, 3.9 Hz, 1H), 4.38 (dd, *J* = 13.9, 8.8 Hz, 1H), 3.69 (s, 3H), 3.34 (s, 3H), 3.30 (s, 3H), 3.13 (s, 3H), 1.30 (s, 9H) ppm; ^13^C NMR (101 MHz, CDCl_3_): *δ* 161.1, 159.2, 158.8, 158.5, 143.0 (d, *J* = 8.3 Hz), 137.7 (d, *J* = 6.1 Hz), 136.7 (d, *J* = 5.9 Hz), 136.4, 135.4, 134.9, 133.9, 132.2 (d, *J* = 13.6 Hz), 131.7 (d, *J* = 10.3 Hz), 131.1 (d, *J* = 2.9 Hz), 129.9, 129.6, 126.5 (d, *J* = 13.7 Hz), 125.0 (t, *J* = 11.7 Hz), 117.2 (d, *J* = 22.2 Hz), 115.7 (d, *J* = 107.8 Hz), 112.3 (d, *J* = 105.4 Hz), 110.7 (d, *J* = 6.9 Hz), 109.9 (d, *J* = 7.7 Hz), 105.3, 104.5 (d, *J* = 5.9 Hz), 59.5, 55.9, 55.4, 55.3, 54.8, 48.0 (d, *J* = 5.5 Hz), 24.3 ppm; ^31^P NMR (162 MHz, CDCl_3_) *δ* 33.7 ppm. HRMS (ESI): *m*/*z* calcd. for C_35_H_38_NO_7_PS [M + Na]^+^ 670.1999, found 670.1989.

*N-(2-(Bis(2,7-diethoxynaphthalen-1-yl)phosphoryl)benzyl)-2-methylpropane-2-sulfonamide* (**4b**). The general procedure was employed, and the product was purified by silica gel (200–300 mesh) column chromatography using petroleum ether/ethyl acetate (2:1 *v*/*v*) as eluent to obtain **4b** as a white solid (26.1 mg, 37% yield). mp: 124–126 °C; ${\left[\alpha \right]}_{D}^{25}\;$= 0.9 (*c* = 0.1, CH_2_Cl_2_). ^1^H NMR (400 MHz, CDCl_3_): *δ* 8.80 (d, *J* = 2.4 Hz, 1H), 8.18 (d, *J* = 2.4 Hz, 1H), 7.83 (dd, *J* = 15.5, 9.0 Hz, 2H), 7.66 (dd, *J* = 8.9, 1.7 Hz, 1H), 7.61–7.56 (m, 2H), 7.43–7.38 (m, 1H), 7.38–7.31 (m, 1H), 7.18–7.09 (m, 2H), 7.02 (dd, *J* = 8.9, 2.4 Hz, 1H), 6.94 (dd, *J* = 8.9, 5.3 Hz, 1H), 6.91–6.86 (m, 2H), 4.60 (dd, *J* = 13.9, 4.1 Hz, 1H), 4.39 (dd, *J* = 13.8, 8.7 Hz, 1H), 4.09–4.02 (m, 1H), 3.90–3.82 (m, 2H), 3.78–3.70 (m, 2H), 3.61–3.47 (m, 2H), 3.13–3.05 (m, 1H), 1.34 (t, *J* = 7.0 Hz, 3H), 1.30 (s, 9H), 1.07 (t, *J* = 7.0 Hz, 3H), 0.66 (t, *J* = 7.0 Hz, 3H), 0.55 (t, *J* = 7.0 Hz, 3H) ppm; ^13^C NMR (101 MHz, CDCl_3_): *δ* 13C NMR (101 MHz, CDCl3) δ 159.7 (d, *J* = 2.9 Hz), 158.4, 137.8, 137.2 (d, *J* = 5.5 Hz), 134.0, 133.0, 130.2, 129.8, 129.3, 127.6, 124.3 (d, *J* = 9.3 Hz), 123.9, 117.3, 112.6 (d, *J* = 104.3 Hz), 110.3 (d, *J* = 6.6 Hz), 103.8 (d, *J* = 6.6 Hz), 64.8, 63.3, 56.1, 49.7, 22.6, 14.5, 14.3 ppm; ^31^P NMR (162 MHz, CDCl_3_) *δ* 33.8 ppm. HRMS (ESI): *m*/*z* calcd. for C_39_H_46_NO_7_PS [M + Na]^+^ 726.2625, found 726.2578.

*N-(2-(bis(2,7-dipropoxynaphthalen-1-yl)phosphoryl)benzyl)-2-methylpropane-2-sulfonamide* (**4c**). The general procedure was employed, and the product was purified by silica gel (200–300 mesh) column chromatography using petroleum ether/ethyl acetate (2:1 *v*/*v*) as eluent to obtain **4c** as a white solid (21.2 mg, 28% yield). mp: 128–130 °C; ${\left[\alpha \right]}_{D}^{25}$ = −1.8 (*c* = 0.1, CH_2_Cl_2_). ^1^H NMR (400 MHz, CDCl_3_): *δ* 8.80 (s, 1H), 8.14 (s, 1H), 7.82 (dd, *J* = 13.3, 9.0 Hz, 2H), 7.66 (d, *J* = 8.9 Hz, 1H), 7.58 (d, *J* = 9.2 Hz, 2H), 7.44–7.30 (m, 2H), 7.11 (t, *J* = 7.9 Hz, 2H), 7.02 (d, *J* = 7.9 Hz, 1H), 6.96 (dd, *J* = 9.0, 5.2 Hz, 1H), 6.88 (d, *J* = 8.6 Hz, 2H), 4.59 (dd, *J* = 13.7, 4.2 Hz, 1H), 4.39 (dd, *J* = 13.8, 8.4 Hz, 1H), 3.98–3.89 (m, 1H), 3.80–3.69 (m, 2H), 3.63–3.54 (m, 2H), 3.51–3.44 (m, 1H), 3.35–3.26 (m, 1H), 2.94–2.84 (m, 1H), 1.79–1.68 (m, 4H), 1.53–1.45 (m, 2H), 1.30 (s, 9H), 1.00–0.93 (m, 5H), 0.78 (t, *J* = 7.4 Hz, 3H), 0.61 (t, *J* = 7.4 Hz, 3H), 0.49 (t, *J* = 7.5 Hz, 3H) ppm; ^13^C NMR (101 MHz, CDCl_3_): *δ* 160.4, 158.8, 158.4, 157.9, 143.2 (d, *J* = 8.1 Hz), 138.0 (d, *J* = 6.2 Hz), 136.8 (d, *J* = 6.5 Hz), 135.8, 134.6, 133.7, 132.7 (d, *J* = 13.7 Hz), 132.0 (d, *J* = 10.3 Hz), 131.0, 129.7, 129.5, 126.4 (d, *J* = 13.5 Hz), 124.8, 124.7 (d, *J* = 5.9 Hz), 124.6, 117.4, 117.1, 115.1 (d, *J* = 109.4 Hz), 110.5 (d, *J* = 7.4 Hz), 109.9 (d, *J* = 7.9 Hz), 106.0, 105.3 (d, *J* = 6.3 Hz), 70.4, 70.2, 69.4, 69.0, 59.5, 48.1, 24.3, 22.33, 22.26, 21.64, 21.57, 10.6, 10.3, 9.9 ppm; ^31^P NMR (162 MHz, CDCl_3_) *δ* 33.6 ppm. HRMS (ESI): *m*/*z* calcd. for C_43_H_54_NO_7_PS [M + H]^+^ 760.3432, found 760.3391.

*N-(2-(Bis(2,7-dibutoxynaphthalen-1-yl)phosphoryl)benzyl)-2-methylpropane-2-sulfonamide* (**4d**). The general procedure was employed, and the product was purified by silica gel (200–300 mesh) column chromatography using petroleum ether/ethyl acetate (2:1 *v*/*v*) as eluent to obtain **4d** as a white solid (27.7 mg, 34% yield). mp: 136–138 °C; ${\left[\alpha \right]}_{D}^{25}$ = −0.6 (*c* = 0.3, CH_2_Cl_2_). ^1^H NMR (400 MHz, CDCl_3_): *δ* 8.74 (d, *J* = 2.4 Hz, 1H), 8.19 (d, *J* = 2.4 Hz, 1H), 7.82 (dd, *J* = 12.2, 9.0 Hz, 2H), 7.65 (dd, J = 9.0, 1.7 Hz, 1H), 7.61–7.55 (m, 2H), 7.40 (t, *J* = 7.5 Hz, 1H), 7.33 (dd, *J* = 15.4, 7.8 Hz, 1H), 7.15–7.07 (m, 2H), 7.01 (dd, *J* = 8.9, 2.4 Hz, 1H), 6.96 (dd, *J* = 9.0, 5.2 Hz, 1H), 6.91–6.85 (m, 2H), 4.57 (dd, *J* = 13.7, 4.2 Hz, 1H), 4.38 (dd, *J* = 13.7, 8.4 Hz, 1H), 3.98–3.91 (m, 1H), 3.80–3.72 (m, 2H), 3.68–3.60 (m, 2H), 3.55–3.49 (m, 1H), 3.40–3.34 (m, 1H), 3.02–2.94 (m, 1H), 1.75–1.61 (m, 4H), 1.48–1.41 (m, 4H), 1.29 (s, 9H), 1.26–1.21 (m, 2H), 1.06–0.96 (m, 3H), 0.92 (t, *J* = 7.3 Hz, 3H), 0.89–0.86 (m, 3H), 0.82 (t, *J* = 7.4 Hz, 3H), 0.73 (t, *J* = 7.0 Hz, 3H), 0.60 (t, *J* = 7.1 Hz, 3H) ppm; ^13^C NMR (101 MHz, CDCl_3_): *δ* 160.4, 158.8, 158.4, 157.9, 143.2 (d, *J* = 8.5 Hz), 137.9 (d, *J* = 6.3 Hz), 136.9 (d, *J* = 6.6 Hz), 134.6, 133.6, 132.7 (d, *J* = 13.4 Hz), 132.1 (d, *J* = 10.2 Hz), 131.0, 129.7, 129.4, 126.3 (d, *J* = 13.4 Hz), 124.7 (t, *J* = 9.9 Hz), 117.4, 117.1, 114.6, 110.5 (d, *J* = 7.4 Hz), 109.9 (d, *J* = 8.1 Hz), 106.0, 105.3, 68.7, 68.4, 67.6, 67.2, 59.5, 48.1, 31.1 (d, *J* = 2.8 Hz), 30.4 (d, *J* = 5.9 Hz), 24.4, 19.3, 19.0, 18.7, 13.8 (d, *J* = 6.2 Hz), 13.7, 13.5 ppm; ^31^P NMR (162 MHz, CDCl_3_) *δ* 34.4 ppm. HRMS (ESI): *m*/*z* calcd. for C_47_H_62_NO_7_PS [M + H]^+^ 816.4058, found 816.4097.

*N-(2-(Bis(2,7-bis(cyclopentyloxy)naphthalen-1-yl)phosphoryl)benzyl)-2-methylpropane-2-sulfonamide* (**4e**). The general procedure was employed, and the product was purified by silica gel (200–300 mesh) column chromatography using petroleum ether/ethyl acetate (2:1 *v*/*v*) as eluent to obtain **4e** as a white solid (31.9 mg, 37% yield). mp: 144–146 °C; ${\left[\alpha \right]}_{D}^{25}$ = 0.45 (*c* = 0.4, CH_2_Cl_2_). ^1^H NMR (400 MHz, CDCl_3_): *δ* 8.57 (d, *J* = 2.4 Hz, 1H), 8.13 (d, *J* = 2.3 Hz, 1H), 7.80 (dd, *J* = 12.1, 9.0 Hz, 2H), 7.62 (dd, *J* = 9.0, 1.7 Hz, 1H), 7.58–7.54 (m, 2H), 7.39–7.30 (m, 2H), 7.24 (dd, *J* = 8.6, 4.0 Hz, 1H), 7.11–7.06 (m, 1H), 6.95–6.91 (m, 2H), 6.88 (dd, *J* = 9.0, 4.9 Hz, 1H), 6.84 (dd, *J* = 8.9, 2.4 Hz, 1H), 4.74–4.69 (m, 1H), 4.60–4.50 (m, 3H), 4.34 (dd, *J* = 13.6, 8.7 Hz, 1H), 3.96–3.91 (m, 1H), 2.03–1.96 (m, 1H), 1.85–1.46 (m, 18H), 1.40–1.33 (m, 5H), 1.31 (s, 9H), 1.22–0.92 (m, 6H), 0.80–0.66 (m, 2H) ppm; ^13^C NMR (101 MHz, CDCl_3_): *δ* 159.5, 158.0 (d, *J* = 3.0 Hz), 157.2, 156.8, 143.1 (d, *J* = 7.2 Hz), 137.8 (d, *J* = 6.2 Hz), 137.0 (d, *J* = 6.4 Hz), 136.9, 135.9, 134.2, 133.3, 133.1, 132.2 (d, *J* = 10.4 Hz), 130.9, 129.5 (d, *J* = 16.9 Hz), 126.3 (d, *J* = 13.4 Hz), 124.4, 124.2 (d, *J* = 6.9 Hz), 124.1, 117.8, 117.4, 114.7 (d, *J* = 109.7 Hz), 111.5 (d, *J* = 105.1 Hz), 110.7 (d, *J* = 7.5 Hz), 110.5 (d, *J* = 8.0 Hz), 107.7 (d, *J* = 4.5 Hz), 106.8 (d, *J* = 6.6 Hz), 79.7 (d, *J* = 15.6 Hz), 79.4, 78.8, 59.7, 48.5 (d, *J* = 5.3 Hz), 32.9, 32.7, 32.5, 32.2 (d, *J* = 2.4 Hz), 32.0, 31.7 (d, *J* = 5.5 Hz), 24.4, 24.3, 24.0 (d, *J* = 3.6 Hz), 23.9 (d, *J* = 3.6 Hz), 23.8 ppm; ^31^P NMR (162 MHz, CDCl_3_) *δ* 32.8 ppm. HRMS (ESI): *m*/*z* calcd. for C_51_H_62_NO_7_PS [M + Na]^+^ 886.3877, found 886.3941.

*N-(2-(Bis(2,7-diisopropoxynaphthalen-1-yl)phosphoryl)benzyl)-2-methylpropane-2-sulfonamide* (**4f**). The general procedure was employed, and the product was purified by silica gel (200–300 mesh) column chromatography using petroleum ether/ethyl acetate (2:1 *v*/*v*) as eluent to obtain **4f** as a white solid (44.8 mg, 59% yield). mp: 96–98 °C; ${\left[\alpha \right]}_{D}^{25}$ = −1.36 (*c* = 0.5, CH_2_Cl_2_). ^1^H NMR (400 MHz, CDCl_3_): *δ* 8.71 (d, *J* = 2.4 Hz, 1H), 8.10 (d, *J* = 2.3 Hz, 1H), 7.80 (t, *J* = 9.8 Hz, 2H), 7.65–7.60 (m, 2H), 7.56 (dd, *J* = 8.9, 1.7 Hz, 1H), 7.43–7.30 (m, 3H), 7.14–7.07 (m, 1H), 6.97 (dd, *J* = 8.9, 2.4 Hz, 1H), 6.92–6.85 (m, 2H), 6.82 (dd, *J* = 8.9, 2.4 Hz, 1H), 4.63 (dd, *J* = 13.5, 3.4 Hz, 1H), 4.54 (p, *J* = 6.0 Hz, 1H), 4.44 (p, *J* = 6.3 Hz, 2H), 4.33 (dd, *J* = 13.5, 9.3 Hz, 1H), 3.76 (p, *J* = 6.0 Hz, 1H), 1.37 (d, *J* = 6.0 Hz, 3H), 1.31 (s, 9H), 1.11 (d, *J* = 6.0 Hz, 3H), 1.06 (d, *J* = 6.0 Hz, 3H), 0.97 (d, *J* = 5.9 Hz, 3H), 0.83 (d, *J* = 6.1 Hz, 3H), 0.71 (d, *J* = 6.0 Hz, 3H), 0.59 (d, *J* = 6.1 Hz, 3H), 0.24 (d, *J* = 6.0 Hz, 3H) ppm; ^13^C NMR (101 MHz, CDCl_3_): *δ* 159.0 (d, *J* = 2.6 Hz), 157.1 (d, *J* = 3.0 Hz), 156.9, 156.5, 143.2 (d, *J* = 8.3 Hz), 138.1 (d, *J* = 6.2 Hz), 137.1 (d, *J* = 6.3 Hz), 136.1, 134.4, 133.3 (d, *J* = 2.2 Hz), 133.0 (d, *J* = 14.0 Hz), 132.2 (d, *J* = 10.3 Hz), 131.1 (d, *J* = 2.9 Hz), 129.5 (d, *J* = 20.4 Hz), 126.6 (d, *J* = 13.7 Hz), 124.4 (d, *J* = 9.6 Hz), 124.2 (d, *J* = 9.8 Hz), 117.8, 117.5, 115.3 (d, *J* = 109.8 Hz), 111.6 (d, *J* = 105.3 Hz), 110.1 (d, *J* = 7.5 Hz), 109.7 (d, *J* = 7.9 Hz), 107.2 (d, *J* = 4.5 Hz), 106.8 (d, *J* = 6.7 Hz), 69.8, 69.3 (d, *J* = 2.7 Hz), 68.7, 59.6, 48.7 (d, *J* = 5.4 Hz), 24.4, 21.8, 21.6 (d, *J* = 6.3 Hz), 21.3, 21.1 (d, *J* = 3.7 Hz), 20.8, 20.0 ppm; ^31^P NMR (162 MHz, CDCl_3_) *δ* 33.4 ppm. HRMS (ESI): *m*/*z* calcd. for C_43_H_54_NO_7_PS [M + H]^+^ 760.3432, found 760.3312.

*N-(2-(Bis(2,7-diisobutoxynaphthalen-1-yl)phosphoryl)benzyl)-2-methylpropane-2-sulfonamide* (**4g**). The general procedure was employed, and the product was purified by silica gel (200–300 mesh) column chromatography using petroleum ether/ethyl acetate (2:1 *v*/*v*) as eluent to obtain **4g** as a white solid (26.9 mg, 33% yield). mp: 92–94 °C; ${\left[\alpha \right]}_{D}^{25}$ = 0.36 (*c* = 0.5, CH_2_Cl_2_). ^1^H NMR (400 MHz, CDCl_3_): *δ* 8.69 (d, *J* = 2.4 Hz, 1H), 8.16 (d, *J* = 2.4 Hz, 1H), 7.82 (t, *J* = 8.6 Hz, 2H), 7.65 (dd, *J* = 9.0, 1.7 Hz, 1H), 7.61–7.56 (m, 2H), 7.42–7.30 (m, 2H), 7.15–7.05 (m, 2H), 7.02 (dd, *J* = 8.9, 2.4 Hz, 1H), 6.97 (dd, *J* = 9.0, 5.2 Hz, 1H), 6.92–6.86 (m, 2H), 4.53 (dd, *J* = 13.6, 4.8 Hz, 1H), 4.37 (dd, *J* = 13.7, 7.9 Hz, 1H), 3.68 (dd, *J* = 9.3, 6.4 Hz, 1H), 3.55–3.47 (m, 2H), 3.40–3.25 (m, 3H), 3.01 (dd, *J* = 9.7, 6.7 Hz, 1H), 2.72 (dd, *J* = 9.1, 6.5 Hz, 1H), 2.02–1.93 (m, 1H), 1.82–1.77 (m, 1H), 1.30 (s, 9H), 1.24–1.18 (m, 1H), 0.96 (dd, *J* = 6.7, 4.7 Hz, 6H), 0.78 (dd, *J* = 8.0, 6.7 Hz, 6H), 0.68 (d, *J* = 6.7 Hz, 3H), 0.52 (d, *J* = 6.6 Hz, 3H), 0.48 (d, *J* = 6.6 Hz, 3H), 0.37 (d, *J* = 6.7 Hz, 3H) ppm; ^13^C NMR (101 MHz, CDCl_3_): *δ* 160.7, 159.2 (d, *J* = 2.7 Hz), 158.6, 158.1, 143.2 (d, *J* = 8.3 Hz), 137.7 (d, *J* = 6.4 Hz), 136.9 (d, *J* = 6.7 Hz), 135.8, 134.6, 133.7, 132.9 (d, *J* = 13.5 Hz), 132.2 (d, *J* = 10.2 Hz), 131.1, 129.7, 129.4, 126.4 (d, *J* = 13.6 Hz), 124.8 (t, *J* = 9.7 Hz), 117.6, 117.1, 115.0 (d, *J* = 109.7 Hz), 111.1 (d, *J* = 7.3 Hz), 110.5 (d, *J* = 7.8 Hz), 106.2 (d, *J* = 4.5 Hz), 105.4 (d, *J* = 6.3 Hz), 76.0, 75.9, 74.0, 73.7, 59.5, 48.1 (d, *J* = 5.5 Hz), 28.2, 28.0, 27.8, 27.6, 24.4, 19.3 (d, *J* = 24.4 Hz), 19.1 (d, *J* = 1.9 Hz), 19.0, 18.7 ppm; ^31^P NMR (162 MHz, CDCl_3_) *δ* 33.1 ppm. HRMS (ESI): *m*/*z* calcd. for C_47_H_62_NO_7_PS [M + H]^+^ 816.4058, found 816.4100.

*N-(2-(Bis(2,7-dimethoxynaphthalen-1-yl)phosphoryl)-5-methylbenzyl)-2-methylpropane-2-sulfonamide* (**4h**). The general procedure was employed, and the product was purified by silica gel (200–300 mesh) column chromatography using petroleum ether/ethyl acetate (2:1 *v*/*v*) as eluent to obtain **4h** as a white solid (27.1 mg, 41% yield). mp: 135–137 °C; ${\left[\alpha \right]}_{D}^{25}$ = 0.9 (*c* = 0.2, CH_2_Cl_2_). ^1^H NMR (400 MHz, CDCl_3_): *δ* 8.64 (s, 1H), 8.32 (s, 1H), 7.86 (dd, *J* = 14.8, 8.5 Hz, 2H), 7.67 (d, *J* = 9.3 Hz, 1H), 7.61 (d, *J* = 8.9 Hz, 1H), 7.44 (d, *J* = 2.7 Hz, 1H), 7.22–7.14 (m, 2H), 7.03–6.89 (m, 5H), 4.57 (dd, *J* = 13.8, 4.0 Hz, 1H), 4.33 (dd, *J* = 13.7, 8.7 Hz, 1H), 3.69 (s, 3H), 3.35 (s, 3H), 3.29 (s, 3H), 3.12 (s, 3H), 2.35 (s, 3H), 1.30 (s, 9H) ppm; ^13^C NMR (101 MHz, CDCl_3_): *δ* 160.5, 159.1, 137.6, 137.4, 137.06 (d, *J* = 5.5 Hz), 134.2 (d, *J* = 1.9 Hz), 132.7, 131.1, 130.1, 130.0, 124.6 (d, *J* = 9.4 Hz), 120.5, 117.2, 113.3, 112.3, 109.8 (d, *J* = 6.5 Hz), 103.3 (d, *J* = 6.9 Hz), 56.3, 56.1, 55.2, 49.7, 22.6, 20.9 ppm; ^31^P NMR (162 MHz, CDCl_3_) *δ* 33.5 ppm. HRMS (ESI): *m*/*z* calcd. for C_36_H_40_NO_7_PS [M + Na]^+^ 684.2156, found 684.2166.

*N-(2-(Bis(2,7-dimethoxynaphthalen-1-yl)phosphoryl)-5-(trifluoromethyl)benzyl)-2-methylpropane-2-sulfonamide* (**4i**). The general procedure was employed, and the product was purified by silica gel (200–300 mesh) column chromatography using petroleum ether/ethyl acetate (2:1 *v*/*v*) as eluent to obtain **4i** as a white solid (39.4 mg, 55% yield). mp: 163–165 °C; ${\left[\alpha \right]}_{D}^{25}$ = −0.18 (*c* = 1.0, CH_2_Cl_2_). ^1^H NMR (400 MHz, CDCl_3_): *δ* 8.77 (s, 1H), 8.12 (s, 1H), 7.93–7.85 (m, 3H), 7.70 (d, *J* = 9.0 Hz, 1H), 7.61 (d, *J* = 8.9 Hz, 1H), 7.45–7.39 (m, 2H), 7.10–7.03 (m, 2H), 6.99 (dd, *J* = 9.0, 5.4 Hz, 1H), 6.95–6.88 (m, 2H), 4.69 (dd, *J* = 14.0, 3.9 Hz, 1H), 4.49–4.41 (m, 1H), 3.75 (s, 3H), 3.37 (s, 3H), 3.22 (s, 3H), 3.14 (s, 3H), 1.30 (s, 9H) ppm; ^13^C NMR (101 MHz, CDCl_3_): *δ* 161.1, 159.2, 159.0, 158.7, 144.0 (d, *J* = 8.8 Hz), 140.7, 139.6, 137.8, 136.4, 135.5, 134.3, 133.3, 132.8, 132.4 (d, *J* = 14.4 Hz), 130.0 (d, *J* = 31.5 Hz), 129.7, 128.2 (q, *J*_C-F_ = 3.4 Hz), 127.8, 125.0, 123.2, 122.3, 117.4 (d, *J* = 21.2 Hz), 110.0 (d, *J* = 85.6 Hz), 104.6 (d, *J* = 70.6 Hz), 104.0, 75.6, 68.6, 59.7, 55.7, 55.3, 54.7, 47.5 (d, *J* = 4.5 Hz), 24.2 ppm; ^19^F NMR (376 MHz, CDCl_3_) *δ* –62.5 ppm; ^31^P NMR (162 MHz, CDCl_3_) *δ* 33.2 ppm. HRMS (ESI): *m*/*z* calcd. for C_36_H_37_F_3_NO_7_PS [M + Na]^+^ 738.1873, found 738.1860.

*N-(2-(Bis(2,7-dimethoxynaphthalen-1-yl)phosphoryl)-5-methoxybenzyl)-2-methylpropane-2-sulfonamide* (**4j**). The general procedure was employed, and the product was purified by silica gel (200–300 mesh) column chromatography using petroleum ether/ethyl acetate (2:1 *v*/*v*) as eluent to obtain **4j** as a white solid (21.7 mg, 32% yield). mp: 121–123 °C; ${\left[\alpha \right]}_{D}^{25}$ = 0.9 (*c* = 0.2, CH_2_Cl_2_). ^1^H NMR (400 MHz, CDCl_3_): *δ* 8.66 (s, 1H), 8.26 (s, 1H), 7.86 (dd, *J* = 14.6, 8.9 Hz, 2H), 7.68 (d, *J* = 9.0 Hz, 1H), 7.60 (d, *J* = 8.9 Hz, 1H), 7.25–7.18 (m, 2H), 7.11 (t, *J* = 6.5 Hz, 1H), 7.04–6.97 (m, 2H), 6.91 (d, *J* = 8.3 Hz, 2H), 6.70–6.63 (m, 1H), 4.62 (dd, *J* = 14.4, 4.1 Hz, 1H), 4.35 (dd, *J* = 14.2, 8.8 Hz, 1H), 3.83 (s, 3H), 3.72 (s, 3H), 3.38 (s, 3H), 3.27 (s, 3H), 3.11 (s, 3H), 1.31 (s, 9H) ppm; ^13^C NMR (101 MHz, CDCl_3_): *δ* 160.5 (d, *J* = 2.8 Hz), 159.1, 138.8, 137.0 (d, *J* = 5.5 Hz), 134.2 (d, *J* = 2.0 Hz), 133.5, 123.0, 124.6 (d, *J* = 9.4 Hz), 117.1, 115.8, 114.8, 113.9, 112.7 (d, *J* = 104.7 Hz), 109.8 (d, *J* = 6.7 Hz), 103.3 (d, *J* = 6.8 Hz), 56.3, 56.1, 55.5, 55.2, 49.8, 22.6 ppm; ^31^P NMR (162 MHz, CDCl_3_) *δ* 32.9 ppm. HRMS (ESI): *m*/*z* calcd. for C_36_H_40_NO_8_PS [M + Na]^+^ 700.2105, found 700.2100.

*N-(2-(Bis(2,7-dimethoxynaphthalen-1-yl)phosphoryl)-5-ethoxybenzyl)-2-methylpropane-2-sulfonamide* (**4k**). The general procedure was employed, and the product was purified by silica gel (200–300 mesh) column chromatography using petroleum ether/ethyl acetate (2:1 *v*/*v*) as eluent to obtain **4k** as a white solid (27.7 mg, 40% yield). mp: 125–127 °C; ${\left[\alpha \right]}_{D}^{25}$ = 1.8 (*c* = 0.1, CH_2_Cl_2_). ^1^H NMR (400 MHz, CDCl_3_): *δ* 8.66 (s, 1H), 8.32 (s, 1H), 7.89–7.82 (m, 2H), 7.68 (d, *J* = 8.9 Hz, 1H), 7.60 (d, *J* = 8.9 Hz, 1H), 7.23–7.16 (m, 3H), 7.04–6.97 (m, 2H), 6.91 (d, *J* = 8.2 Hz, 2H), 6.64 (d, *J* = 8.7 Hz, 1H), 4.58 (dd, *J* = 14.0, 3.9 Hz, 1H), 4.32 (dd, *J* = 13.9, 8.7 Hz, 1H), 4.10–4.02 (m, 2H), 3.72 (s, 3H), 3.38 (s, 3H), 3.29 (s, 3H), 3.11 (s, 3H), 1.40 (t, *J* = 7.0 Hz, 3H), 1.31 (s, 9H) ppm; ^13^C NMR (101 MHz, CDCl_3_): *δ* 160.5, 159.1, 158.5, 138.7, 137.0, 134.2, 133.5, 130.0, 124.6 (d, *J* = 9.6 Hz), 117.2, 116.4, 115.4, 113.7, 109.9 (d, *J* = 6.7 Hz), 103.3 (d, *J* = 6.8 Hz), 63.8, 56.3, 56.1, 55.2, 49.9, 22.6, 14.7 ppm; ^31^P NMR (162 MHz, CDCl_3_) *δ* 34.5 ppm. HRMS (ESI): *m*/*z* calcd. for C_37_H_42_NO_8_PS [M + H]^+^ 692.2442, found 692.2423.

*N-(2-(Bis(2,7-dimethoxynaphthalen-1-yl)phosphoryl)-5-propoxybenzyl)-2-methylpropane-2-sulfonamide* (**4l**). The general procedure was employed, and the product was purified by silica gel (200–300 mesh) column chromatography using petroleum ether/ethyl acetate (2:1 *v*/*v*) as eluent to obtain **4l** as a white solid (33.2 mg, 47% yield). mp: 146–148 °C; ${\left[\alpha \right]}_{D}^{25}$ = 1.8 (*c* = 0.1, CH_2_Cl_2_). ^1^H NMR (400 MHz, CDCl_3_): *δ* 8.65 (s, 1H), 8.32 (s, 1H), 7.85 (dd, *J* = 14.5, 8.9 Hz, 2H), 7.67 (d, *J* = 9.2 Hz, 1H), 7.60 (d, *J* = 8.9 Hz, 1H), 7.24–7.14 (m, 3H), 7.05–6.96 (m, 2H), 6.90 (dd, *J* = 9.0, 4.9 Hz, 2H), 6.65 (dt, *J* = 8.6, 2.3 Hz, 1H), 4.59 (dd, *J* = 13.8, 3.9 Hz, 1H), 4.36–4.30 (m, 1H), 3.99–3.91 (m, 2H), 3.72 (s, 3H), 3.38 (s, 3H), 3.29 (s, 3H), 3.11 (s, 3H), 1.82–1.76 (m, 2H), 1.31 (s, 9H), 1.02 (t, *J* = 7.4 Hz, 3H) ppm; ^13^C NMR (101 MHz, CDCl_3_): *δ* 160.5 (d, *J* = 3.0 Hz), 159.1, 158.7, 138.7, 137.1 (d, *J* = 5.6 Hz), 134.2 (d, *J* = 2.0 Hz), 133.5, 130.0, 124.6 (d, *J* = 9.2 Hz), 117.2, 116.4, 115.4, 113.7, 113.3, 112.3, 109.8 (d, *J* = 6.6 Hz), 103.3 (d, *J* = 6.7 Hz), 69.8, 56.3, 56.1, 55.2, 49.8, 22.6, 22.5, 10.4 ppm; ^31^P NMR (162 MHz, CDCl_3_) *δ* 32.8 ppm. HRMS (ESI): *m*/*z* calcd. for C_38_H_44_NO_8_PS [M + Na]^+^ 728.2418, found 728.2407.

*N-(2-(Bis(2,7-dimethoxynaphthalen-1-yl)phosphoryl)-5-isopropoxybenzyl)-2-methylpropane-2-sulfonamide* (**4m**). The general procedure was employed, and the product was purified by silica gel (200–300 mesh) column chromatography using petroleum ether/ethyl acetate (2:1 *v*/*v*) as eluent to obtain **4m** as a white solid (38.8 mg, 55% yield). mp: 115–117 °C; ${\left[\alpha \right]}_{D}^{25}$ = 0.18 (*c* = 0.1, CH_2_Cl_2_). ^1^H NMR (400 MHz, CDCl_3_): *δ* 8.63 (s, 1H), 8.36 (s, 1H), 7.86 (dd, *J* = 14.3, 9.0 Hz, 2H), 7.67 (d, *J* = 9.0 Hz, 1H), 7.61 (d, *J* = 9.0 Hz, 1H), 7.23–7.14 (m, 3H), 7.04–6.97 (m, 2H), 6.91 (dd, *J* = 9.1, 4.7 Hz, 2H), 6.63 (d, *J* = 8.5 Hz, 1H), 4.65–4.55 (m, 2H), 4.34–4.28 (m, 1H), 3.71 (s, 3H), 3.38 (s, 3H), 3.31 (s, 3H), 3.12 (s, 3H), 1.35–1.30 (m, 15H) ppm; ^13^C NMR (101 MHz, CDCl_3_): *δ* 160.7 (d, *J* = 3.0 Hz), 159.3, 157.4, 156.8, 151.1, 138.5, 137.0 (d, *J* = 5.3 Hz), 134.6, 134.1, 133.6, 130.0 (d, *J* = 20.4 Hz), 128.9, 124.6 (d, *J* = 9.5 Hz), 124.4, 117.7, 117.3, 116.8, 116.3, 114.1, 113.7, 111.8 (d, *J* = 106.6 Hz), 109.7 (d, *J* = 6.7 Hz), 107.9, 104.5, 103.1 (d, *J* = 7.0 Hz), 70.3, 56.3 (d, *J* = 3.1 Hz), 55.2, 49.9, 29.5, 22.7, 21.9 (d, *J* = 3.6 Hz) ppm; ^31^P NMR (162 MHz, CDCl_3_) *δ* 34.5 ppm. HRMS (ESI): *m*/*z* calcd. for C_38_H_44_NO_8_PS [M + Na]^+^ 728.2418, found 728.2407.

*N-((6-(Bis(2,7-dimethoxynaphthalen-1-yl)phosphoryl)benzo[d][1,3]dioxol-5-yl)methyl)-2-methylpropane-2-sulfonamide* (**4n**). The general procedure was employed, and the product was purified by silica gel (200–300 mesh) column chromatography using petroleum ether/ethyl acetate (2:1 *v*/*v*) as eluent to obtain **4n** as a white solid (22.1 mg, 32% yield). mp: 101–103 °C; ${\left[\alpha \right]}_{D}^{25}$ = 0.45 (*c* = 0.2, CH_2_Cl_2_). ^1^H NMR (400 MHz, CDCl_3_): *δ* 8.72 (s, 1H), 8.20 (s, 1H), 7.86 (dd, *J* = 13.7, 8.8 Hz, 2H), 7.68 (d, *J* = 9.0 Hz, 1H), 7.60 (d, *J* = 8.9 Hz, 1H), 7.21–6.84 (m, 6H), 6.76 (d, *J* = 15.1 Hz, 1H), 5.93 (d, *J* = 20.9 Hz, 2H), 4.57 (d, *J* = 14.0 Hz, 1H), 4.31–4.25 (m, 1H), 3.78 (s, 3H), 3.45 (s, 3H), 3.24 (s, 3H), 3.11 (s, 3H), 1.31 (s, 9H) ppm; ^13^C NMR (101 MHz, CDCl_3_): *δ* 160.9, 159.2, 159.0, 158.5, 149.5, 146.3, 146.1, 139.1 (d, *J* = 9.1 Hz), 134.9, 133.8, 129.8 (d, *J* = 32.6 Hz), 128.8, 125.1 (d, *J* = 10.0 Hz), 117.3 (d, *J* = 24.3 Hz), 112.4, 112.3 (d, *J* = 7.1 Hz), 112.1, 110.8, 110.0, 105.2, 104.7, 101.4, 59.6, 55.8 (d, *J* = 21.2 Hz), 55.4, 54.8, 47.8, 29.7, 24.3, 22.7, 14.1 ppm; ^31^P NMR (162 MHz, CDCl_3_) *δ* 33.0 ppm. HRMS (ESI): *m*/*z* calcd. for C_36_H_38_NO_9_PS [M + Na]^+^ 714.1898, found 714.1900.

*N-(2-(Bis(2,7-dimethoxynaphthalen-1-yl)phosphoryl)benzyl)-2-methylpropane-2-sulfonamide* (**5a**). The general procedure was employed, and the product was purified by silica gel (200–300 mesh) column chromatography using petroleum ether/ethyl acetate (2:1 *v*/*v*) as eluent to obtain **5a** as a white solid (20.1 mg, 31% yield). mp: 97–99 °C; ${\left[\alpha \right]}_{D}^{25}$ = −1.8 (*c* = 0.3, CH_2_Cl_2_). ^1^H NMR (400 MHz, CDCl_3_): *δ* 8.65 (s, 1H), 8.29 (s, 1H), 7.87 (dd, *J* = 15.6, 8.9 Hz, 2H), 7.70–7.59 (m, 3H), 7.45–7.40 (m, 1H), 7.33–7.26 (m, 1H), 7.19–7.14 (m, 1H), 7.09 (dd, *J* = 8.2, 4.6 Hz, 1H), 7.04–6.96 (m, 2H), 6.92 (dd, *J* = 9.0, 2.9 Hz, 2H), 4.62 (dd, *J* = 14.0, 3.9 Hz, 1H), 4.39 (dd, *J* = 14.0, 8.8 Hz, 1H), 3.69 (s, 3H), 3.34 (s, 3H), 3.30 (s, 3H), 3.13 (s, 3H), 1.30 (s, 9H) ppm; ^13^C NMR (101 MHz, CDCl_3_): *δ* 161.1, 159.2, 158.9, 158.6, 143.1 (d, *J* = 8.5 Hz), 137.7, 136.7 (d, *J* = 7.2 Hz), 136.4, 135.3, 134.9, 133.9, 132.9, 132.2 (d, *J* = 13.9 Hz), 131.7 (d, *J* = 10.4 Hz), 131.2 (d, *J* = 2.9 Hz), 129.8 (d, *J* = 27.2 Hz), 126.6 (d, *J* = 13.7 Hz), 125.0, 117.2 (d, *J* = 21.7 Hz), 110.3 (d, *J* = 88.9 Hz), 105.0 (d, *J* = 75.8 Hz), 59.6, 55.9, 55.4 (d, *J* = 10.5 Hz), 54.8, 47.9 (d, *J* = 5.5 Hz), 24.31 ppm; ^31^P NMR (162 MHz, CDCl_3_) *δ* 33.9 ppm. HRMS (ESI): *m*/*z* calcd. for C_35_H_38_NO_7_PS [M + Na]^+^ 670.1999, found 670.2007.

*N-(2-(Bis(2,7-diisopropoxynaphthalen-1-yl)phosphoryl)benzyl)-2-methylpropane-2-sulfonamide* (**5b**). The general procedure was employed, and the product was purified by silica gel (200–300 mesh) column chromatography using petroleum ether/ethyl acetate (2:1 *v*/*v*) as eluent to obtain **5b** as a white solid (39.5 mg, 52% yield). mp: 112–114 °C; ${\left[\alpha \right]}_{D}^{25}$ = −2.1 (*c* = 0.5, CH_2_Cl_2_). ^1^H NMR (400 MHz, CDCl_3_): *δ* 8.74 (s, 1H), 8.08 (s, 1H), 7.81 (t, *J* = 10.2 Hz, 2H), 7.65–7.54 (m, 3H), 7.42–7.31 (m, 4H), 7.15–7.09 (m, 1H), 6.98–6.79 (m, 4H), 4.63 (dd, *J* = 13.5, 4.2 Hz, 1H), 4.57–4.51 (m, 1H), 4.49–4.41 (m, 2H), 4.36–4.29 (m, 1H), 3.78–3.70 (m, 1H), 1.37 (t, *J* = 5.0 Hz, 3H), 1.31 (s, 9H), 1.12 (t, *J* = 4.5 Hz, 3H), 1.05 (t, *J* = 4.5 Hz, 3H), 0.97 (t, *J* = 5.0 Hz, 3H), 0.83 (t, *J* = 4.6 Hz, 3H), 0.70 (t, *J* = 5.0 Hz, 3H), 0.58 (t, *J* = 5.1 Hz, 3H), 0.23 (t, *J* = 5.1 Hz, 3H) ppm; ^13^C NMR (101 MHz, CDCl_3_): *δ* 158.8, 157.0, 156.7, 156.3, 143.0 (d, *J* = 8.1 Hz), 138.0 (d, *J* = 6.2 Hz), 137.1, 137.0 (d, *J* = 6.6 Hz), 136.0, 134.3, 133.2, 132.8 (d, *J* = 13.9 Hz), 132.0 (d, *J* = 10.4 Hz), 130.9, 129.4 (d, *J* = 22.0 Hz), 128.5, 127.6, 127.45, 126.5 (d, *J* = 13.9 Hz), 124.3 (d, *J* = 9.5 Hz), 124.0 (d, *J* = 9.6 Hz), 117.6, 117.4, 115.6, 114.5, 111.9, 110.8, 110.0 (d, *J* = 7.5 Hz), 109.6 (d, *J* = 8.0 Hz), 107.1, 106.7 (d, *J* = 6.8 Hz), 69.6, 69.2 (d, *J* = 5.3 Hz), 68.6, 59.4, 48.6 (d, *J* = 5.5 Hz), 48.2, 39.9 (q, *J* = 21.3 Hz), 24.2, 21.6 (d, *J* = 10.7 Hz), 21.4, 21.1, 20.9 (d, *J* = 4.7 Hz), 20.6, 19.8 ppm; ^31^P NMR (162 MHz, CDCl_3_) *δ* 33.3 ppm. HRMS (ESI): *m*/*z* calcd. for C_39_H_36_N_2_O_2_S [M + Na]^+^ 619.2390, found 619.2397.

*N-(2-(Bis(2,7-diisobutoxynaphthalen-1-yl)phosphoryl)benzyl)-2-methylpropane-2-sulfonamide* (**5c**). The general procedure was employed, and the product was purified by silica gel (200–300 mesh) column chromatography using petroleum ether/ethyl acetate (2:1 *v*/*v*) as eluent to obtain **5c** as a white solid (30.2 mg, 37% yield). mp: 133–135 °C; ${\left[\alpha \right]}_{D}^{25}$ = −0.45 (*c* = 0.3, CH_2_Cl_2_). ^1^H NMR (400 MHz, CDCl_3_): *δ* 8.68 (s, 1H), 8.15 (s, 1H), 7.82 (t, *J* = 8.7 Hz, 2H), 7.65 (dd, *J* = 9.0, 1.7 Hz, 1H), 7.60–7.56 (m, 2H), 7.41–7.31 (m, 2H), 7.15–7.04 (m, 2H), 7.02 (dd, *J* = 8.9, 2.4 Hz, 1H), 6.97 (dd, *J* = 9.0, 5.2 Hz, 1H), 6.92–6.87 (m, 2H), 4.55–4.34 (m, 2H), 3.68 (dd, *J* = 9.3, 6.4 Hz, 1H), 3.55–3.46 (m, 2H), 3.39–3.26 (m, 3H), 3.01 (dd, *J* = 9.7, 6.7 Hz, 1H), 2.72 (dd, *J* = 9.1, 6.5 Hz, 1H), 2.02–1.93 (m, 1H), 1.85–1.75 (m, 2H), 1.30 (s, 9H), 1.24–1.17 (m, 1H), 0.96 (dd, *J* = 6.7, 4.7 Hz, 6H), 0.80–0.75 (m, 6H), 0.68 (d, *J* = 6.7 Hz, 3H), 0.50 (dd, *J* = 14.7, 6.6 Hz, 6H), 0.37 (d, *J* = 6.6 Hz, 3H) ppm; ^13^C NMR (101 MHz, CDCl_3_): *δ* 160.7, 159.2, 158.6, 158.1, 143.3, 137.7, 136.9 (d, *J* = 4.3 Hz), 135.8, 134.6, 133.7, 132.9 (d, *J* = 13.5 Hz), 132.2 (d, *J* = 10.2 Hz), 131.1, 129.6 (d, *J* = 27.8 Hz), 126.4 (d, *J* = 13.4 Hz), 124.8 (t, *J* = 9.6 Hz), 117.6, 117.2, 114.5, 111.2, 110.5 (d, *J* = 7.6 Hz), 106.2, 105.4, 76.0, 75.9, 74.1, 73.7, 59.6, 48.1, 28.2, 28.0, 27.8, 27.7, 24.4, 19.3 (t, *J* = 4.2 Hz), 19.1 (d, *J* = 2.1 Hz), 19.0, 18.7 ppm; ^31^P NMR (162 MHz, CDCl_3_) *δ* 34.8 ppm. HRMS (ESI): *m*/*z* calcd. for C_47_H_62_NO_7_PS [M + Na]^+^ 838.3877, found 838.3854.

*N-(2-(Bis(2,7-bis(cyclopentyloxy)naphthalen-1-yl)phosphoryl)benzyl)-2-methylpropane-2-sulfonamide* (**5d**). The general procedure was employed, and the product was purified by silica gel (200–300 mesh) column chromatography using petroleum ether/ethyl acetate (2:1 *v*/*v*) as eluent to obtain **5d** as a white solid (23.3 mg, 27% yield). mp: 165–167 °C; ${\left[\alpha \right]}_{D}^{25}$ = −0.6 (*c* = 0.3, CH_2_Cl_2_). ^1^H NMR (400 MHz, CDCl_3_): *δ* 8.56 (s, 1H), 8.12 (s, 1H), 7.80 (dd, *J* = 12.4, 9.0 Hz, 2H), 7.62 (d, *J* = 7.3 Hz, 1H), 7.59–7.54 (m, 2H), 7.39–7.30 (m, 2H), 7.21 (dd, *J* = 8.8, 4.2 Hz, 1H), 7.12–7.06 (m, 1H), 6.96–6.83 (m, 4H), 4.75–4.69 (m, 1H), 4.60–4.51 (m, 3H), 4.34 (dd, *J* = 13.6, 8.7 Hz, 1H), 3.96–3.91 (m, 1H), 2.03–1.96 (m, 1H), 1.85–1.46 (m, 19H), 1.32–1.28 (m, 9H + 4H), 1.20–0.92 (m, 6H), 0.80–0.67 (m, 2H) ppm; ^13^C NMR (101 MHz, CDCl_3_): *δ* 159.5, 158.0 (d, *J* = 3.0 Hz), 157.2, 156.8, 143.1 (d, *J* = 8.1 Hz), 137.8 (d, *J* = 6.3 Hz), 134.2, 133.3, 133.1, 132.2 (d, *J* = 10.3 Hz), 130.9, 129.5 (d, *J* = 16.8 Hz), 126.3 (d, *J* = 13.6 Hz), 124.3 (d, *J* = 16.9 Hz), 124.2 (d, *J* = 16.9 Hz), 117.8, 117.4, 114.1, 110.6 (dd, *J* = 15.2, 7.6 Hz), 107.7, 106.8 (d, *J* = 6.7 Hz), 79.8, 79.6, 79.4, 78.9, 59.7, 48.5, 32.8 (d, *J* = 19.0 Hz), 32.5, 32.2 (d, *J* = 2.4 Hz), 32.0, 31.7 (d, *J* = 4.6 Hz), 24.4, 24.3, 24.0 (d, *J* = 3.7 Hz), 23.9 (d, *J* = 3.5 Hz), 23.8 ppm; ^31^P NMR (162 MHz, CDCl_3_) *δ* 32.8 ppm. HRMS (ESI): *m*/*z* calcd. for C_51_H_62_NO_7_PS [M + Na]^+^ 886.3877, found 886.3839.

## 4. Summary

We have successfully designed and synthesized chiral targets that exclusively feature the element of turbo chirality. The chirality is effectively controlled using a sulfonimine auxiliary through catalytic P–C(sp^2^) bond formation, followed by oxidation. The resulting configurations and conformations were unambiguously confirmed via X-ray diffraction analysis. The three propeller-like structures of the turbo frameworks are covalently linked to the phosphorus center of the P=O bond, displaying either a clockwise (PPP) or counterclockwise (MMM) molecular arrangement. Notably, for each of the three propellers, the bulkier part of the aromatic planes is directed toward the oxygen of the P=O bond, rather than away from it. Computational studies were performed to examine the relative energies of the rotational barriers along the P=O axis, as well as the transition pathway between the two enantiomers, aligning with our theoretical expectations. This work is poised to have a significant impact across chemical, biomedical, and material sciences in the future.

## Data Availability

All data are available in the manuscript or the Supplementary Materials.

## Supplementary Materials

The following supporting information can be downloaded at https://www.mdpi.com/article/10.3390/molecules30030603/s1, General synthesis of turbo chiral targets, precursors and analytical data, ^1^H NMR spectra, ^13^C NMR spectra, and X-ray diffraction analysis data.
