# Supplementary material for: Enantiopure Turbo Chirality Targets in Tri-Propeller Blades: Design, Asymmetric Synthesis, and Computational Analysis
Source: Molecules. 2025 Jan 29;30(3):603. doi: 10.3390/molecules30030603 (PMC11819669; doi:10.3390/molecules30030603)
Supplement: Supplementary file 1 [file molecules-30-00603-s001.zip › molecules-3395828-supplementary.pdf]

Enantiopure Turbo Chirality in Tri-Propeller Targets: Design and Asymmetric Synthesis, and Computational Analysis

Yu Wang,[1,†] Ting Xu,[1,†] Ankit Pandey,[2] Shengzhou Jin,[1] Jasmine X. Yan,[2] Qingkai Yuan,[2] Sai Zhang,[3] Jia-Yin Wang,[3] Ruibin Liang,[*,2] and Guigen Li[*,1,2]

[1] *School of Chemistry and Chemical Engineering, Nanjing University, Nanjing, 210093, China.*

[2]*Department of Chemistry and Biochemistry, Texas Tech University, Lubbock, TX 79409-1061, USA.*

[3] *School of Pharmacy, Continuous Flow Engineering Laboratory of National Petroleum and Chemical Industry, Changzhou University, Changzhou, 213164, China.*

[*]Correspondence should be addressed to Ruibin Liang and Guigen Li: rliang@ttu.edu *(RL)* guigen.li@ttu.edu, guigenli@nju.edu.cn *(GL)*

[†]These authors contributed equally to this work.

Supporting Information

Contents

1. Copies of ^{1}H, ^{13}C, ^{19}F and ^{31}P NMR spectra of compounds 1, 2, 4, 5 .... S1
2. X-ray Diffraction Data for 4a S48

1. Copies of ^{1}H, ^{13}C, ^{19}F and ^{31}P NMR spectra of compounds 1, 2, 4, 5

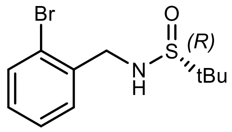

1a

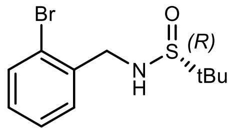

1a

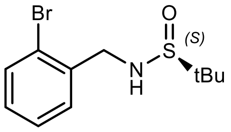

1a'

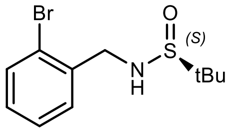

1a'

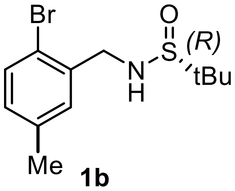

1b

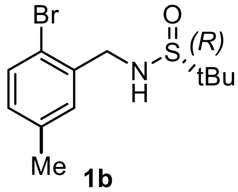

1b

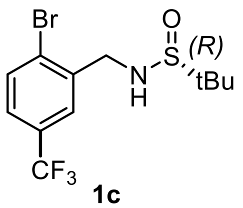

1c

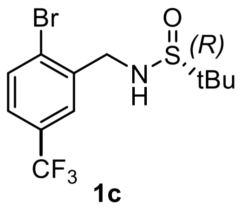

1c

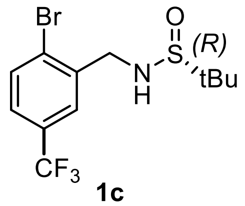

1c

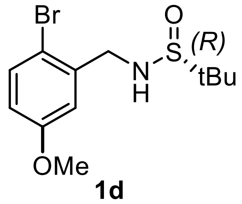

1d

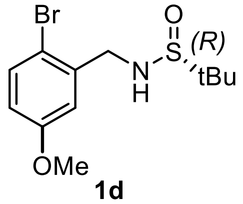

1d

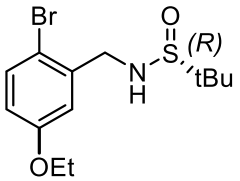

1e

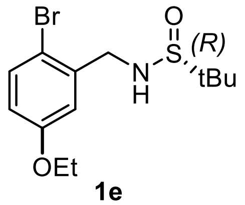

1e

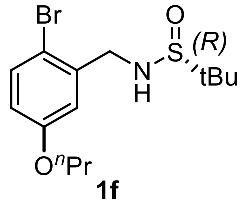

1f

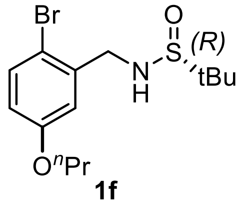

1f

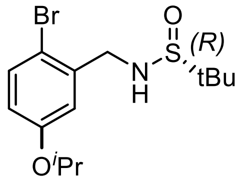

1g

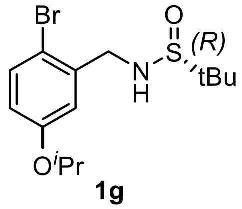

1g

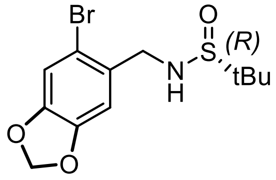

1h

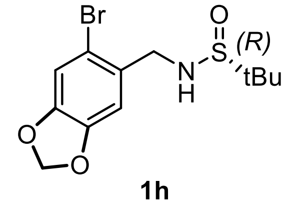

1h

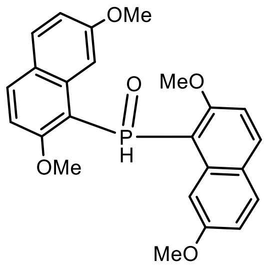

2a

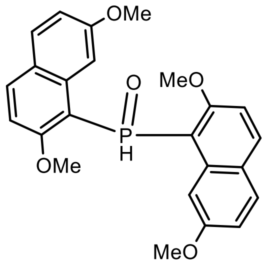

2a

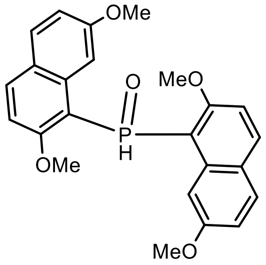

2a

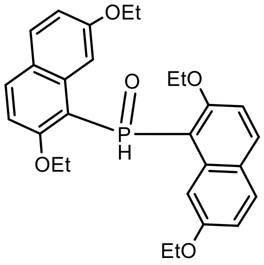

2b

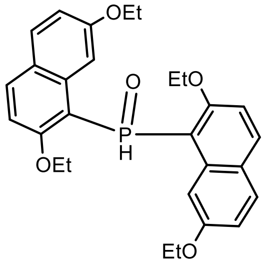

2b

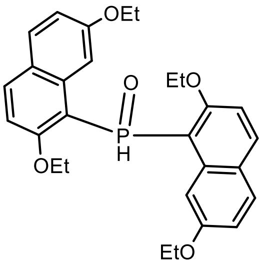

2b

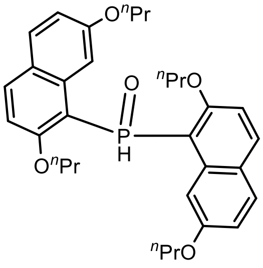

2c

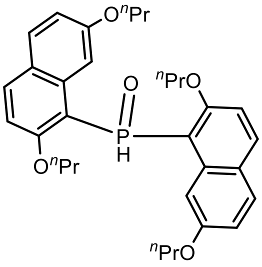

2c

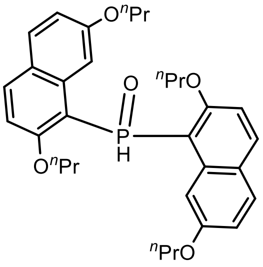

2c

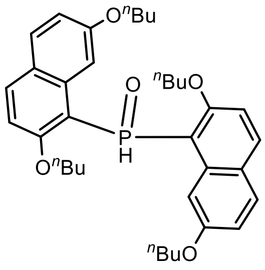

2d

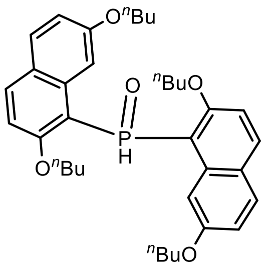

2d

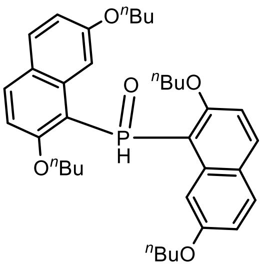

2d

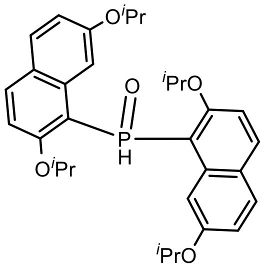

2e

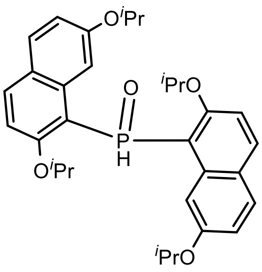

2e

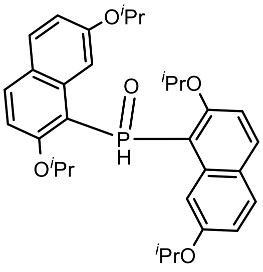

2e

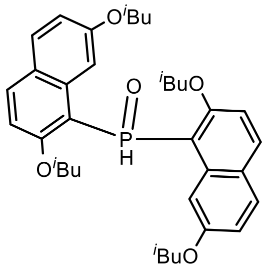

2f

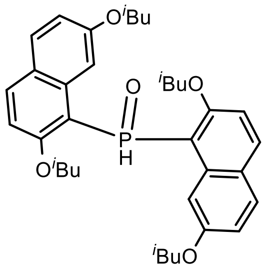

2f

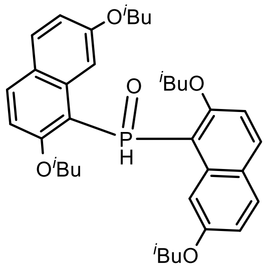

2f

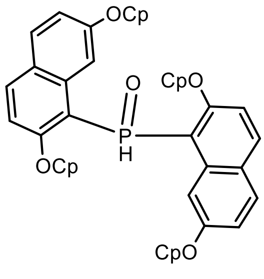

2g

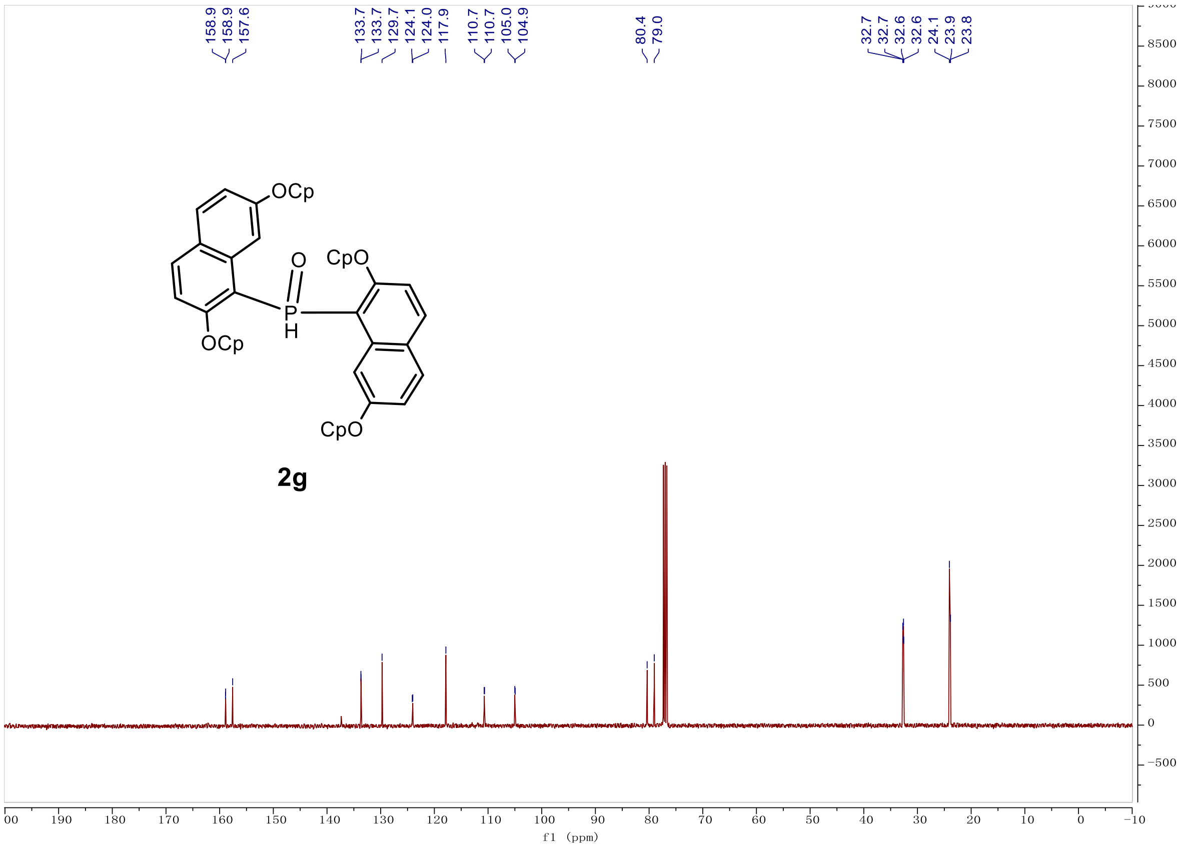
158.9
158.9
157.6
133.7
133.7
129.7
124.1
124.0
117.9
110.7
110.7
105.0
104.9
80.4
79.0
32.7
32.7
32.6
32.6
24.1
23.9
23.8
OCp
CpO
O
P
H
OCp
CpO
2g
f1 (ppm)

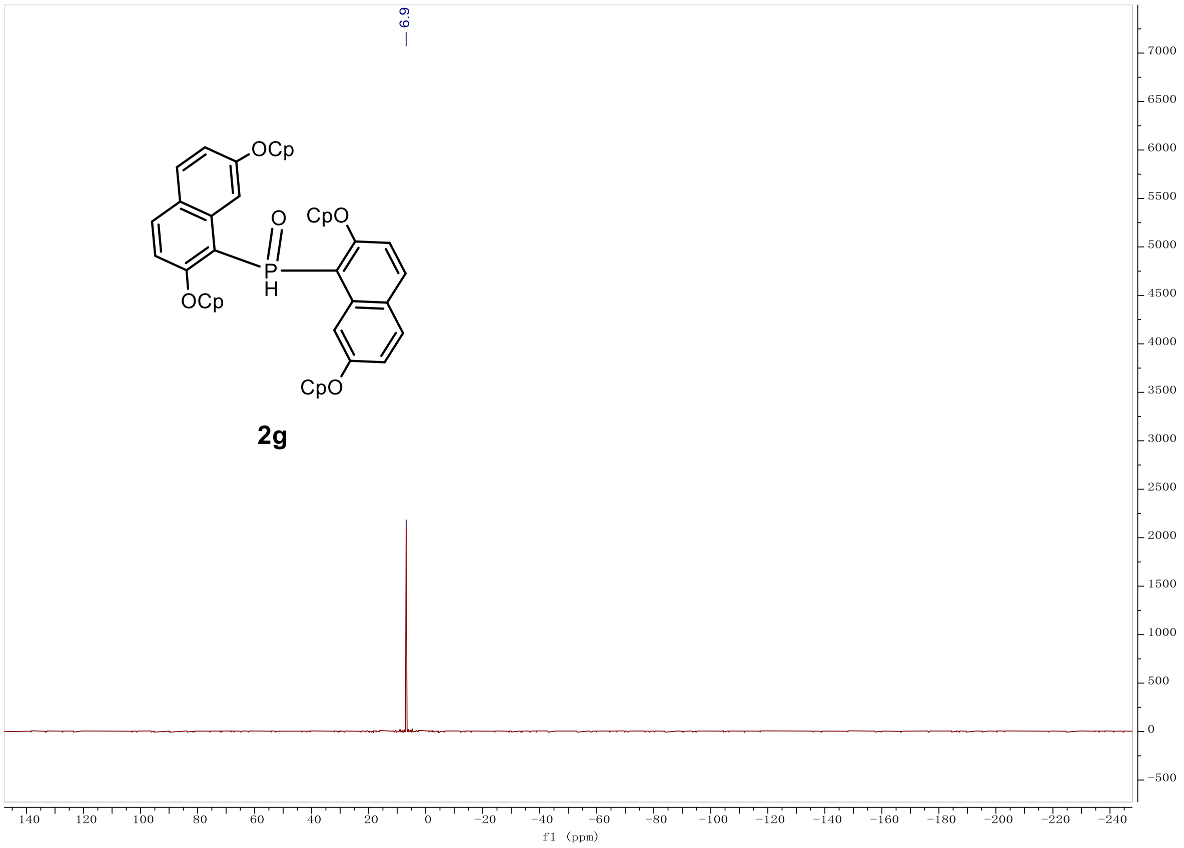
6.9
OCp
CpO
O
P
H
OCp
CpO
2g
f1 (ppm)

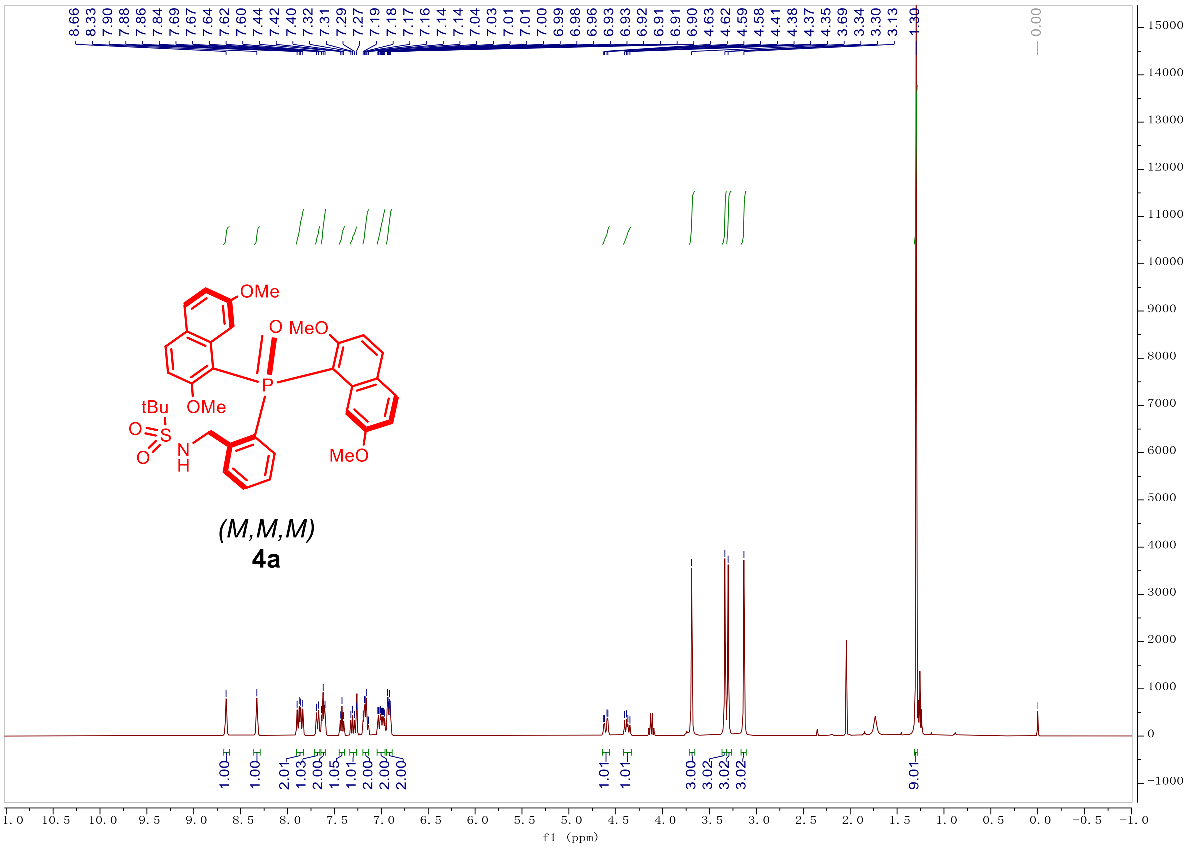
OMe
O MeO
P
tBu OMe
O=S
N
H
MeO
(M,M,M)
4a
f1 (ppm)

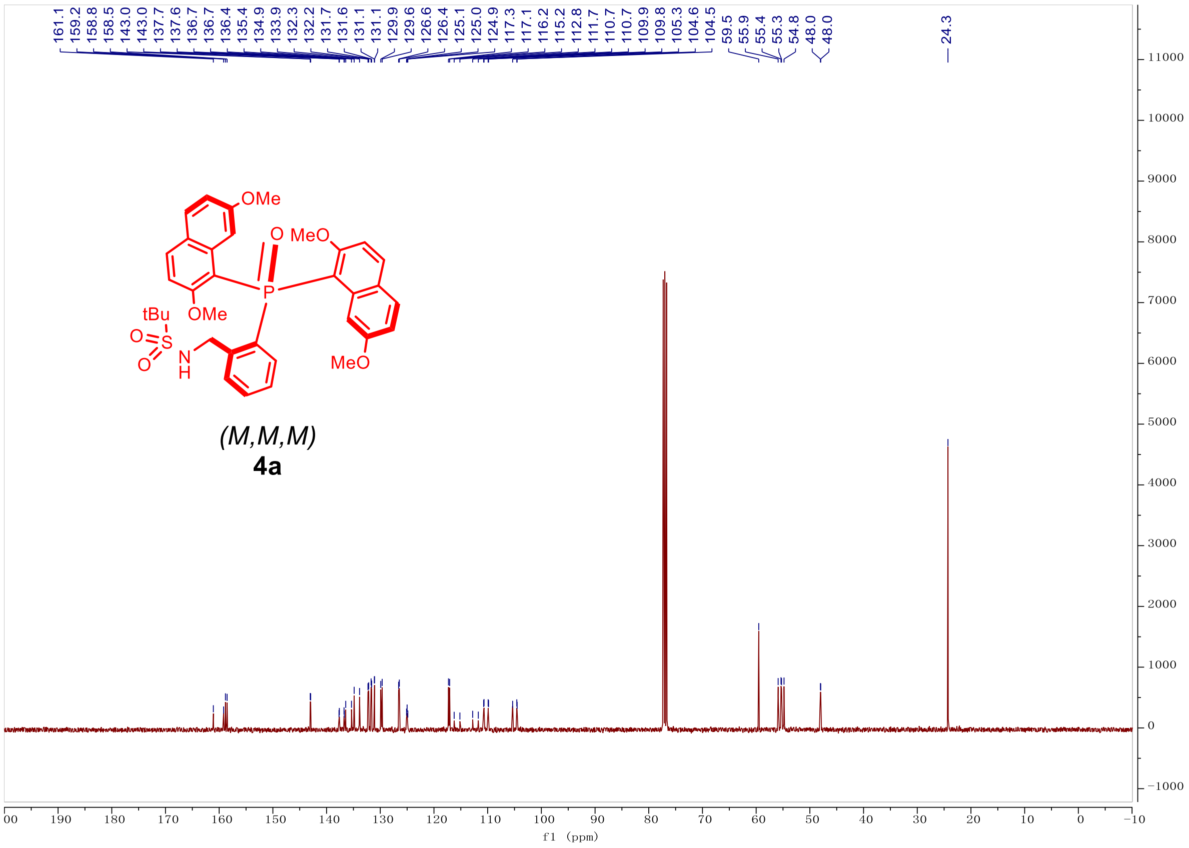
OMe
O MeO
P
tBu OMe
O=S
N
H
MeO
(M,M,M)
4a
f1 (ppm)

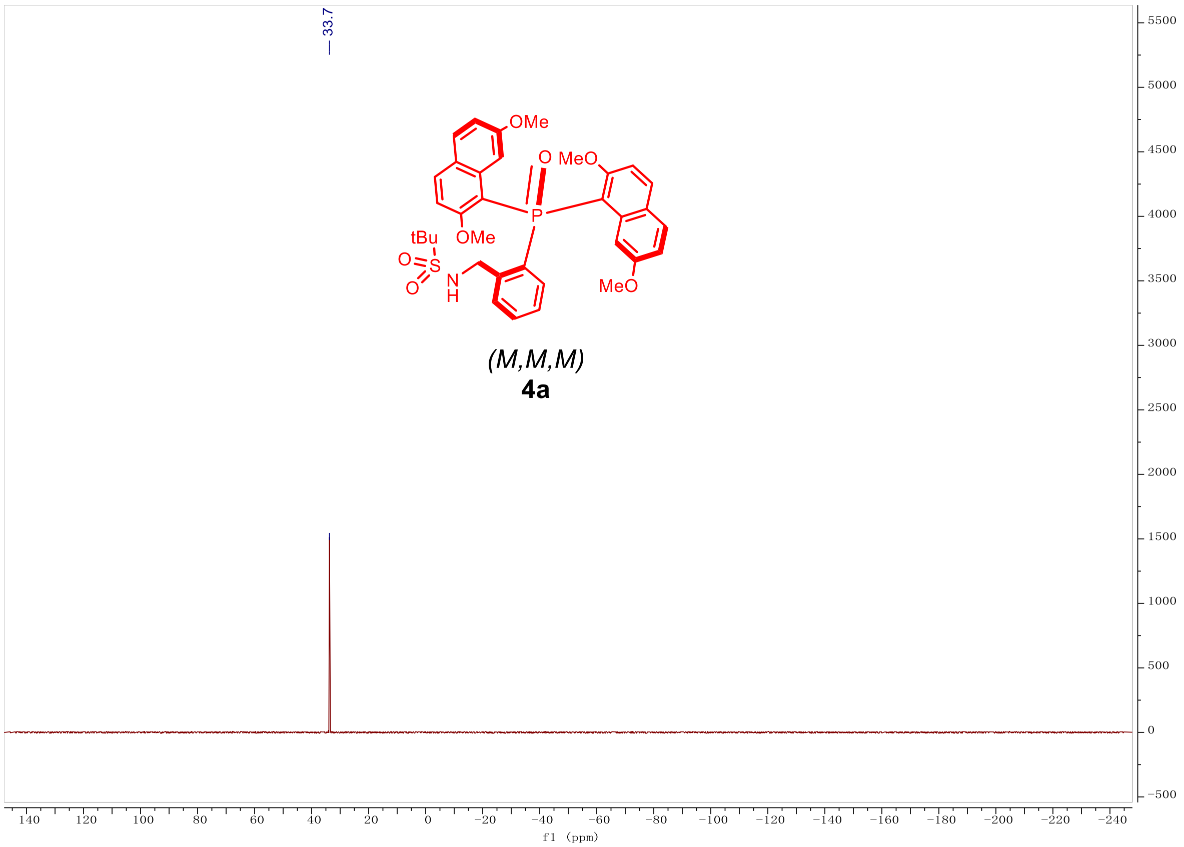
33.7
OMe
O
MeO
P
OMe
tBu
S
N
H
MeO
(M,M,M)
4a
f1 (ppm)

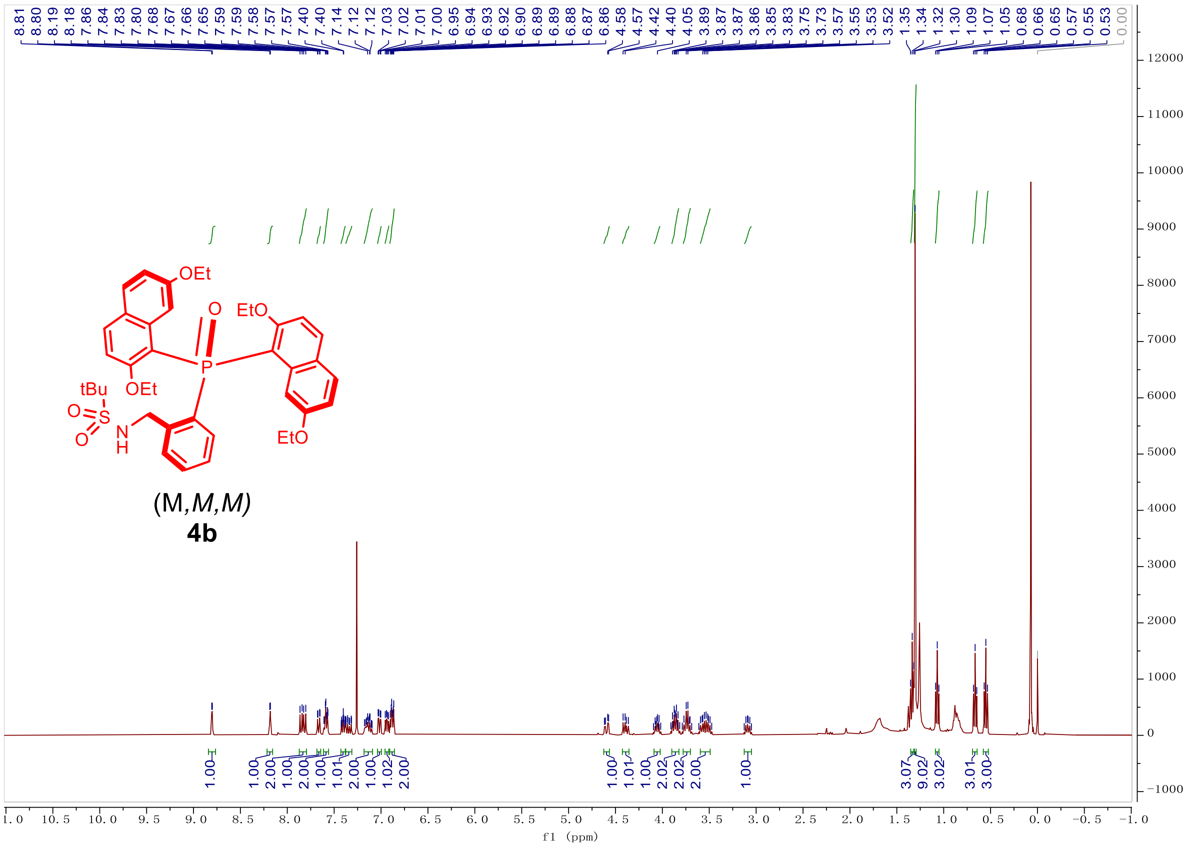
OEt
O
EtO
P
OEt
tBu
S
N
H
EtO
(M,M,M)
4b
f1 (ppm)

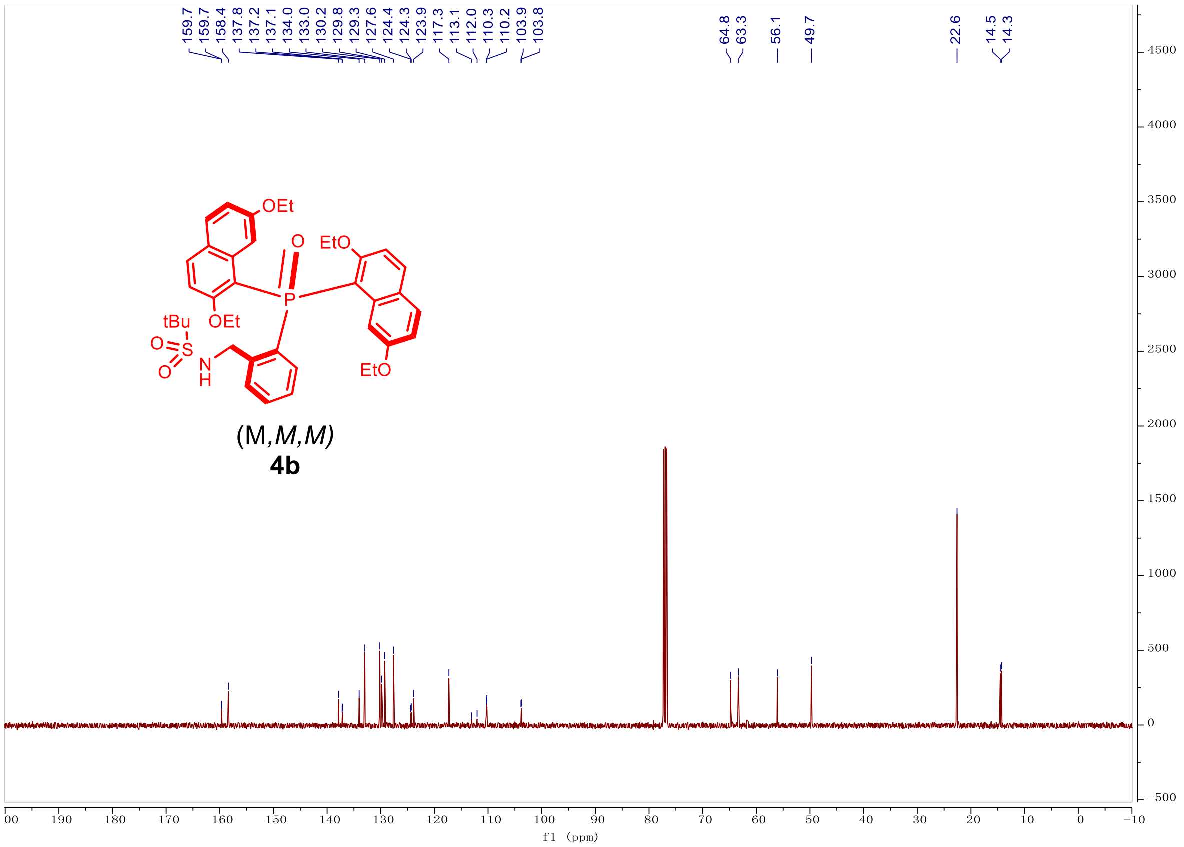
159.7
159.7
158.4
137.8
137.2
137.1
134.0
133.0
130.2
129.8
129.3
127.6
124.4
124.3
123.9
117.3
113.1
112.0
110.3
110.2
103.9
103.8
64.8
63.3
56.1
49.7
22.6
14.5
14.3
OEt
O
EtO
P
tBu
OEt
O
S
O
N
H
EtO
(M,*M*,*M*)
4b
f1 (ppm)

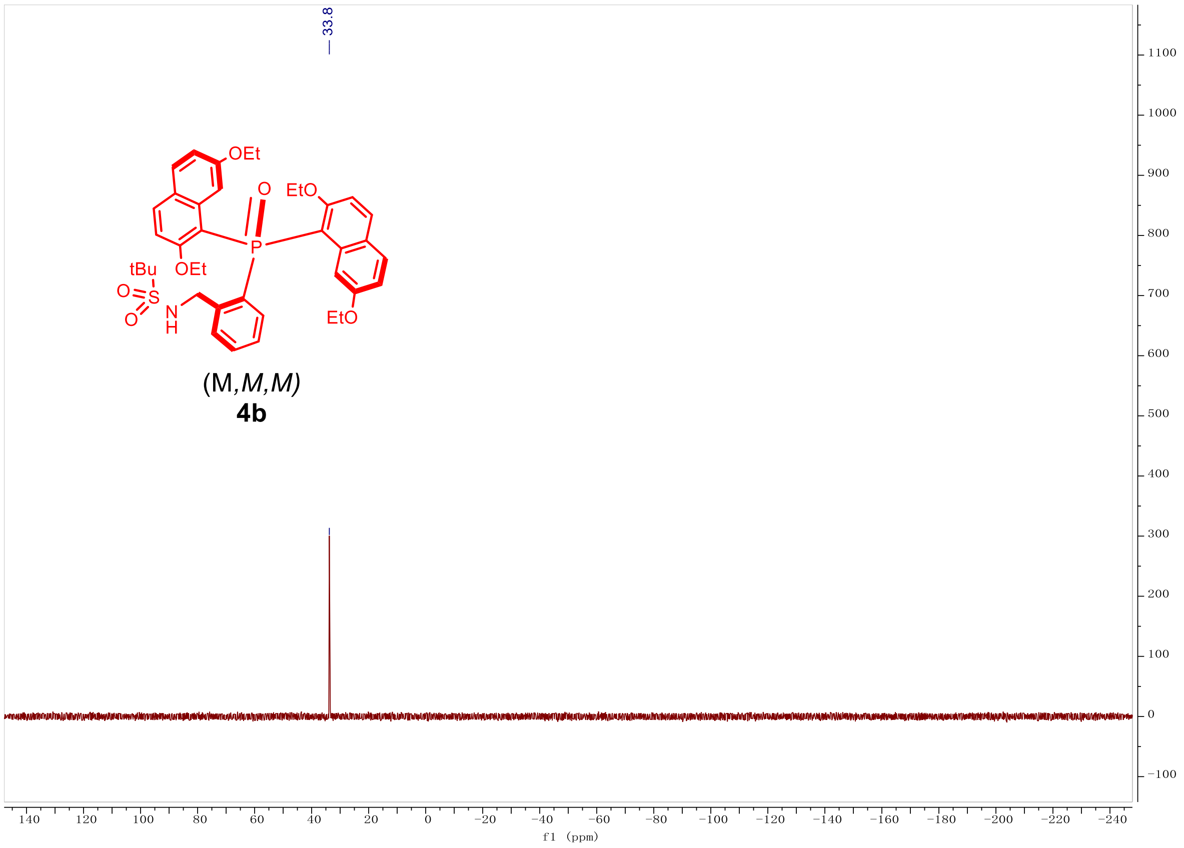
33.8
OEt
O
EtO
P
tBu
OEt
O
S
O
N
H
EtO
(M,*M*,*M*)
4b
f1 (ppm)

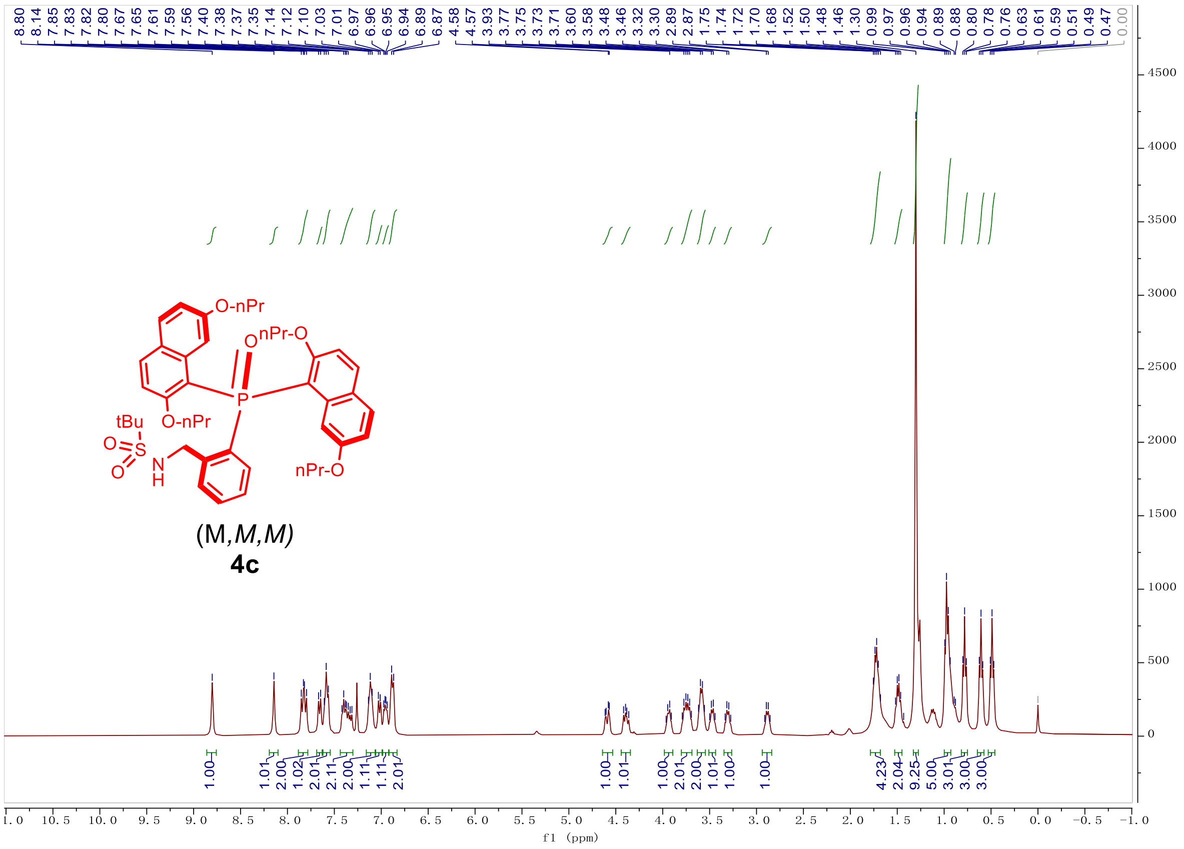

O-nPr
O
nPr-O
P
O-nPr
tBu
O
S
O
N
H
nPr-O
(M,M,M)
4c
f1 (ppm)

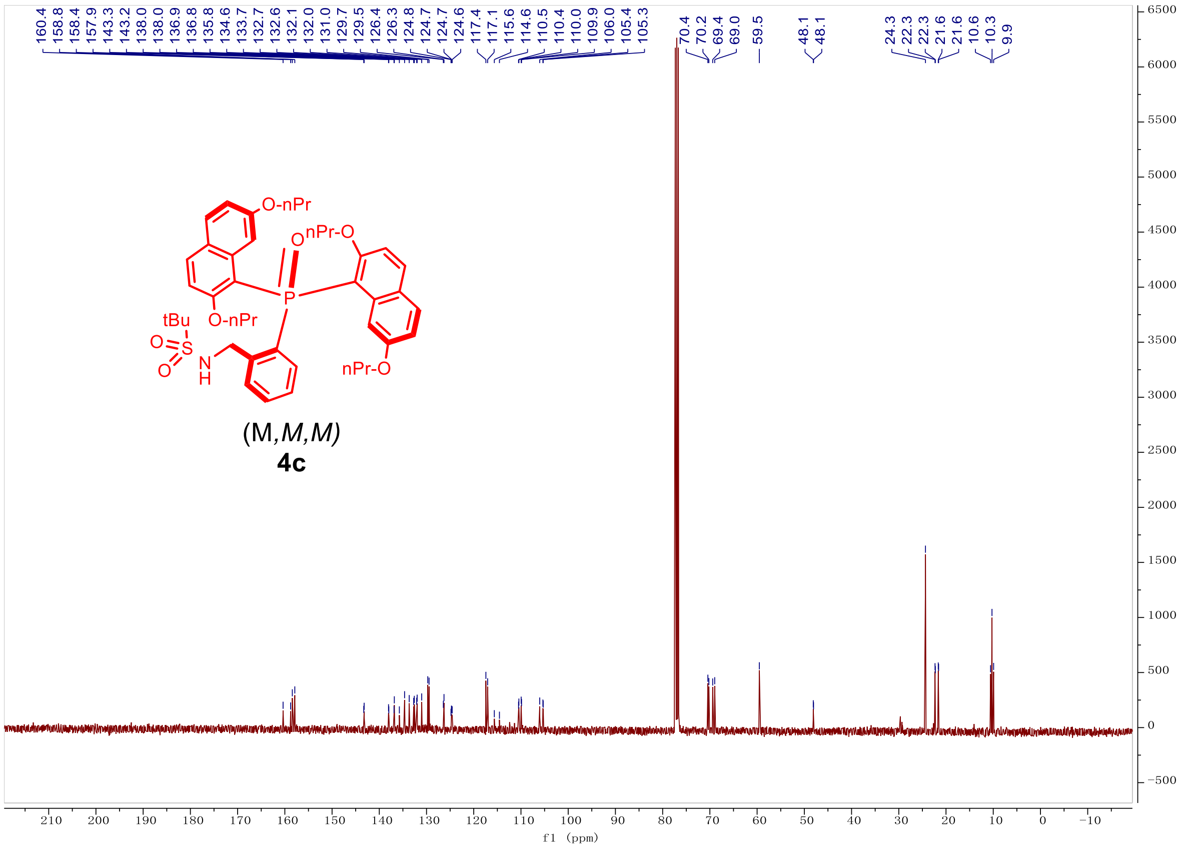

O-nPr
O
nPr-O
P
O-nPr
tBu
O
S
O
N
H
nPr-O
(M,M,M)
4c
f1 (ppm)

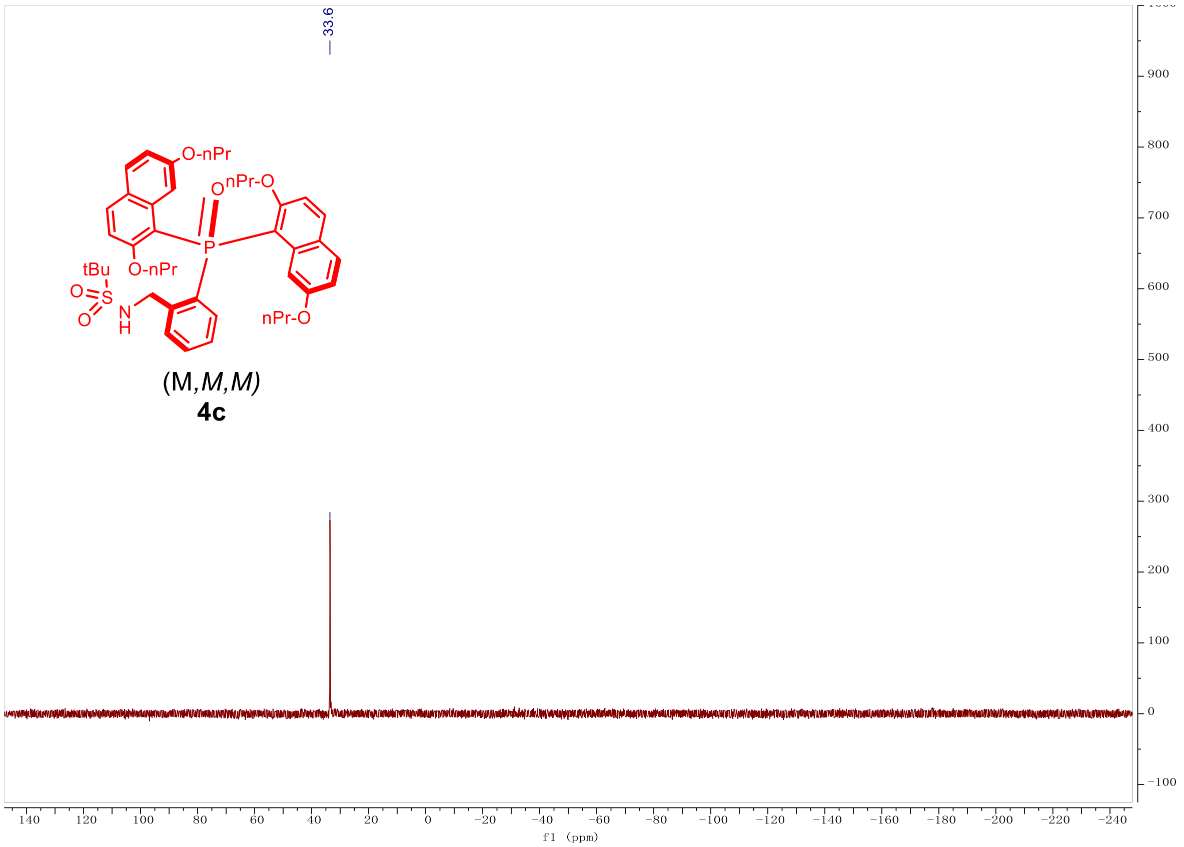

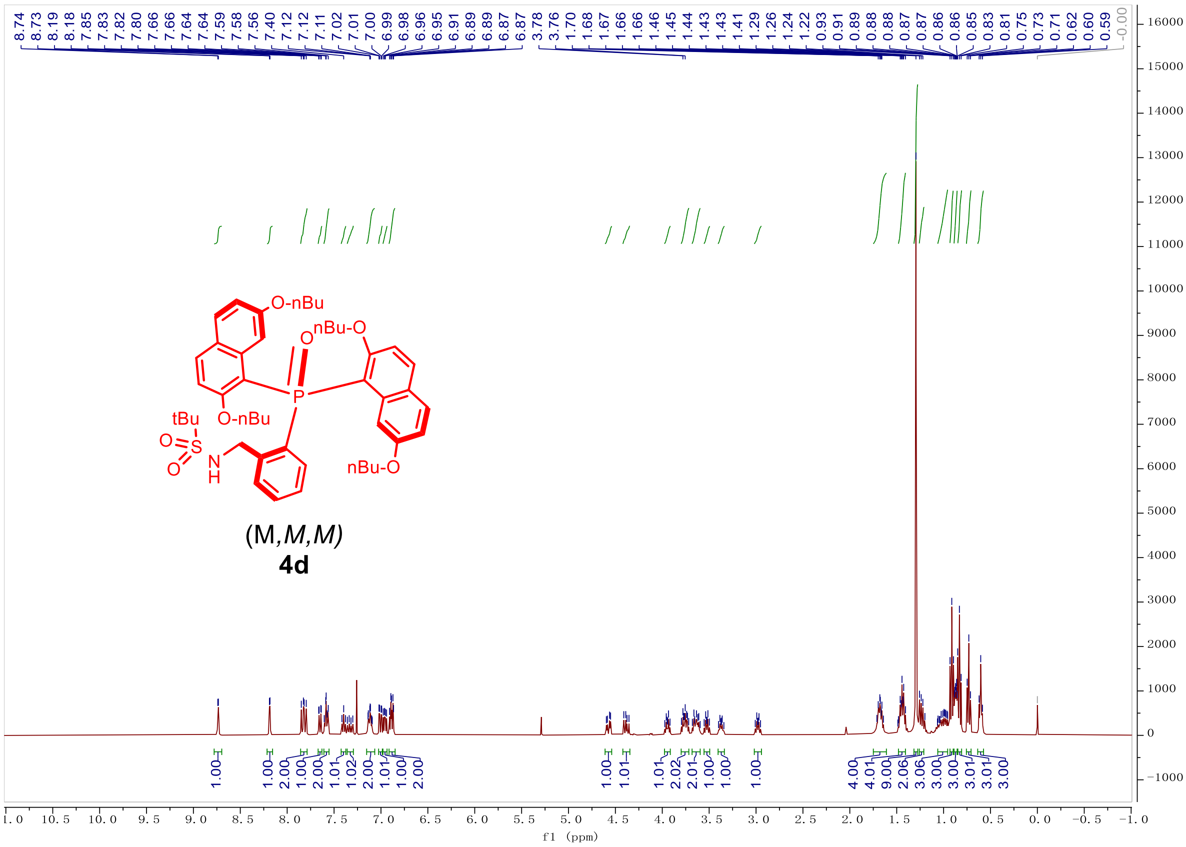

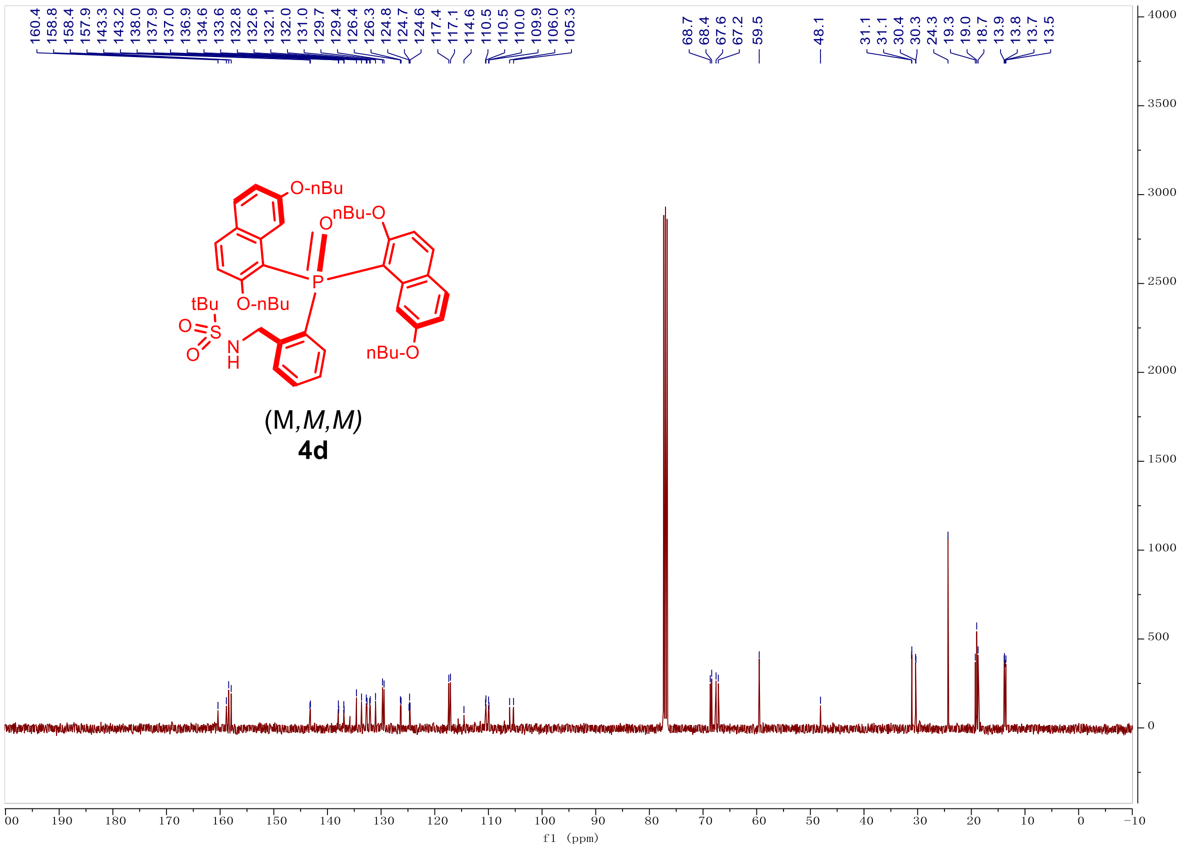
160.4
158.8
158.4
157.9
143.3
143.2
138.0
137.9
137.0
136.9
134.6
133.6
132.8
132.6
132.1
132.0
131.0
129.7
129.4
126.4
126.3
124.8
124.7
124.6
117.4
117.1
114.6
110.5
110.5
110.0
109.9
106.0
105.3
68.7
68.4
67.6
67.2
59.5
48.1
31.1
31.1
30.4
30.3
24.3
19.3
19.0
18.7
13.9
13.8
13.7
13.5
(M,*M*,*M*)
4d
f1 (ppm)

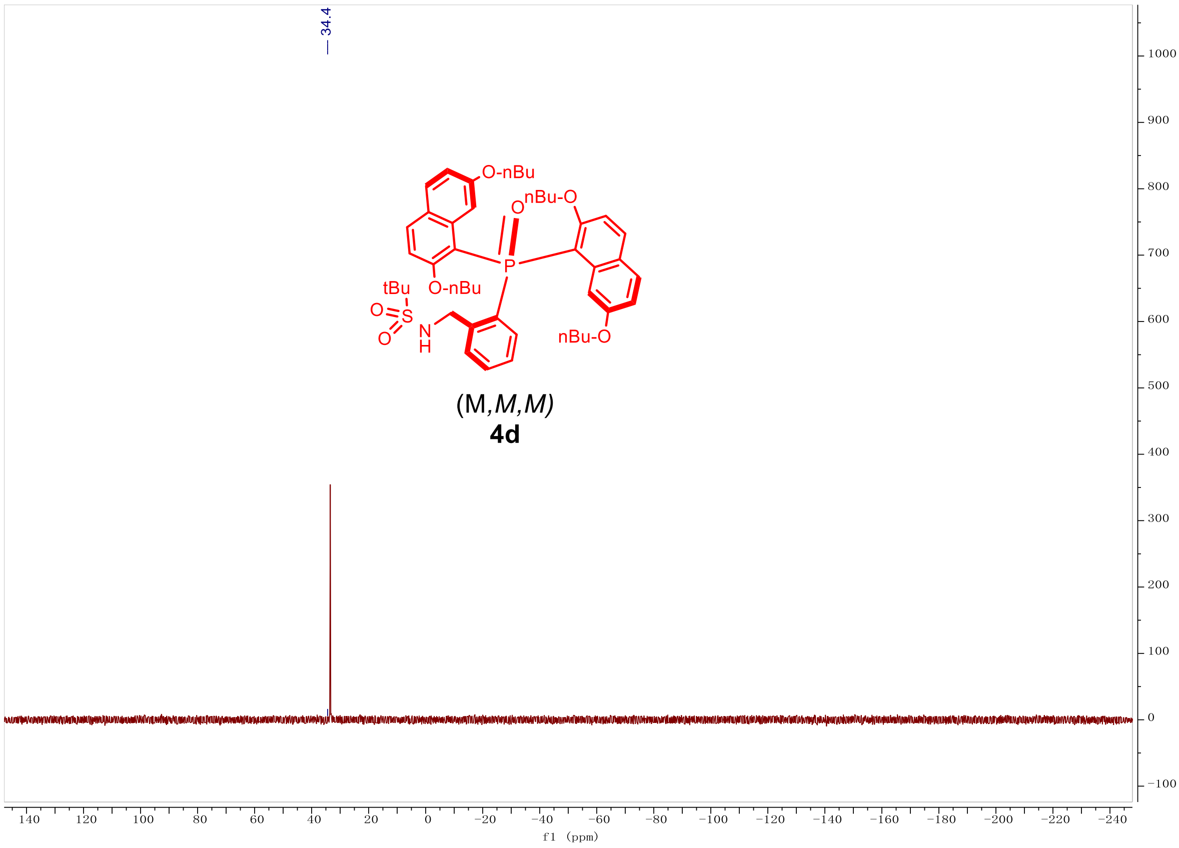
34.4
(M,*M*,*M*)
4d
f1 (ppm)

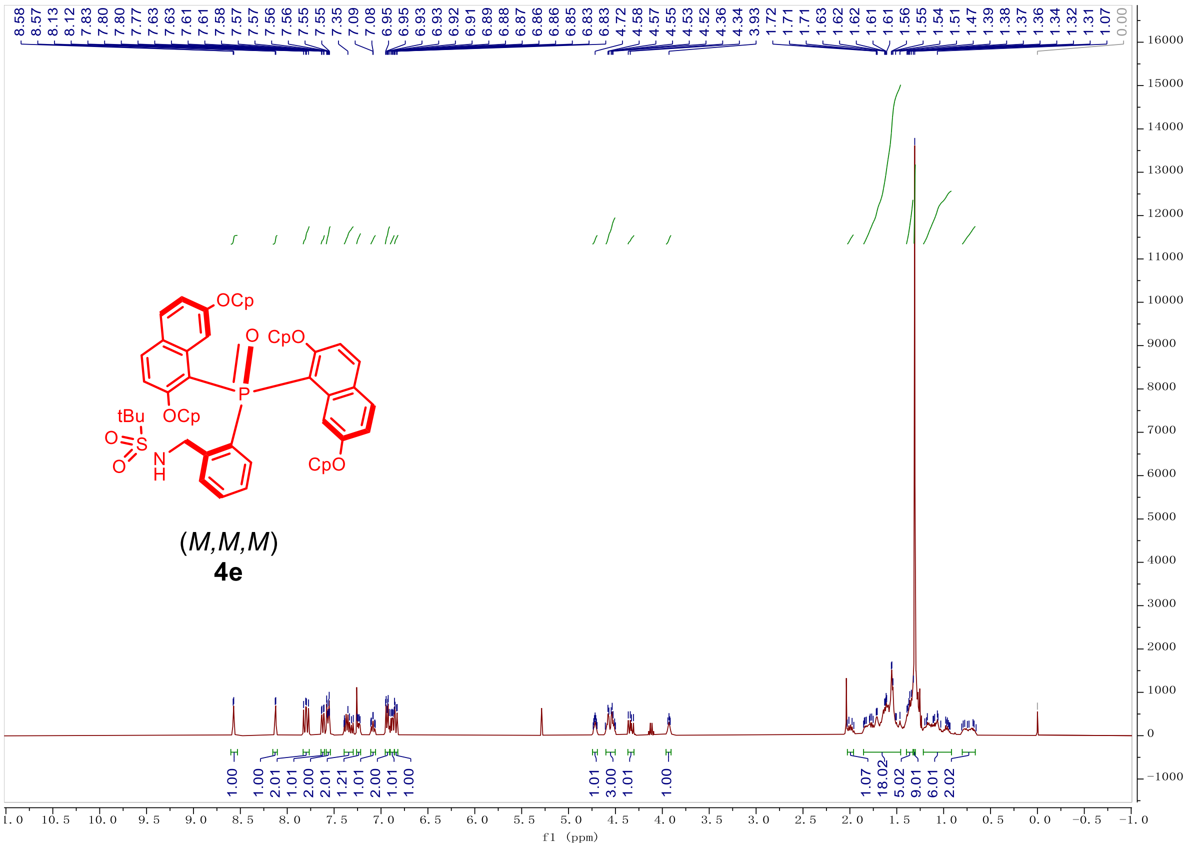
8.58 8.57 8.13 8.12 7.83 7.80 7.77 7.63 7.63 7.61 7.61 7.58 7.57 7.57 7.56 7.56 7.55 7.55 7.35 7.09 7.08 6.95 6.95 6.93 6.93 6.92 6.91 6.89 6.88 6.87 6.86 6.86 6.85 6.83 6.83 4.72 4.58 4.57 4.55 4.53 4.52 4.36 4.34 3.93 1.72 1.71 1.63 1.62 1.61 1.61 1.61 1.56 1.55 1.54 1.51 1.47 1.39 1.38 1.37 1.36 1.34 1.32 1.31 1.07 0.00
OCp
O
CpO
P
tBu
OCp
O=S
O
N
H
CpO
(M,M,M)
4e
1.00 1.00 2.01 1.01 2.00 2.01 1.21 1.01 2.00 1.01 1.00
1.01 3.00 1.01 1.00
1.07 18.02 5.02 9.01 6.01 2.02
f1 (ppm)

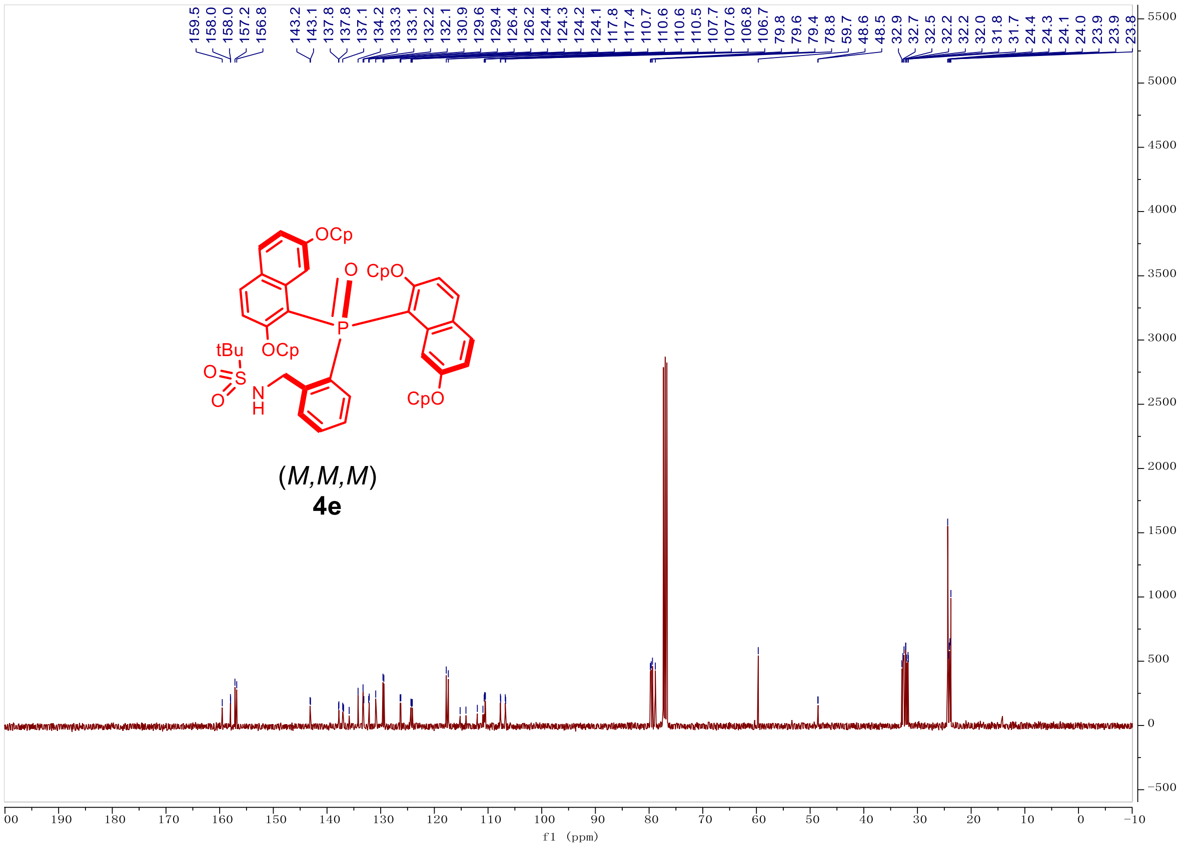
159.5 158.0 158.0 157.2 156.8 143.2 143.1 137.8 137.8 137.1 134.2 133.3 133.1 132.2 132.1 130.9 129.6 129.4 126.4 126.2 124.4 124.3 124.2 124.1 117.8 117.4 110.7 110.6 110.5 107.7 107.6 106.8 106.7 79.8 79.6 79.4 78.8 59.7 48.6 48.5 32.9 32.7 32.5 32.2 32.2 32.0 31.8 31.7 24.4 24.3 24.1 24.0 23.9 23.9 23.8
OCp
O
CpO
P
tBu
OCp
O=S
O
N
H
CpO
(M,M,M)
4e
f1 (ppm)

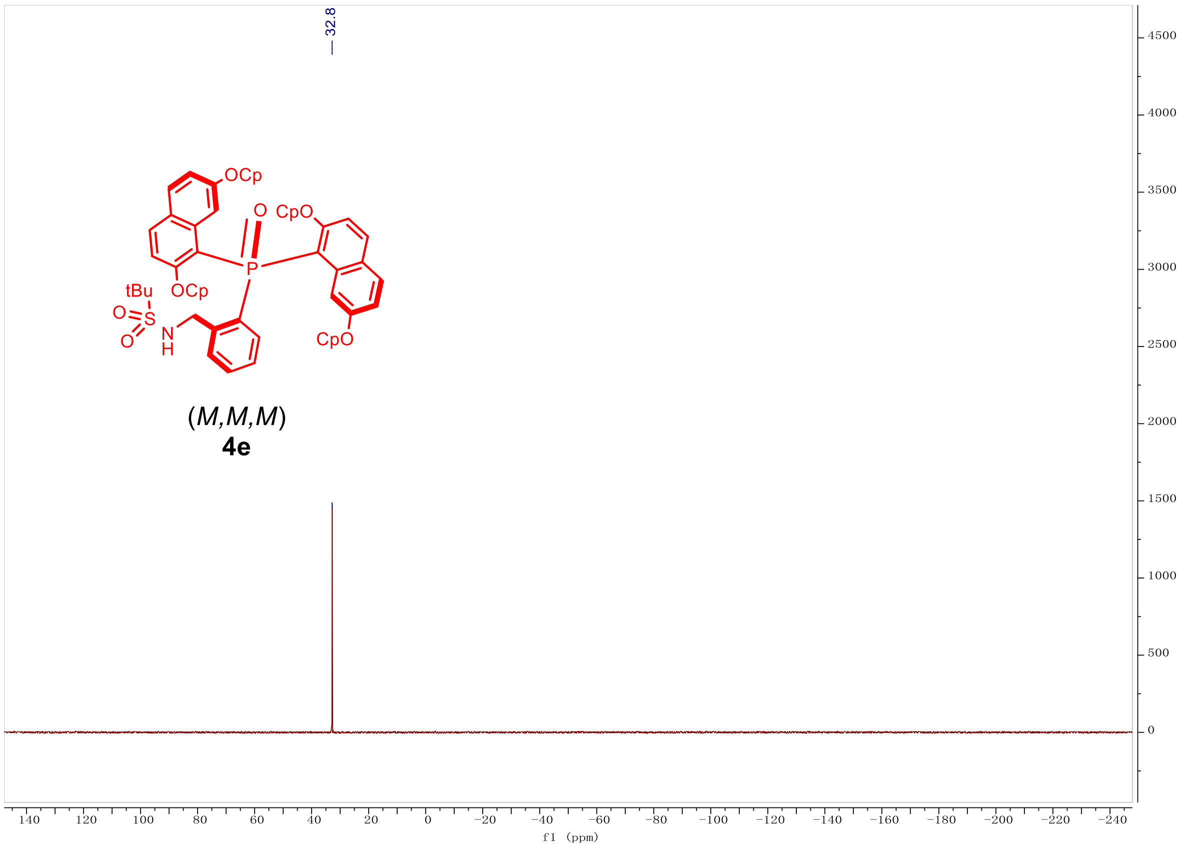
32.8
OCp
O
CpO
tBu
OCp
O=S
O
N
H
CpO
(M,M,M)
4e
f1 (ppm)

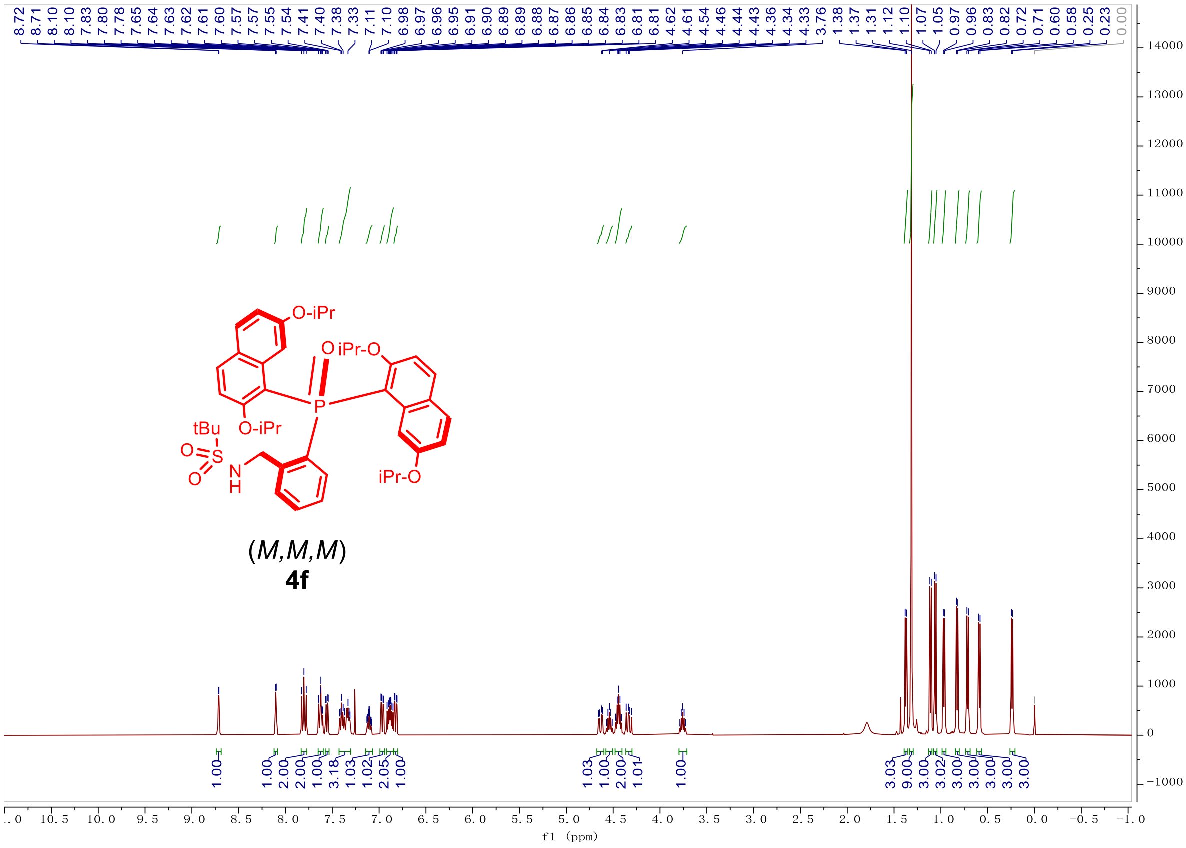
O-iPr
O
iPr-O
tBu
O-iPr
O=S
O
N
H
iPr-O
(M,M,M)
4f
f1 (ppm)

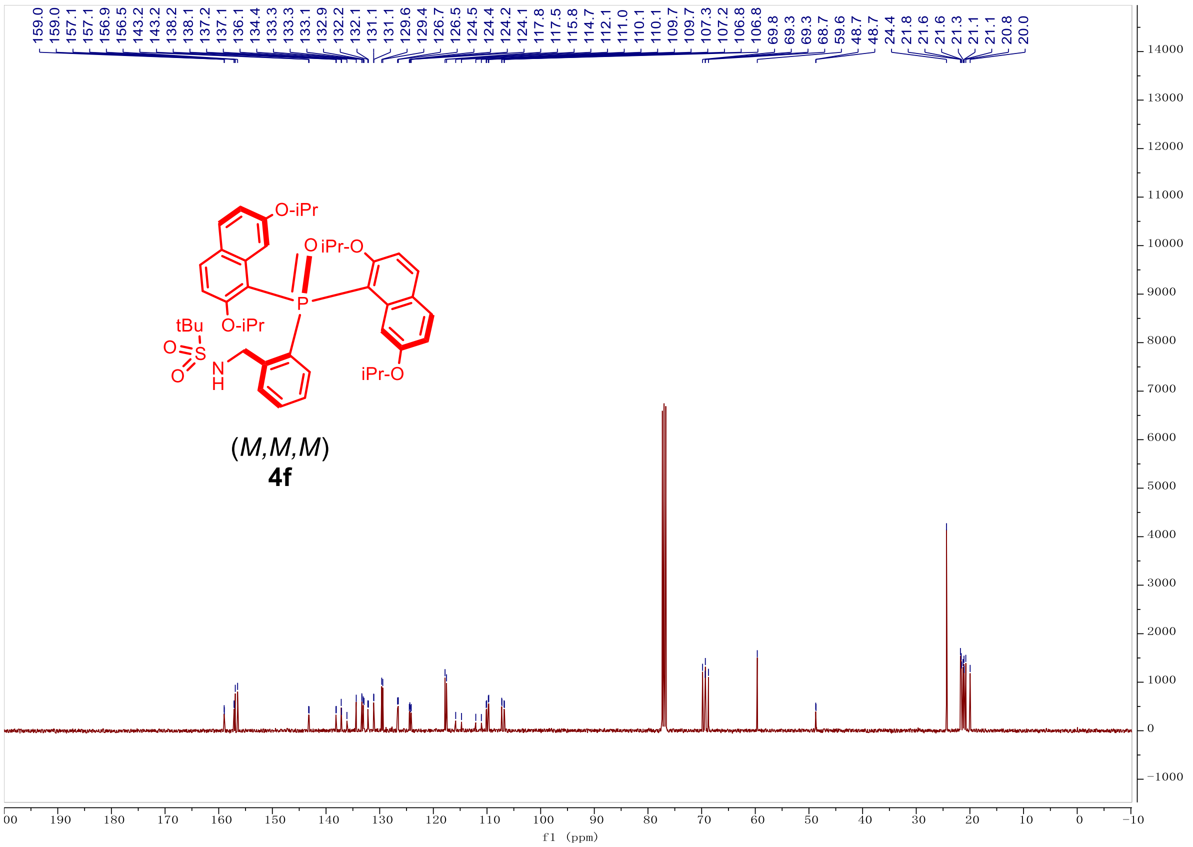
159.0
159.0
157.1
157.1
156.9
156.5
143.2
143.2
138.2
138.1
137.2
137.1
136.1
134.4
133.3
133.3
133.1
132.9
132.2
132.1
131.1
131.1
129.6
129.4
126.7
126.5
124.5
124.4
124.2
124.1
117.8
117.5
115.8
114.7
112.1
111.0
110.1
110.1
109.7
109.7
107.3
107.2
106.8
106.8
69.8
69.3
69.3
68.7
59.6
48.7
48.7
24.4
21.8
21.6
21.6
21.3
21.1
21.1
20.8
20.0
O-iPr
O
iPr-O
P
tBu
O-iPr
O=S
O
N
H
iPr-O
(M,M,M)
4f
f1 (ppm)

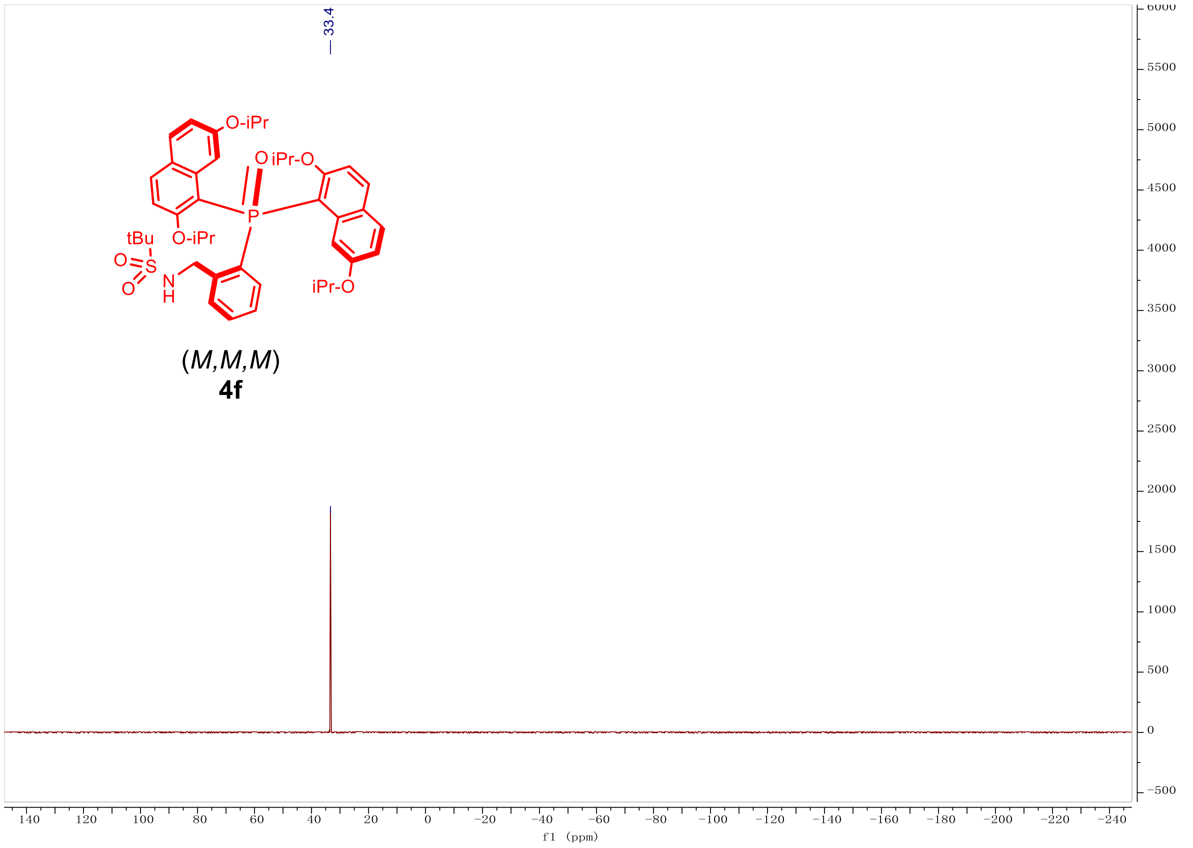
33.4
O-iPr
O
iPr-O
P
tBu
O-iPr
O=S
O
N
H
iPr-O
(M,M,M)
4f
f1 (ppm)

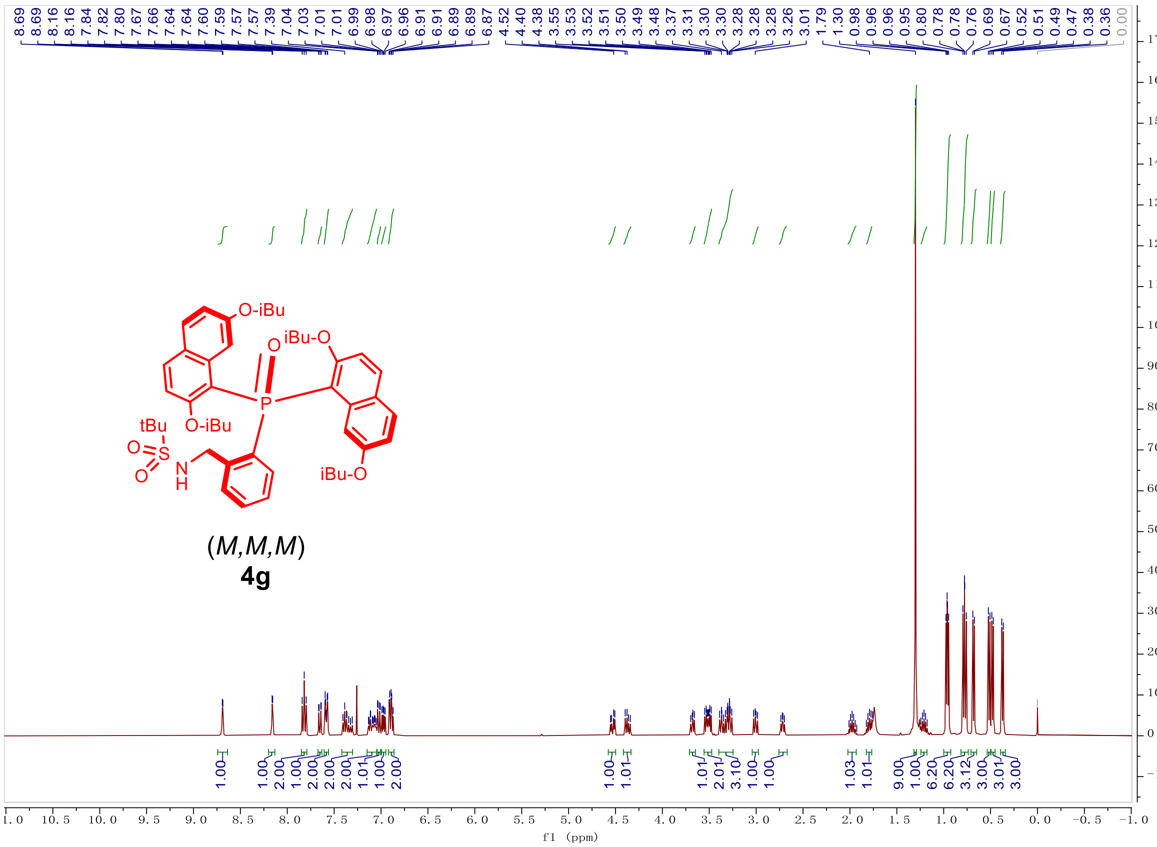
O-iBu
O
iBu-O
P
O-iBu
tBu
O=S
O
N
H
iBu-O
(M,M,M)
4g
f1 (ppm)

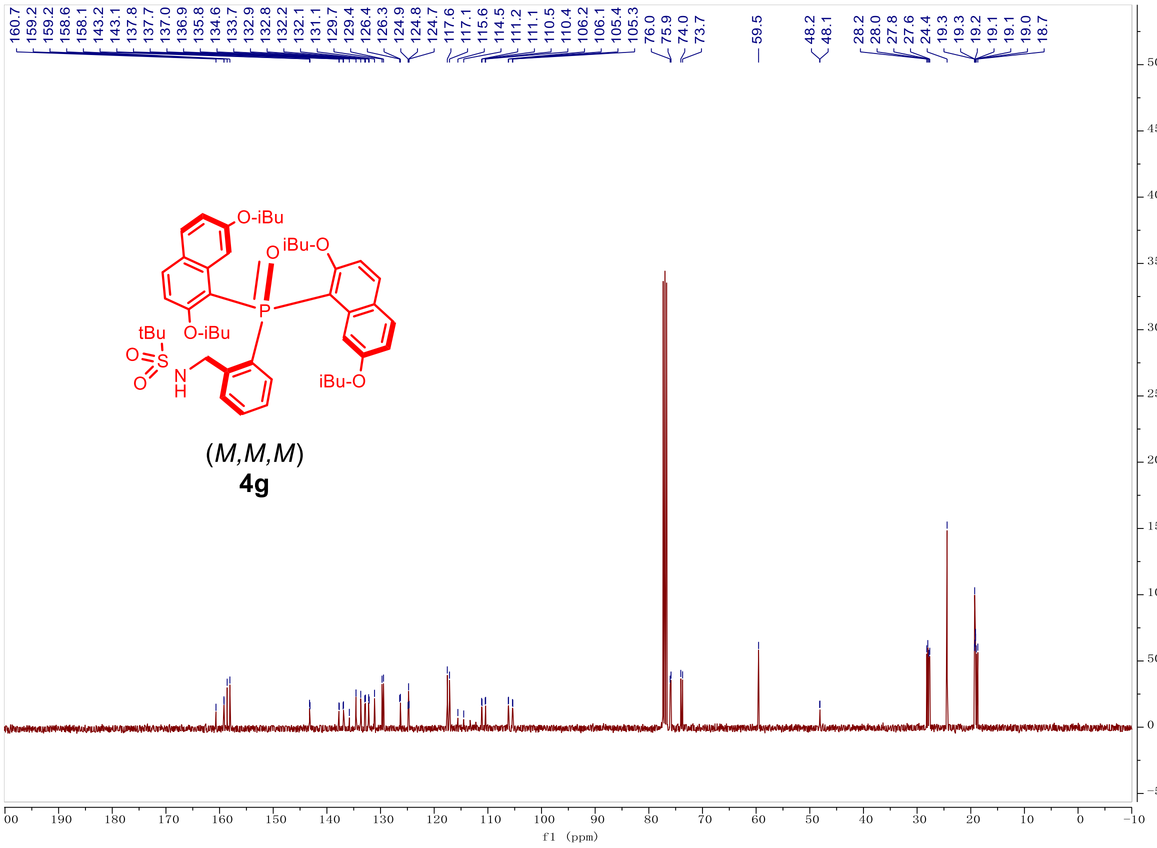
O-iBu
O
iBu-O
P
O-iBu
tBu
O=S
O
N
H
iBu-O
(M,M,M)
4g
f1 (ppm)

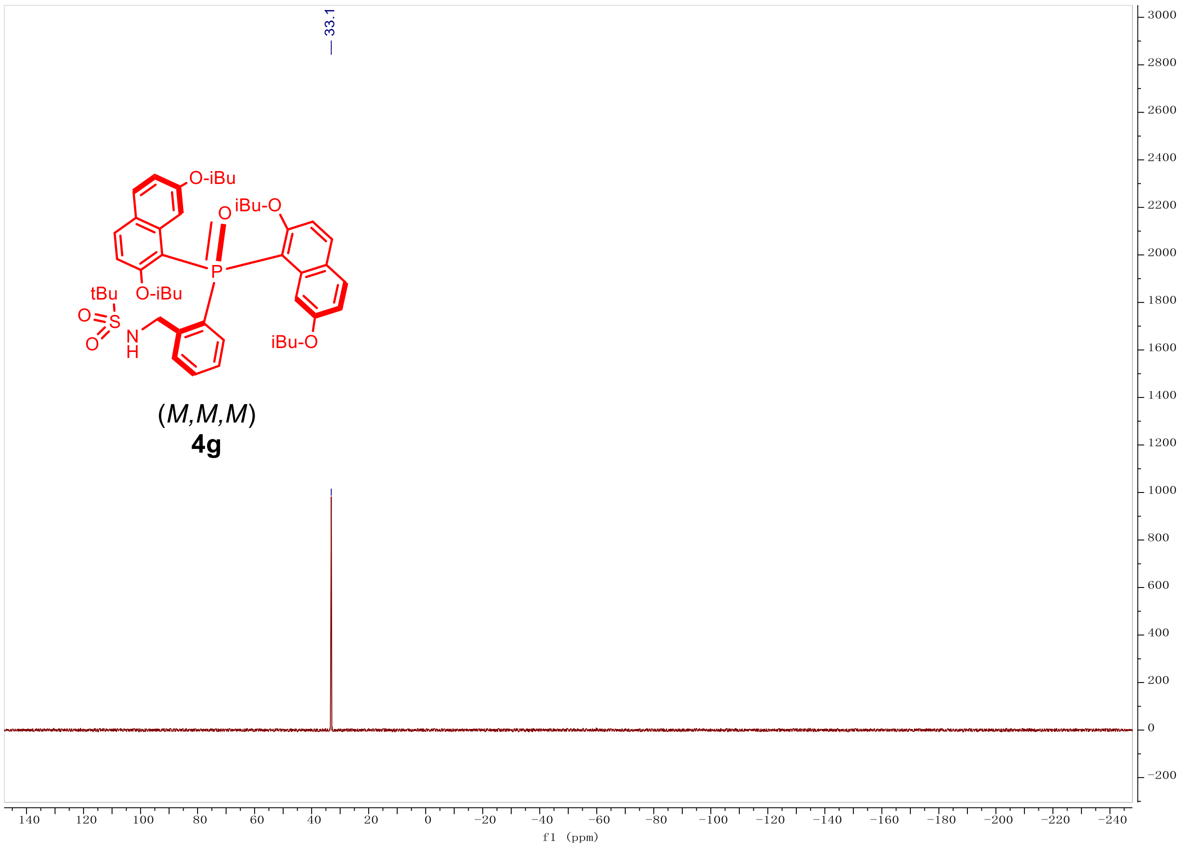
33.1
O-iBu
iBu-O
O
P
tBu
O-iBu
O=S
O
N
H
iBu-O
(M,M,M)
4g
f1 (ppm)

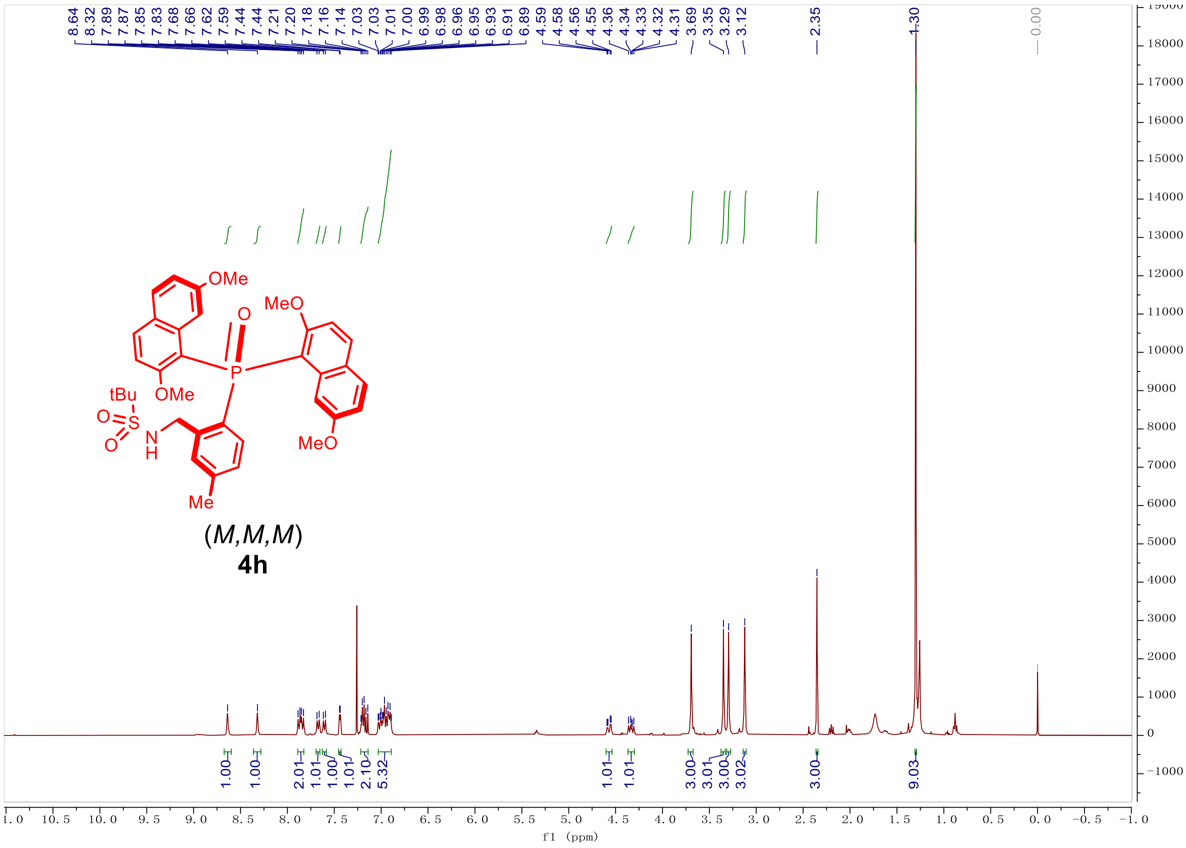
OMe
MeO
O
P
tBu
OMe
O=S
O
N
H
MeO
Me
(M,M,M)
4h
f1 (ppm)

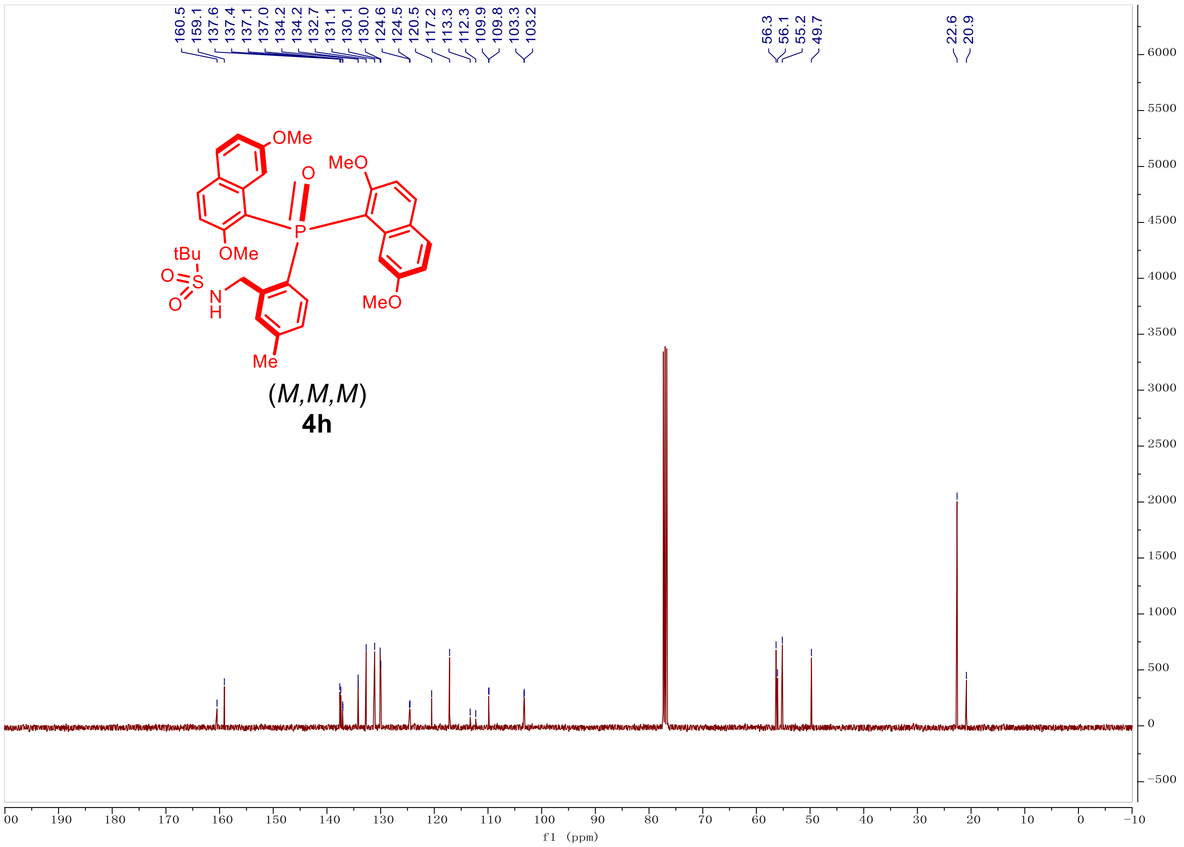
160.5
159.1
137.6
137.4
137.1
137.0
134.2
134.2
132.7
131.1
130.1
130.0
124.6
124.5
120.5
117.2
113.3
112.3
109.9
109.8
103.3
103.2
56.3
56.1
55.2
49.7
22.6
20.9
OMe
MeO
O
P
tBu
OMe
O
S
O
N
H
MeO
Me
(M,M,M)
4h
f1 (ppm)

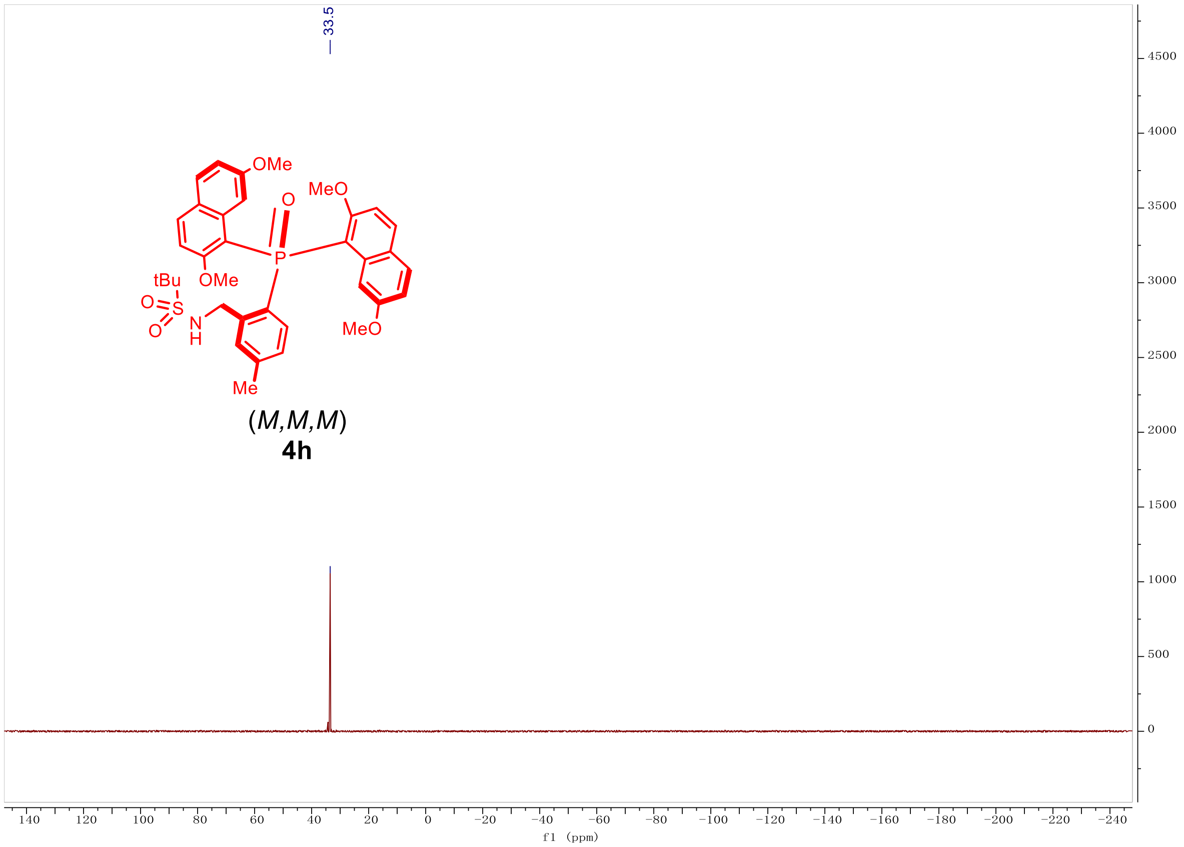
33.5
OMe
MeO
O
P
tBu
OMe
O
S
O
N
H
MeO
Me
(M,M,M)
4h
f1 (ppm)

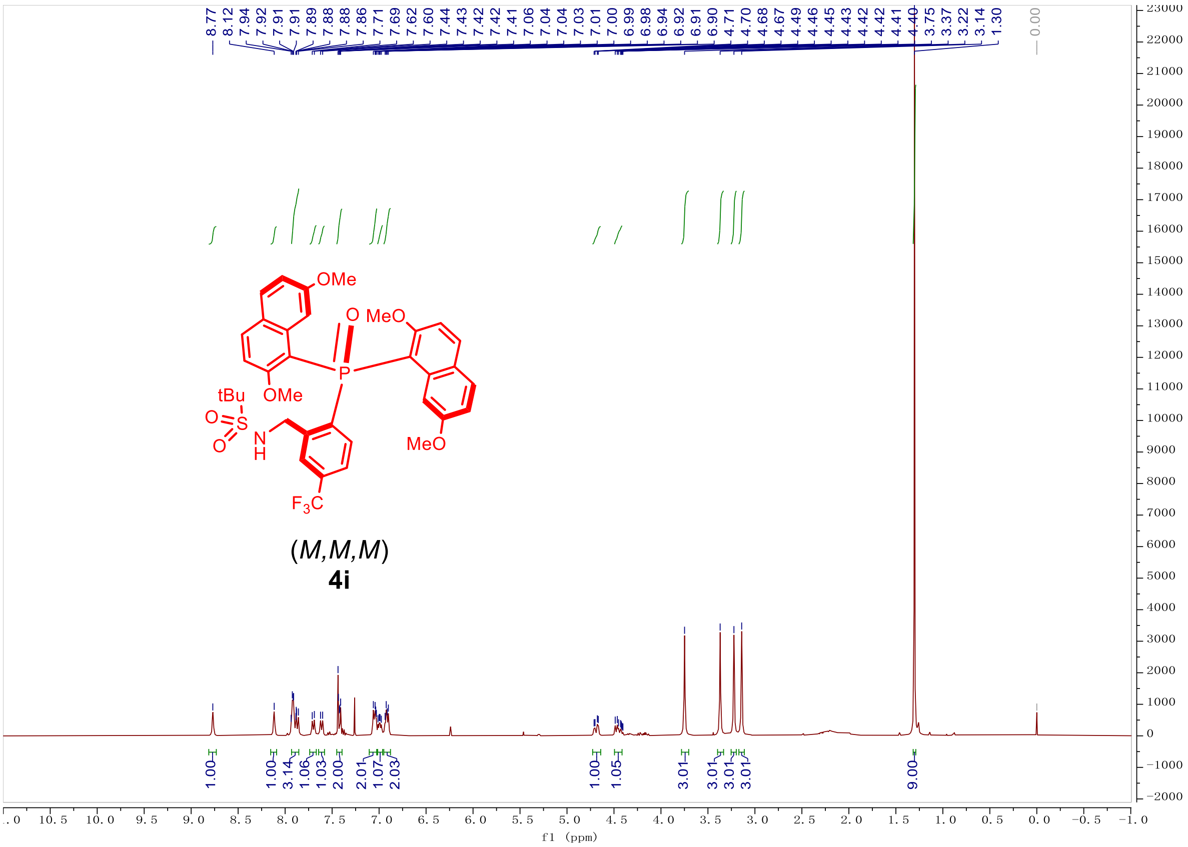
8.77
8.12
7.94
7.92
7.91
7.91
7.89
7.88
7.88
7.86
7.71
7.69
7.62
7.60
7.44
7.43
7.42
7.42
7.41
7.06
7.04
7.04
7.03
7.01
7.00
6.99
6.98
6.94
6.92
6.91
6.90
4.71
4.70
4.68
4.67
4.49
4.46
4.45
4.43
4.42
4.42
4.41
4.40
3.75
3.37
3.22
3.14
1.30
0.00
OMe
O
MeO
tBu
OMe
O
S
O
N
H
P
MeO
F_3C
(*M*,*M*,*M*)
4i
f1 (ppm)

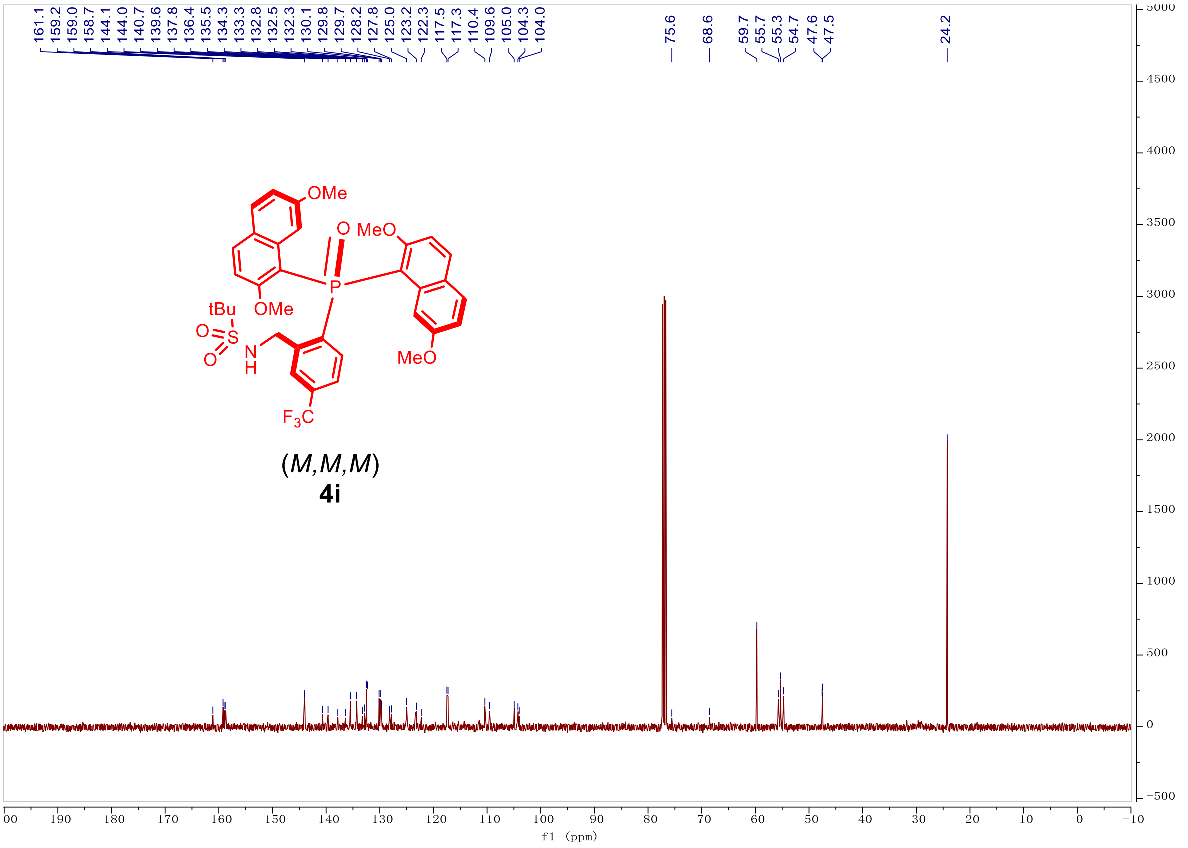
161.1
159.2
159.0
158.7
144.1
144.0
140.7
139.6
137.8
136.4
135.5
134.3
133.3
132.8
132.5
132.3
130.1
129.8
129.7
128.2
127.8
125.0
123.2
122.3
117.5
117.3
110.4
109.6
105.0
104.3
104.0
75.6
68.6
59.7
55.7
55.3
54.7
47.6
47.5
24.2
OMe
O
MeO
tBu
OMe
O
S
O
N
H
P
MeO
F_3C
(*M*,*M*,*M*)
4i
f1 (ppm)

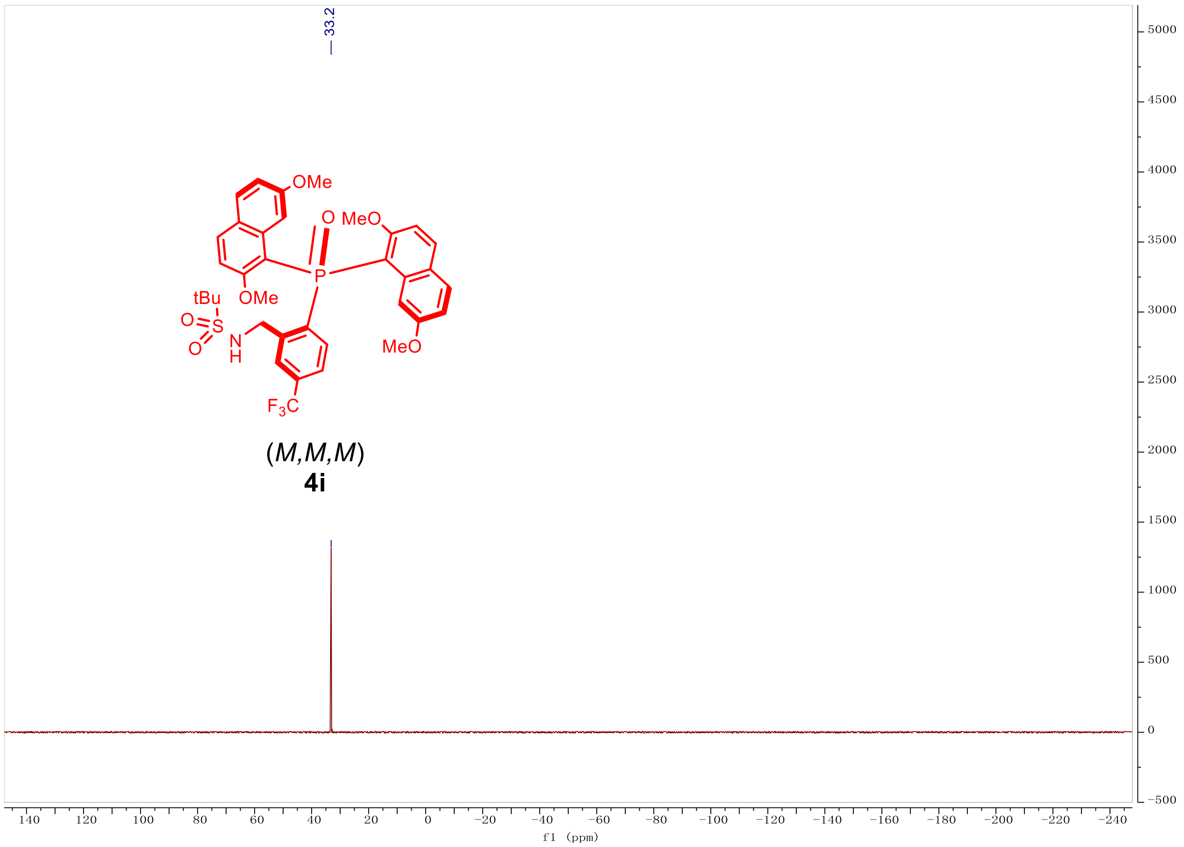
33.2
OMe
O
MeO
OMe
tBu
MeO
(M,M,M)
4i
f1 (ppm)

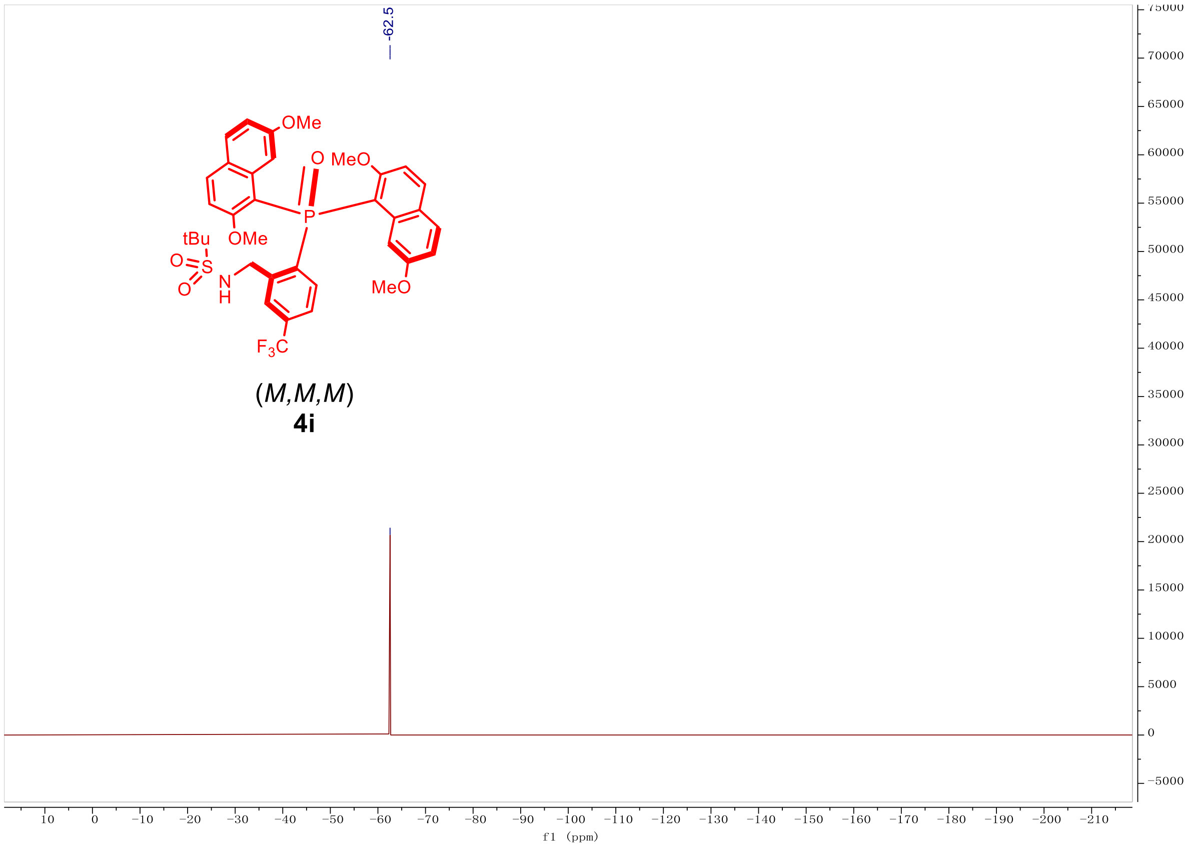
-62.5
OMe
O
MeO
OMe
tBu
MeO
(M,M,M)
4i
f1 (ppm)

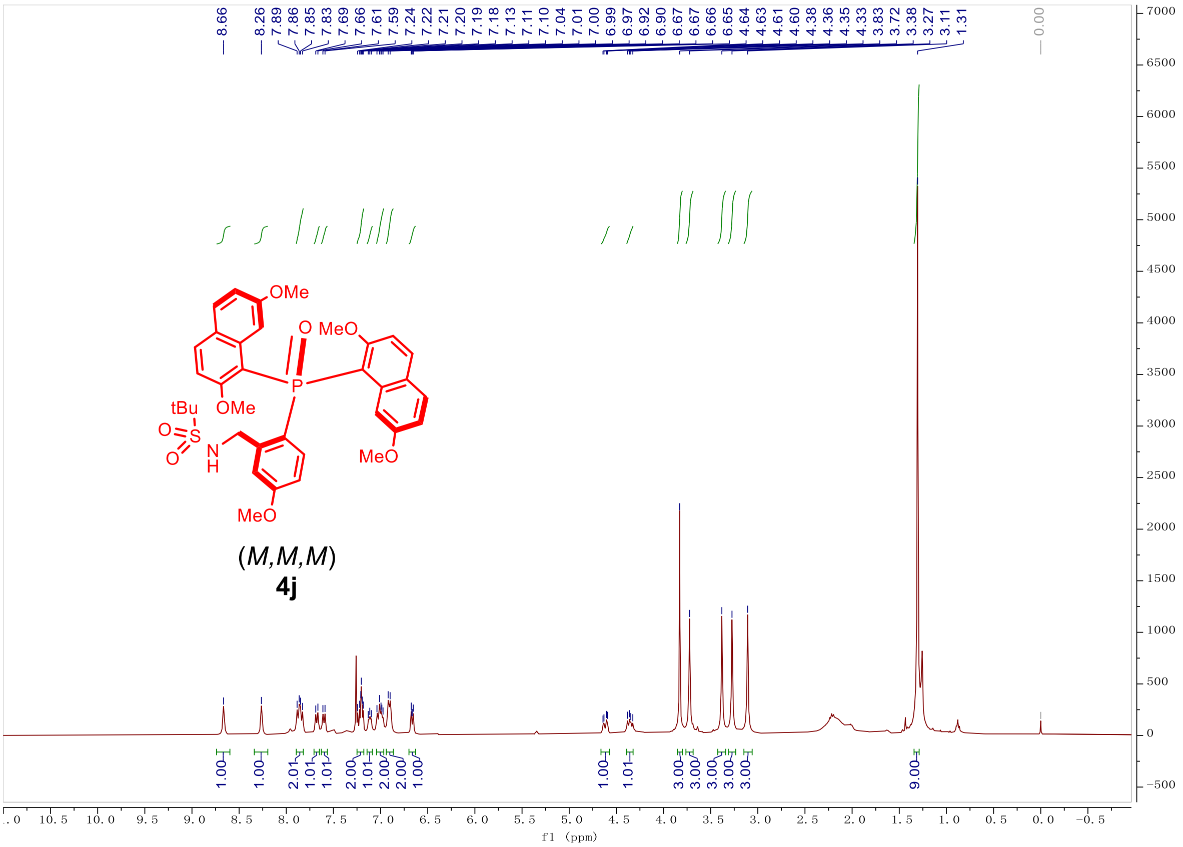

8.66
8.26
7.89
7.86
7.85
7.83
7.69
7.66
7.61
7.59
7.24
7.22
7.21
7.20
7.19
7.18
7.13
7.11
7.10
7.04
7.01
7.00
6.99
6.97
6.92
6.90
6.67
6.67
6.66
6.65
4.64
4.63
4.61
4.60
4.38
4.36
4.35
4.33
3.83
3.72
3.38
3.27
3.11
1.31
0.00
(M,M,M)
4j
f1 (ppm)

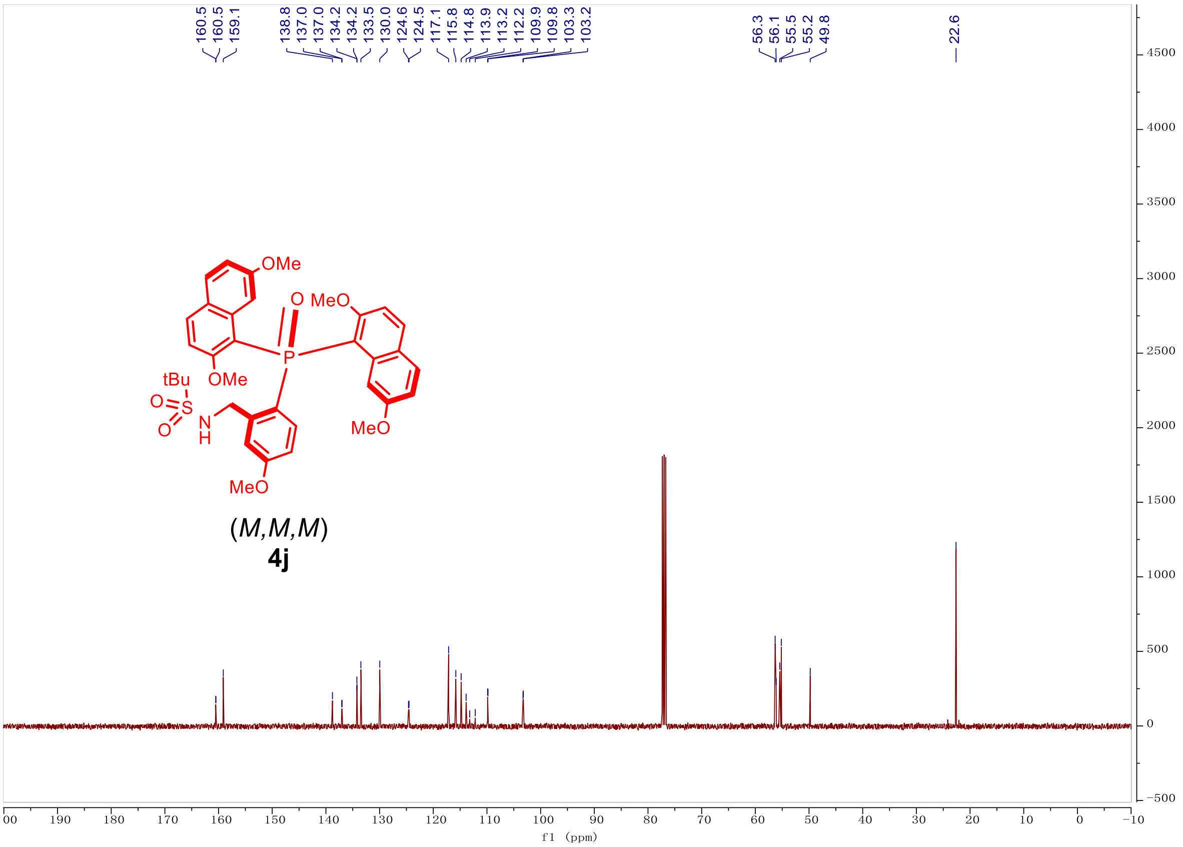

160.5
160.5
159.1
138.8
137.0
137.0
134.2
134.2
133.5
130.0
124.6
124.5
117.1
115.8
114.8
113.9
113.2
112.2
109.9
109.8
103.3
103.2
56.3
56.1
55.5
55.2
49.8
22.6
(M,M,M)
4j
f1 (ppm)

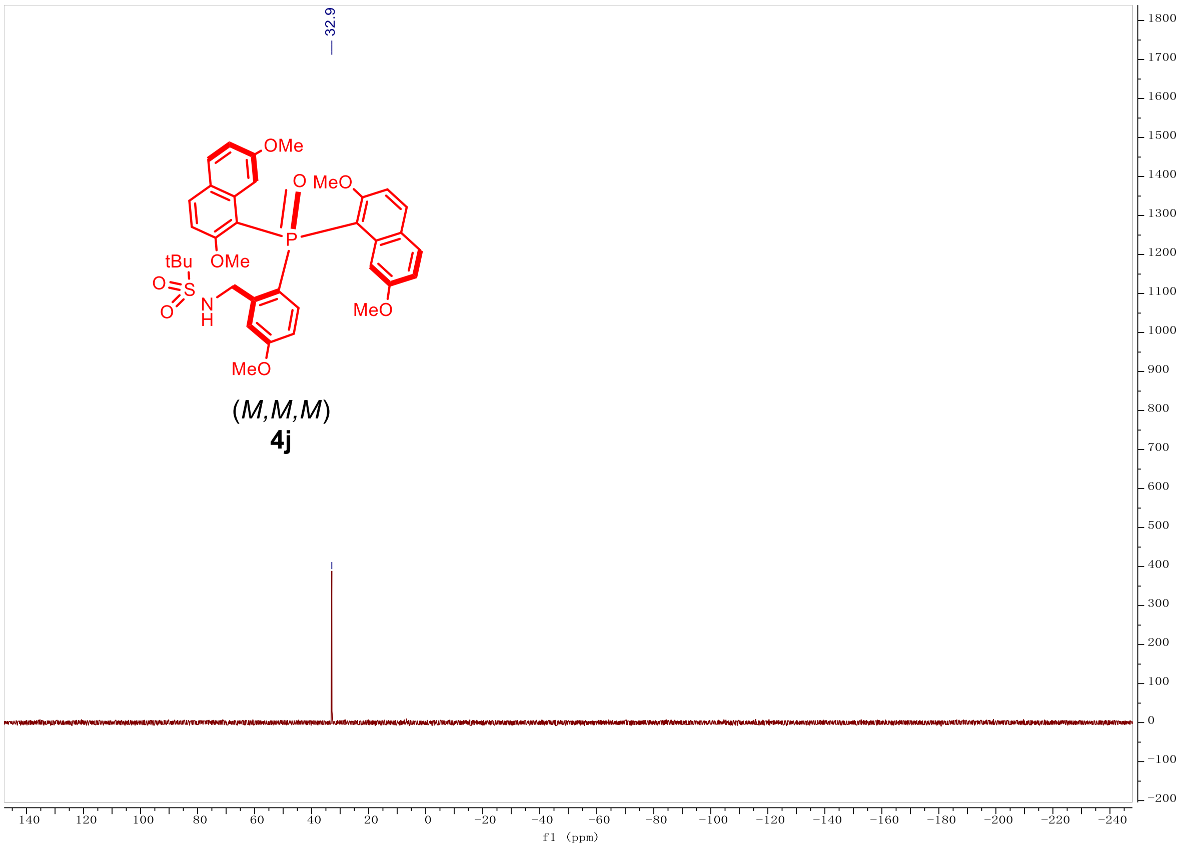
32.9
(M,M,M)
4j
f1 (ppm)

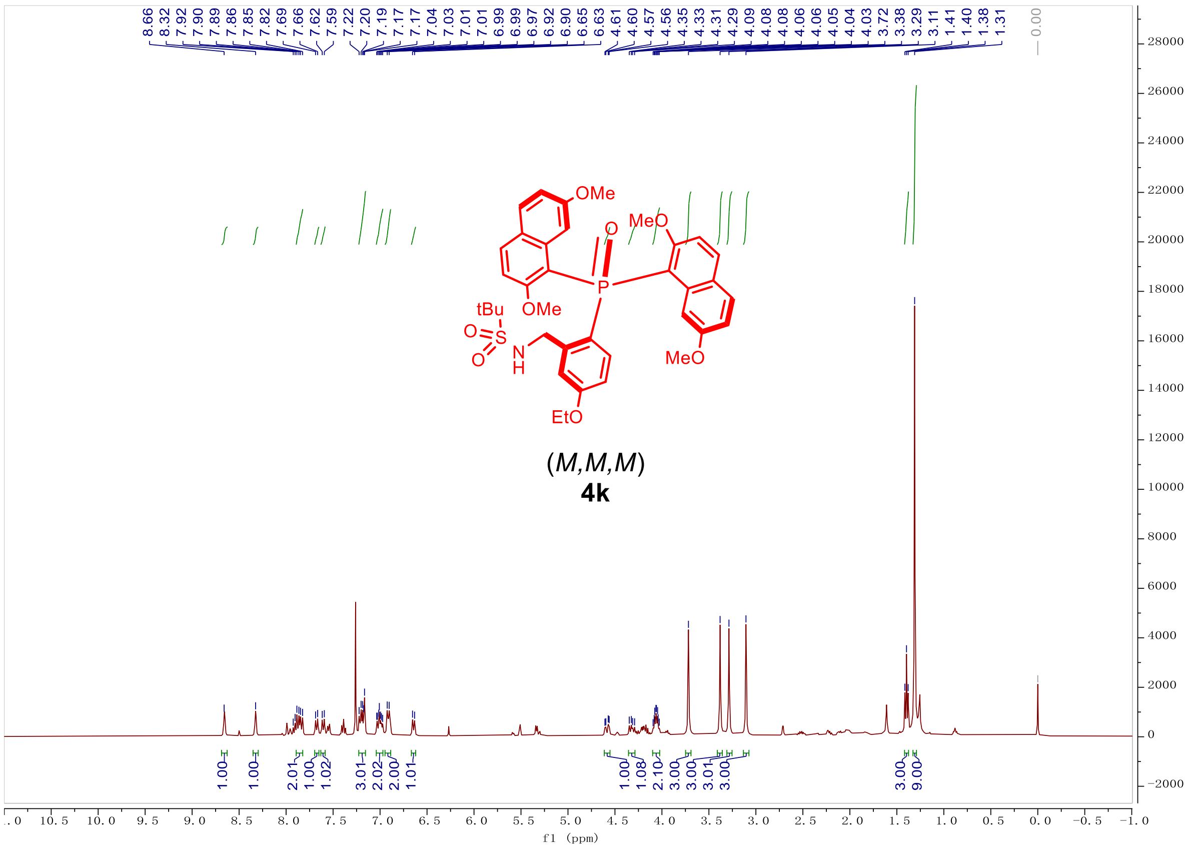
(M,M,M)
4k
f1 (ppm)

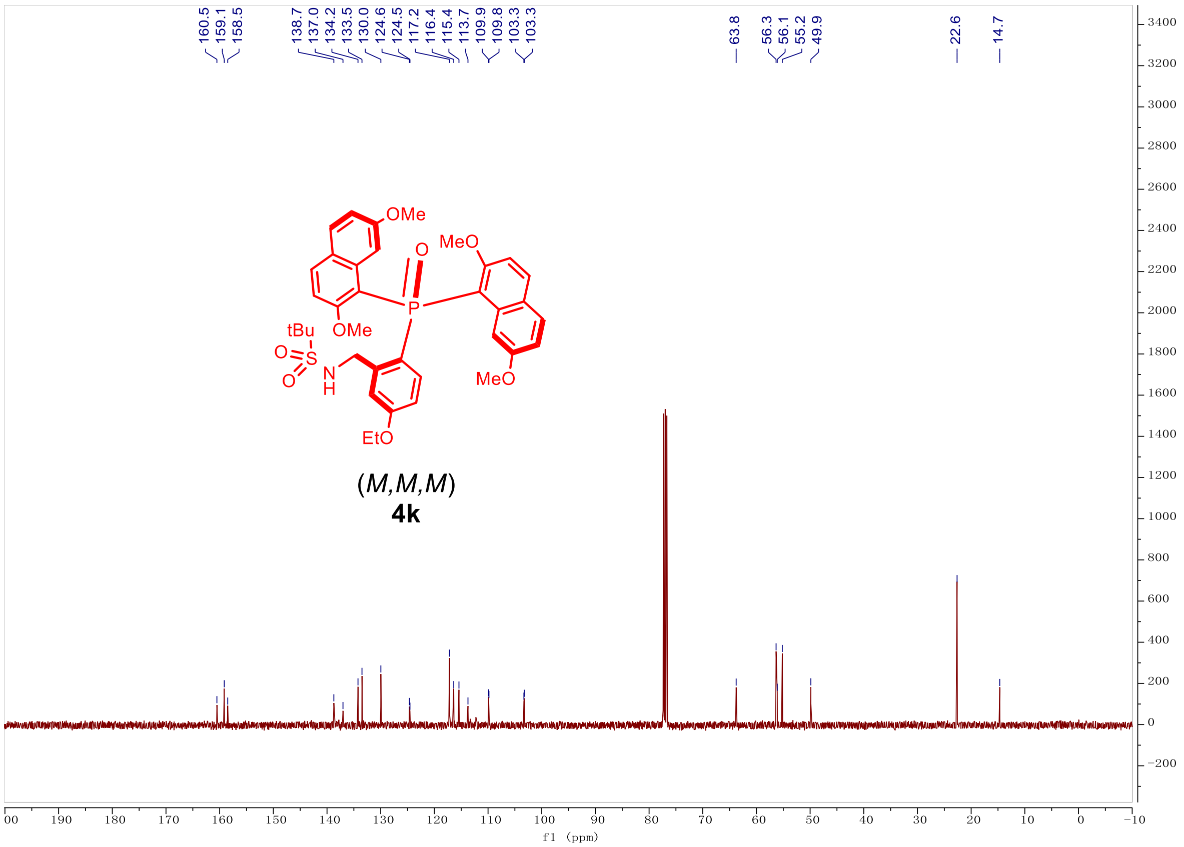
160.5
159.1
158.5
138.7
137.0
134.2
133.5
130.0
124.6
124.5
117.2
116.4
115.4
113.7
109.9
109.8
103.3
103.3
63.8
56.3
56.1
55.2
49.9
22.6
14.7
OMe
MeO
O
P
tBu
OMe
O
S
N
H
O
MeO
EtO
(M,M,M)
4k
f1 (ppm)

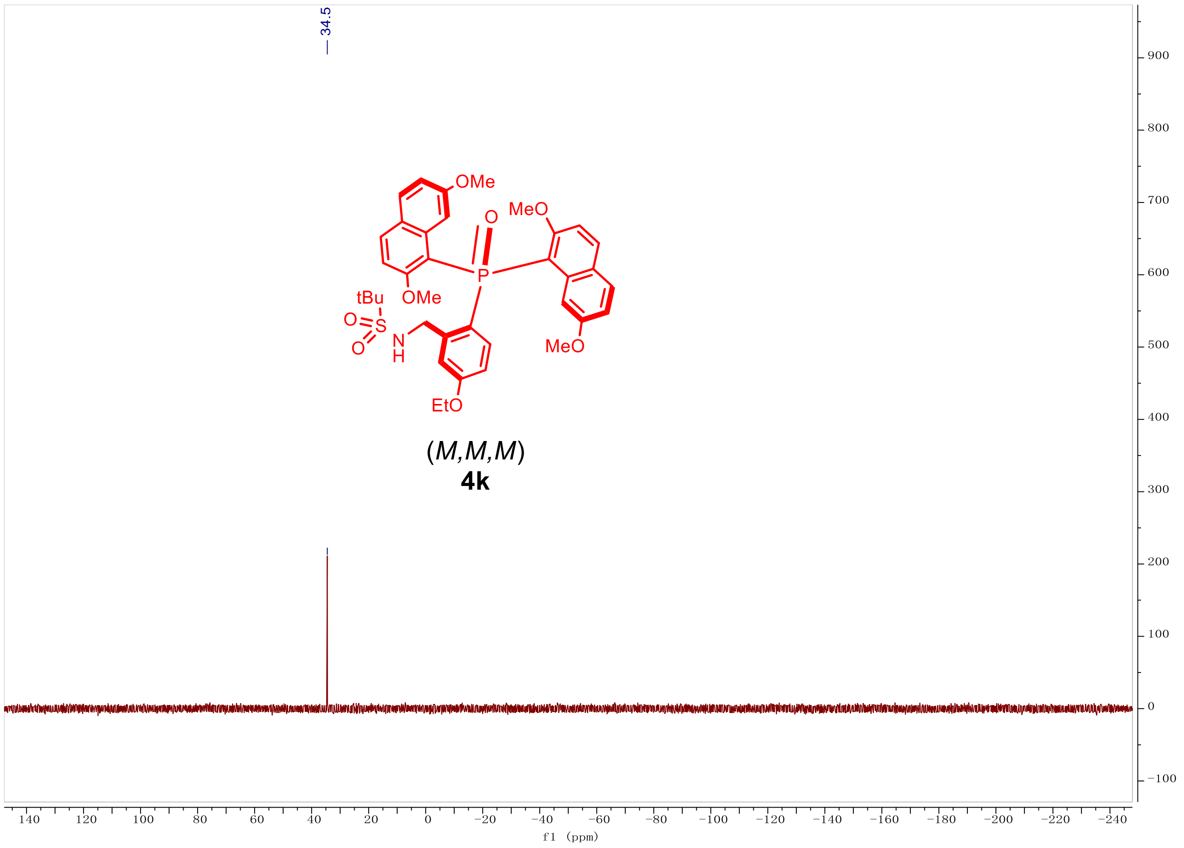
34.5
OMe
MeO
O
P
tBu
OMe
O
S
N
H
O
MeO
EtO
(M,M,M)
4k
f1 (ppm)

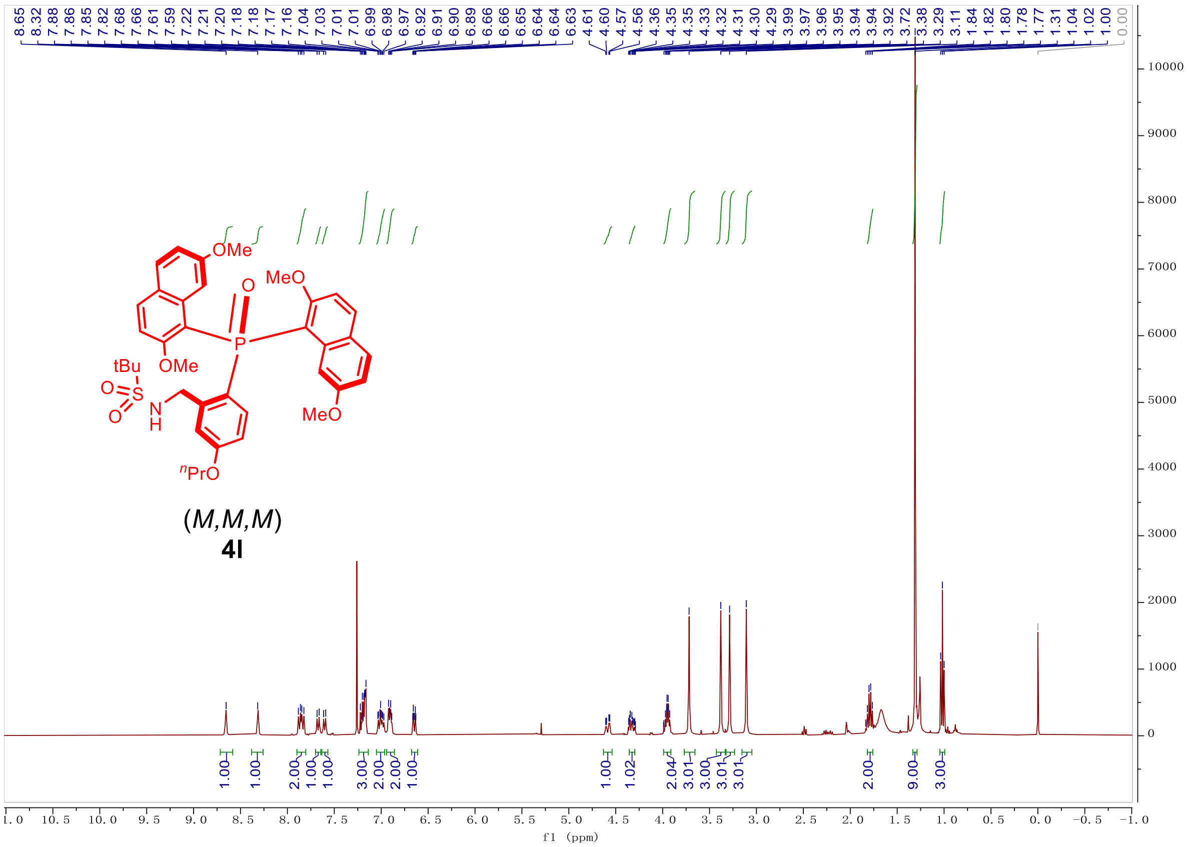

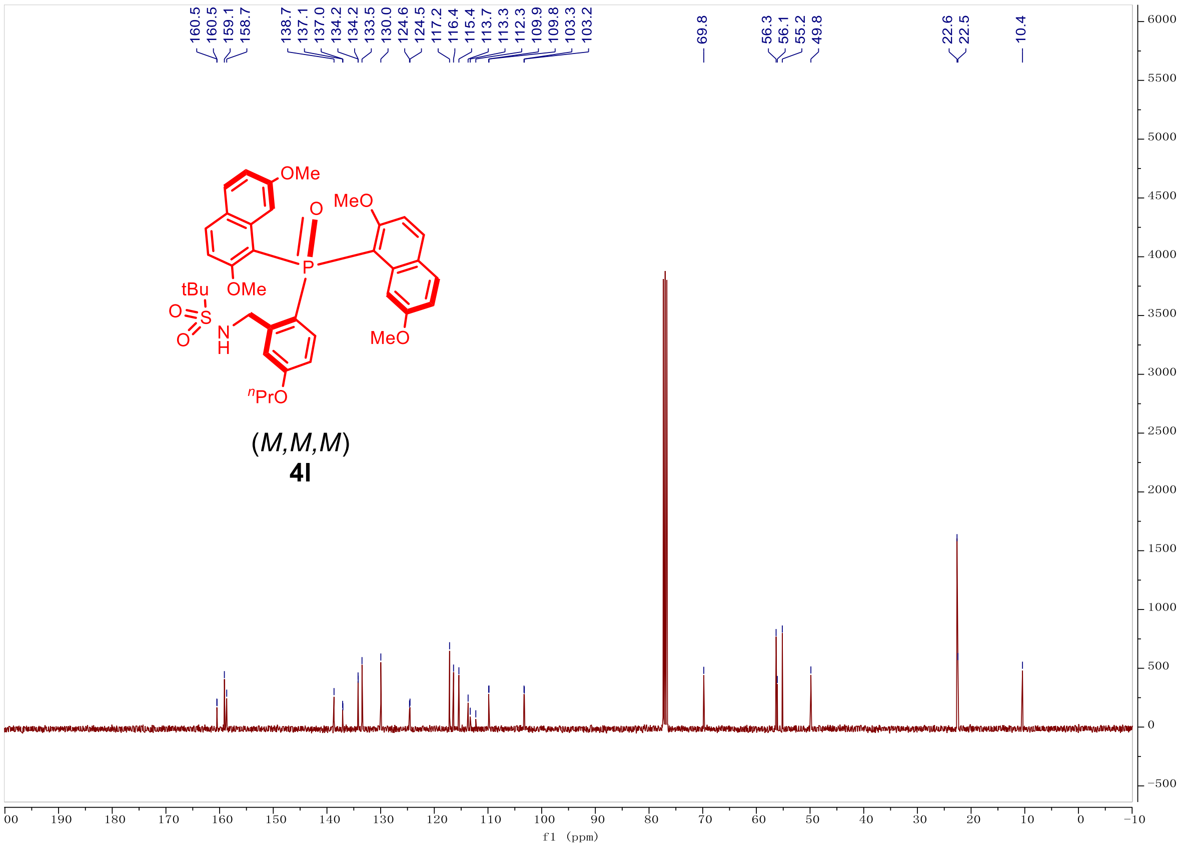

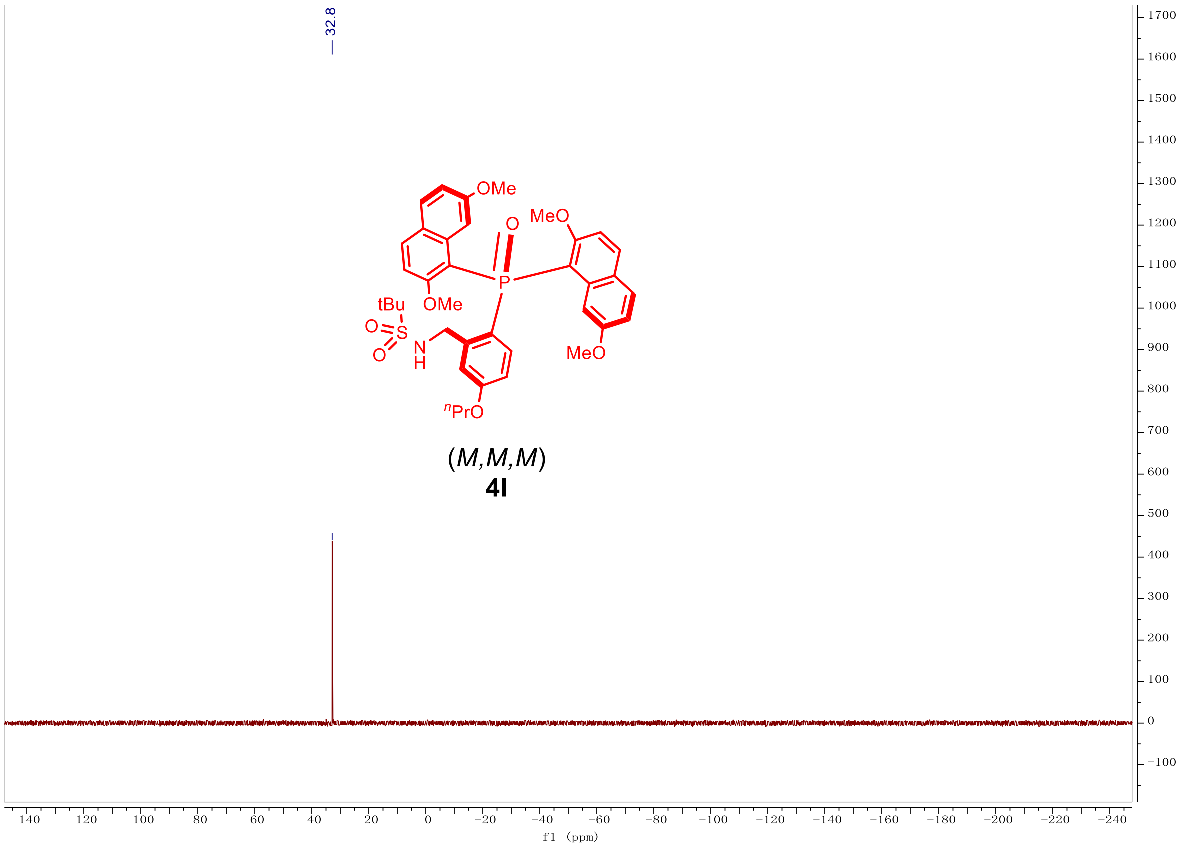

32.8
OMe
MeO
O
P
tBu
OMe
O=S
O
N
H
MeO
^{n}PrO
(M,M,M)
4l
f1 (ppm)

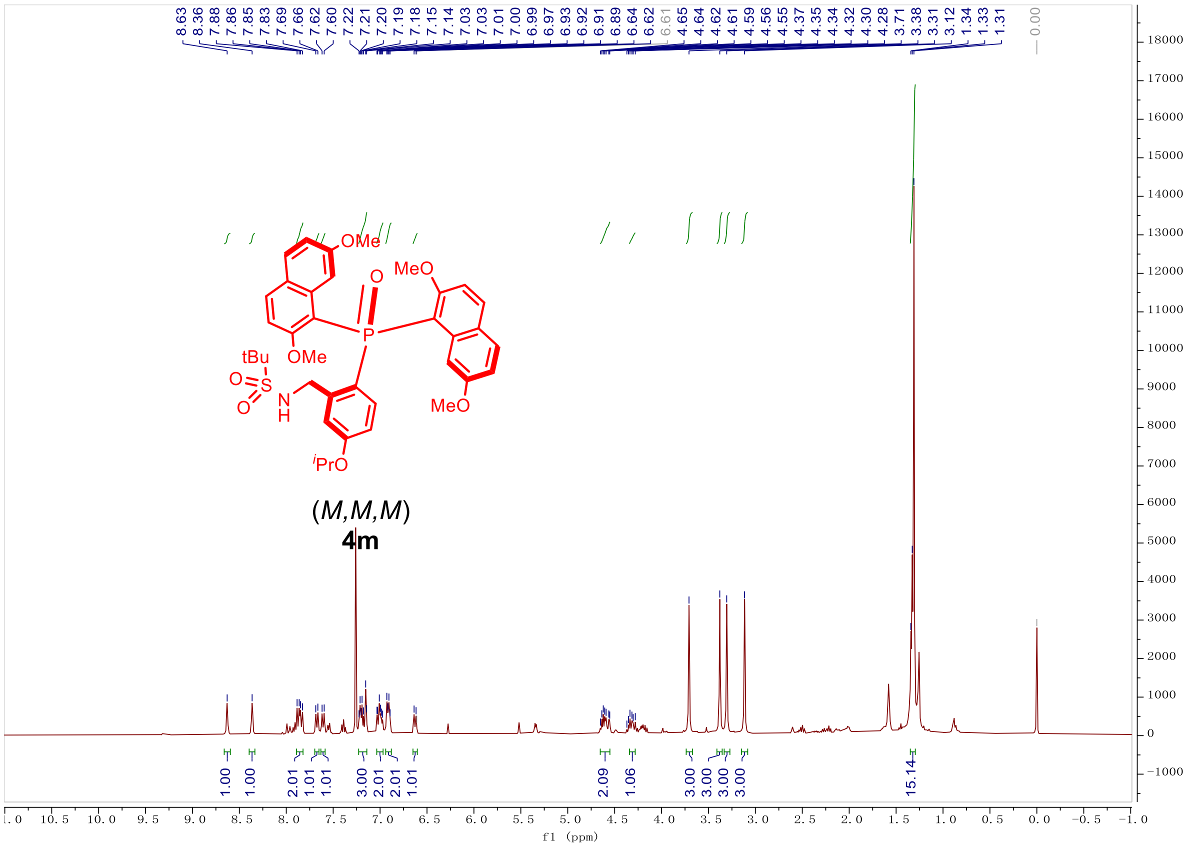

8.63
8.36
7.88
7.86
7.85
7.83
7.69
7.66
7.62
7.60
7.22
7.21
7.20
7.19
7.18
7.15
7.14
7.03
7.01
7.00
6.99
6.97
6.93
6.92
6.91
6.89
6.64
6.62
6.61
4.65
4.64
4.62
4.61
4.59
4.56
4.55
4.37
4.35
4.34
4.32
4.30
4.28
3.71
3.38
3.31
3.12
1.34
1.33
1.31
0.00
OMe
MeO
O
P
tBu
OMe
O=S
O
N
H
MeO
^{i}PrO
(M,M,M)
4m
1.00
1.00
2.01
1.01
1.01
3.00
2.01
2.01
1.01
2.09
1.06
3.00
3.00
3.00
3.00
15.14
f1 (ppm)

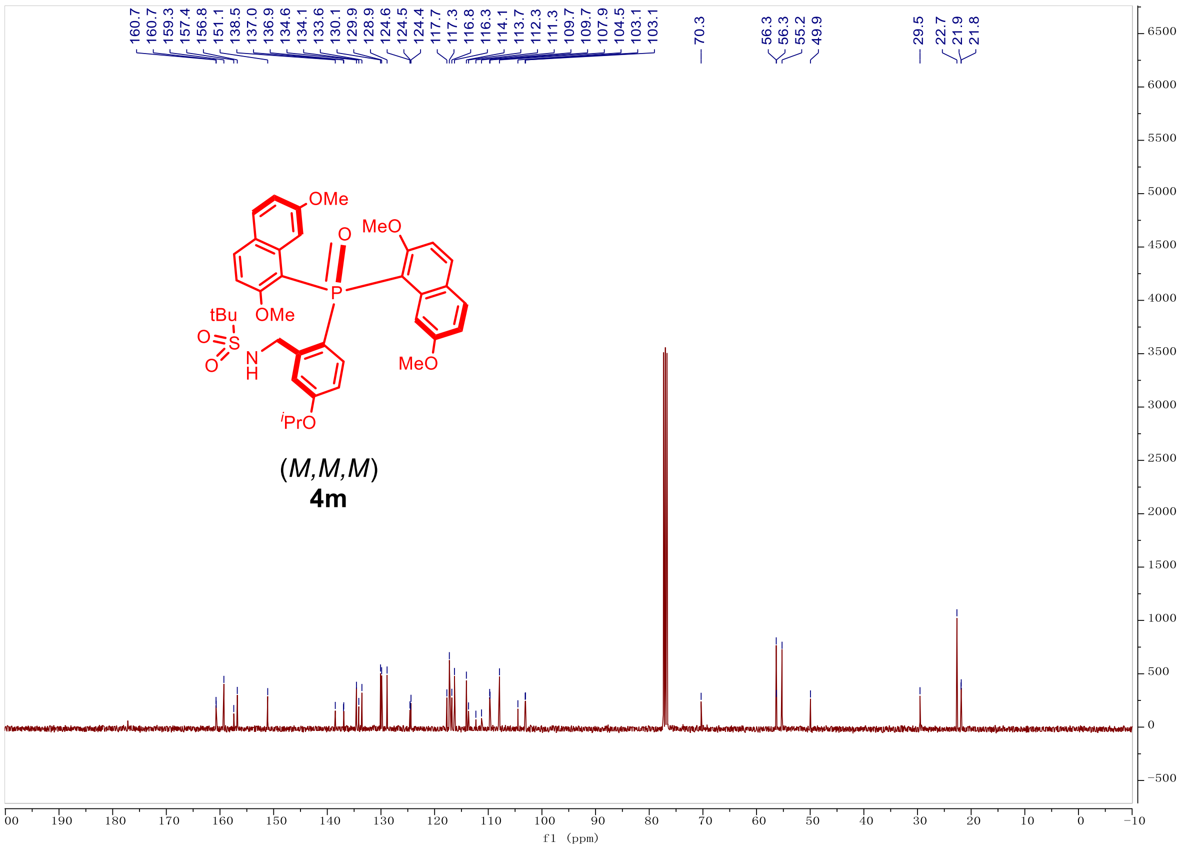
160.7
160.7
159.3
157.4
156.8
151.1
138.5
137.0
136.9
134.6
134.1
133.6
130.1
129.9
128.9
124.6
124.5
124.4
117.7
117.3
116.8
116.3
114.1
113.7
112.3
111.3
109.7
109.7
107.9
104.5
103.1
103.1
70.3
56.3
56.3
55.2
49.9
29.5
22.7
21.9
21.8
OMe
O
MeO
P
tBu
OMe
O=S
O
N
H
MeO
iPrO
(M,M,M)
4m
f1 (ppm)

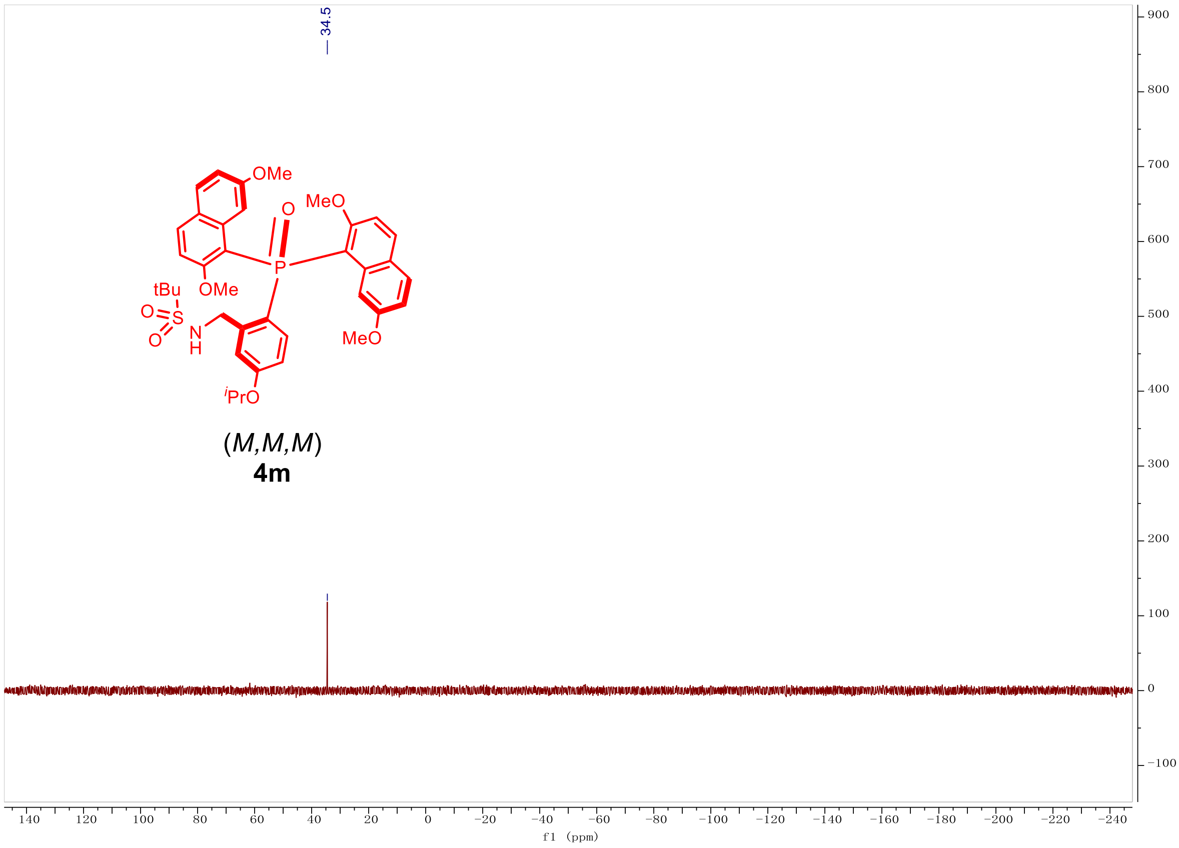
34.5
OMe
O
MeO
P
tBu
OMe
O=S
O
N
H
MeO
iPrO
(M,M,M)
4m
f1 (ppm)

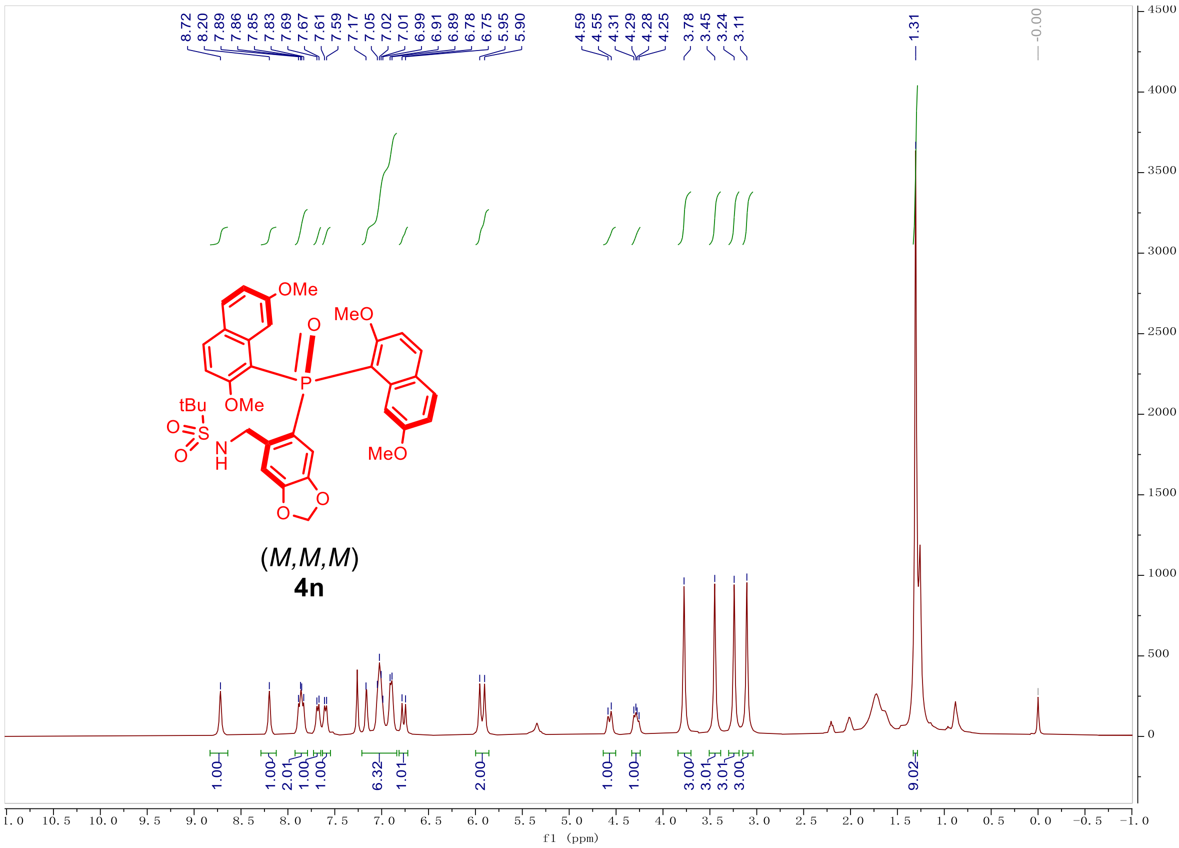
8.72
8.20
7.89
7.86
7.85
7.83
7.69
7.67
7.61
7.59
7.17
7.05
7.02
7.01
6.99
6.91
6.89
6.78
6.75
5.95
5.90
4.59
4.55
4.31
4.29
4.28
4.25
3.78
3.45
3.24
3.11
1.31
-0.00
OMe
MeO
O
P
tBu
OMe
O=S
O
N
H
MeO
O
O
(M,M,M)
4n
1.00
1.00
2.01
1.00
1.00
6.32
1.01
2.00
1.00
1.00
3.00
3.01
3.01
3.00
9.02
f1 (ppm)

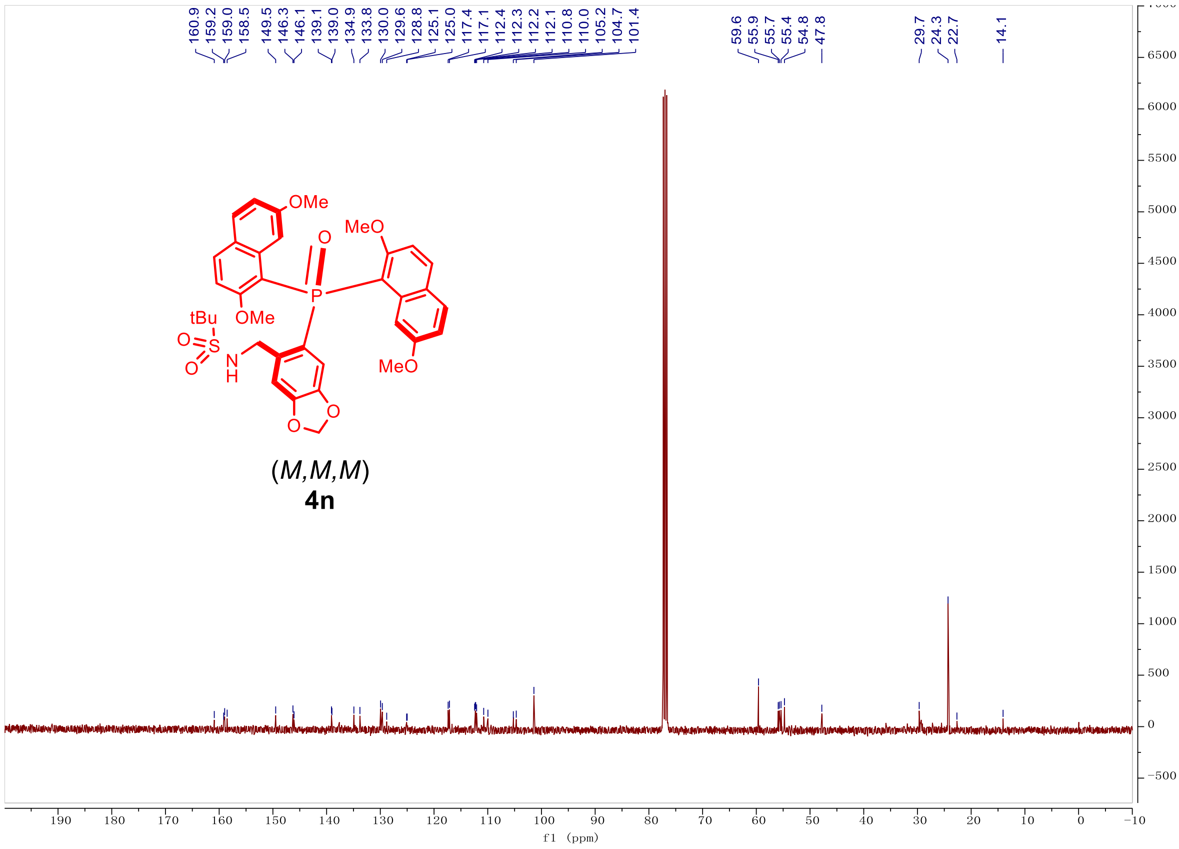
160.9
159.2
159.0
158.5
149.5
146.3
146.1
139.1
139.0
134.9
133.8
130.0
129.6
128.8
125.1
125.0
117.4
117.1
112.4
112.3
112.2
112.1
110.8
110.0
105.2
104.7
101.4
59.6
55.9
55.7
55.4
54.8
47.8
29.7
24.3
22.7
14.1
OMe
MeO
O
P
tBu
OMe
O=S
O
N
H
MeO
O
O
(M,M,M)
4n
f1 (ppm)

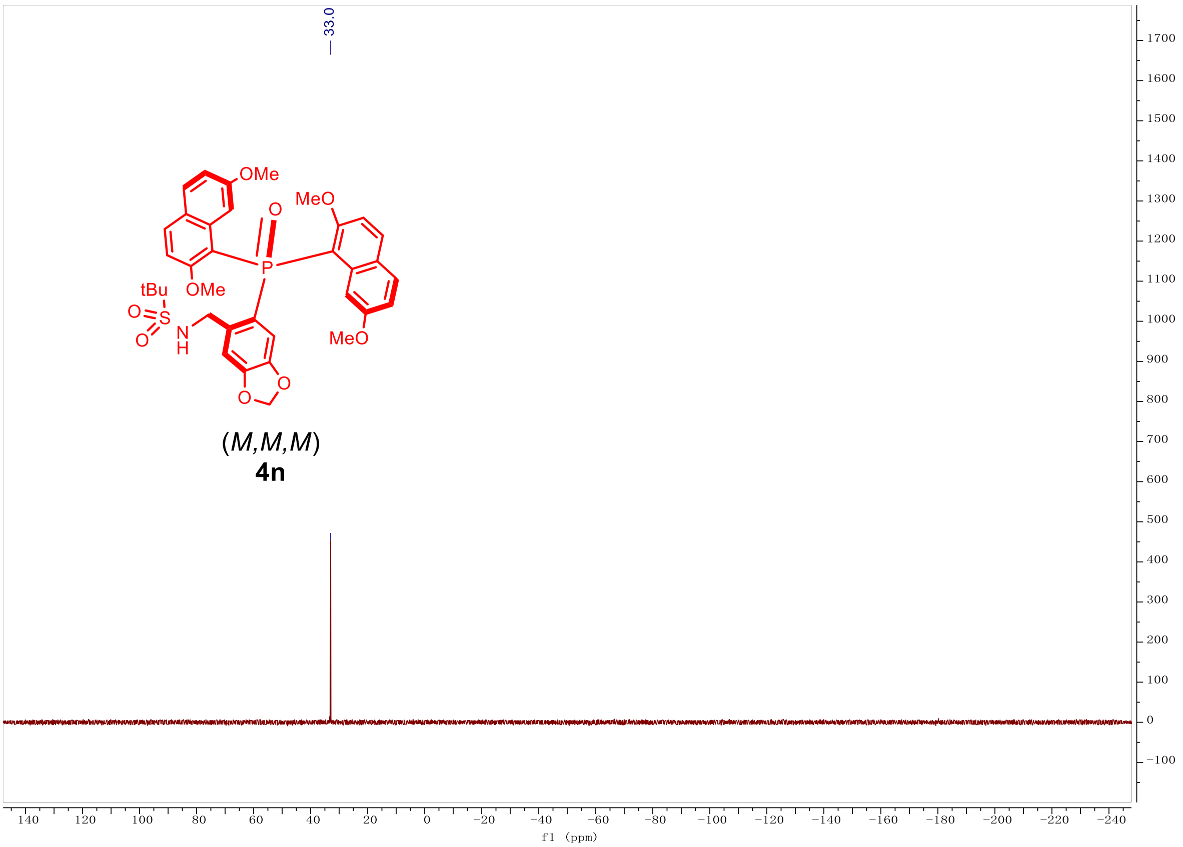

(*M*,*M*,*M*)
4n

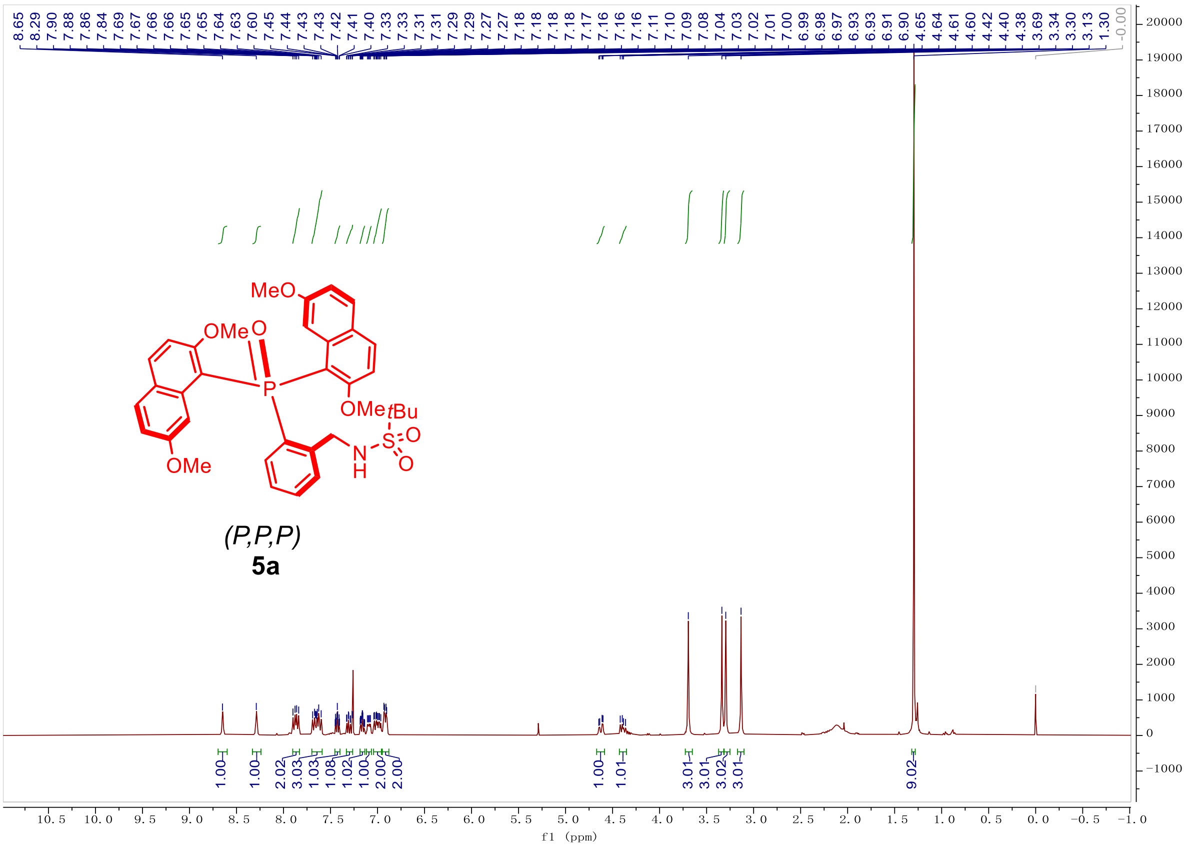

(*P*,*P*,*P*)
5a

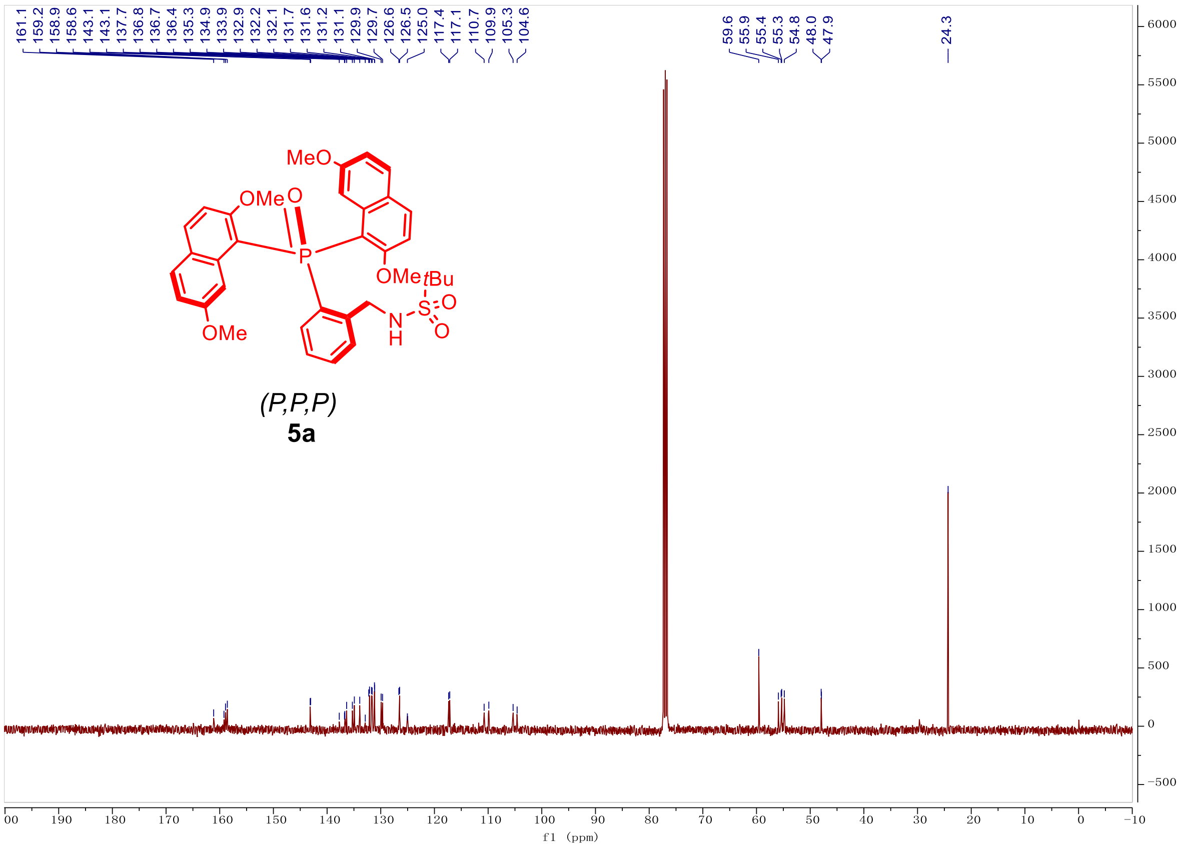
161.1
159.2
158.9
158.6
143.1
143.1
137.7
136.8
136.7
136.4
135.3
134.9
133.9
132.9
132.2
132.1
131.7
131.6
131.2
131.1
129.9
129.7
126.6
126.5
125.0
117.4
117.1
110.7
109.9
105.3
104.6
59.6
55.9
55.4
55.3
54.8
48.0
47.9
24.3
MeO
OMe
O
P
OMe
tBu
S
O
O
N
H
OMe
(P,P,P)
5a
f1 (ppm)

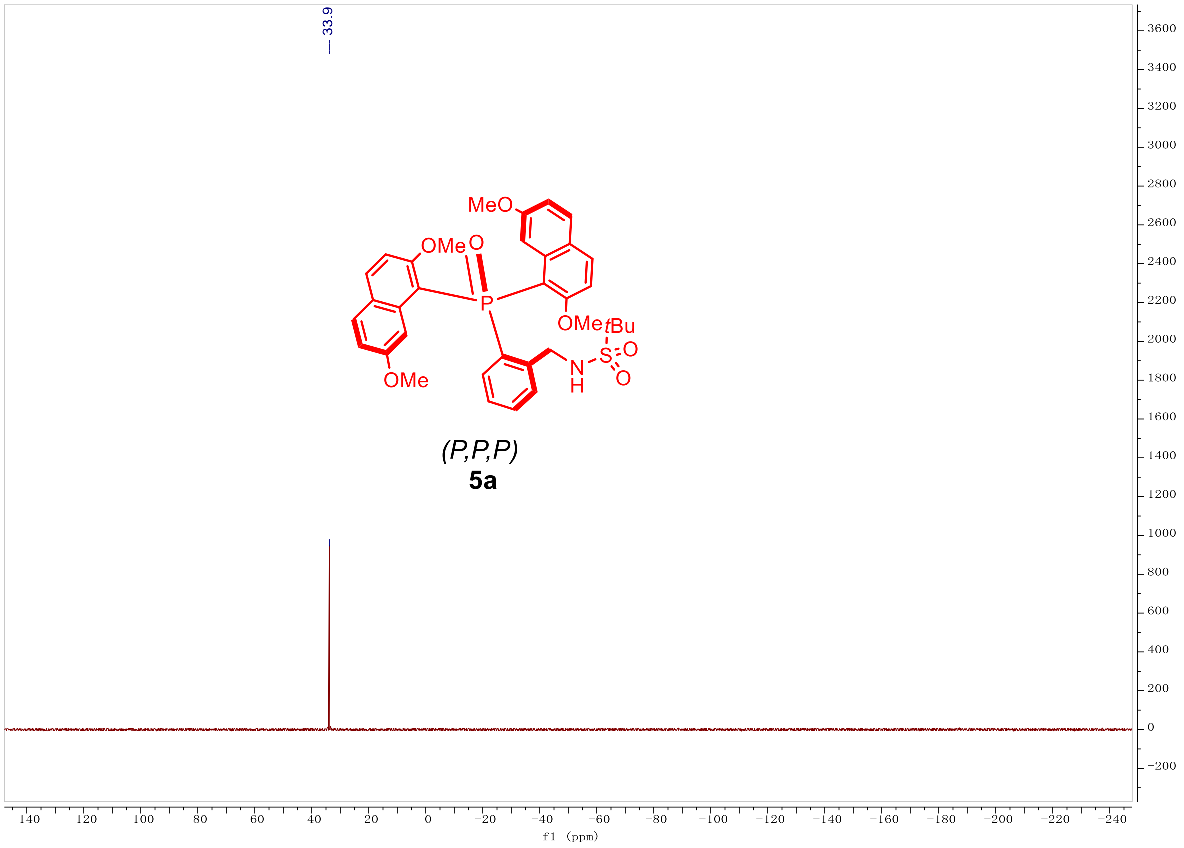
33.9
MeO
OMe
O
P
OMe
tBu
S
O
O
N
H
OMe
(P,P,P)
5a
f1 (ppm)

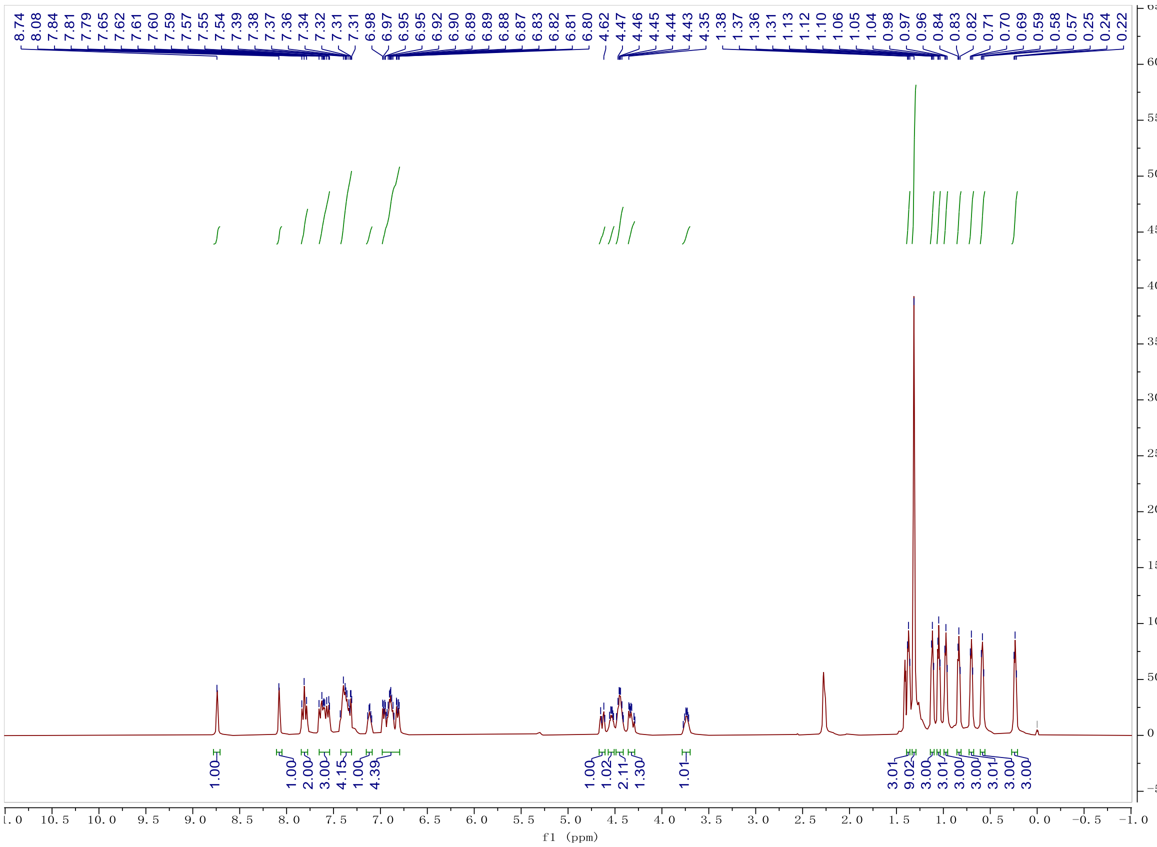

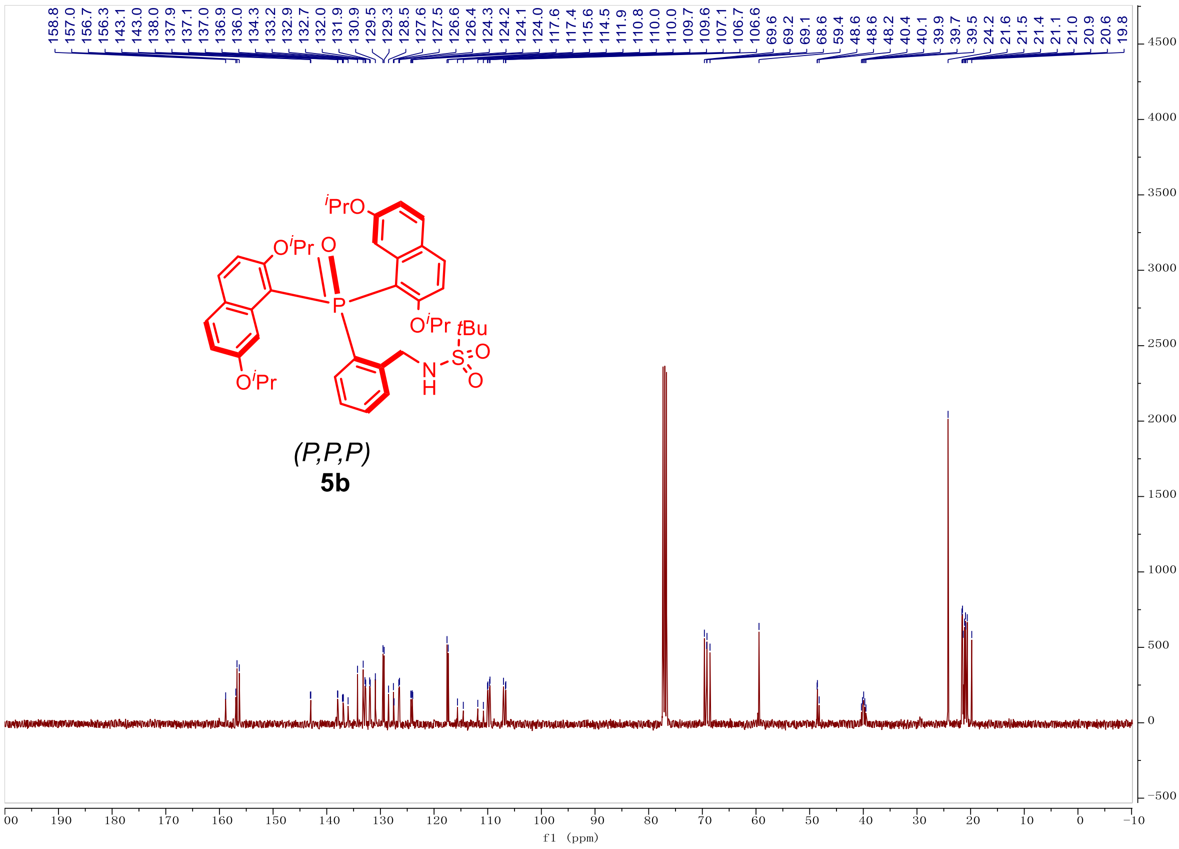

(P,P,P)

5b

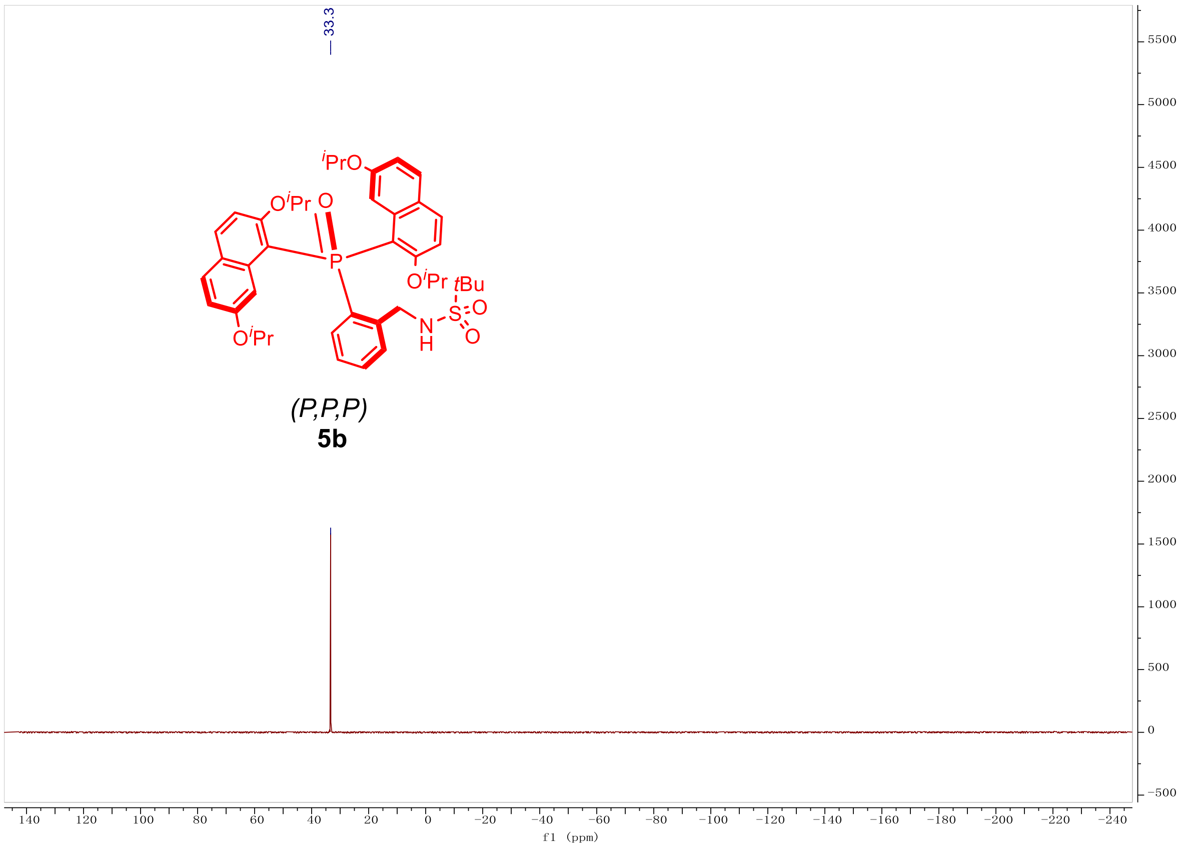
33.3
(P,P,P)
5b
f1 (ppm)

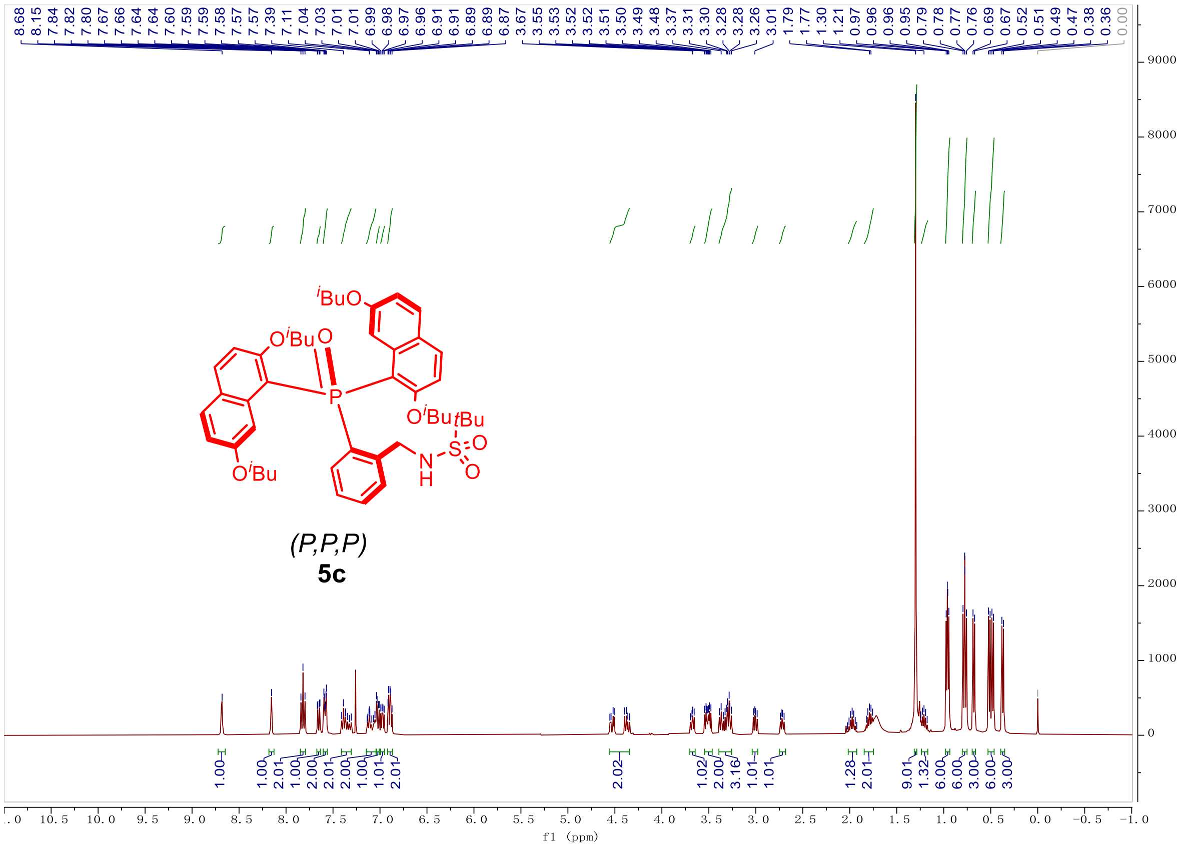
(P,P,P)
5c
f1 (ppm)

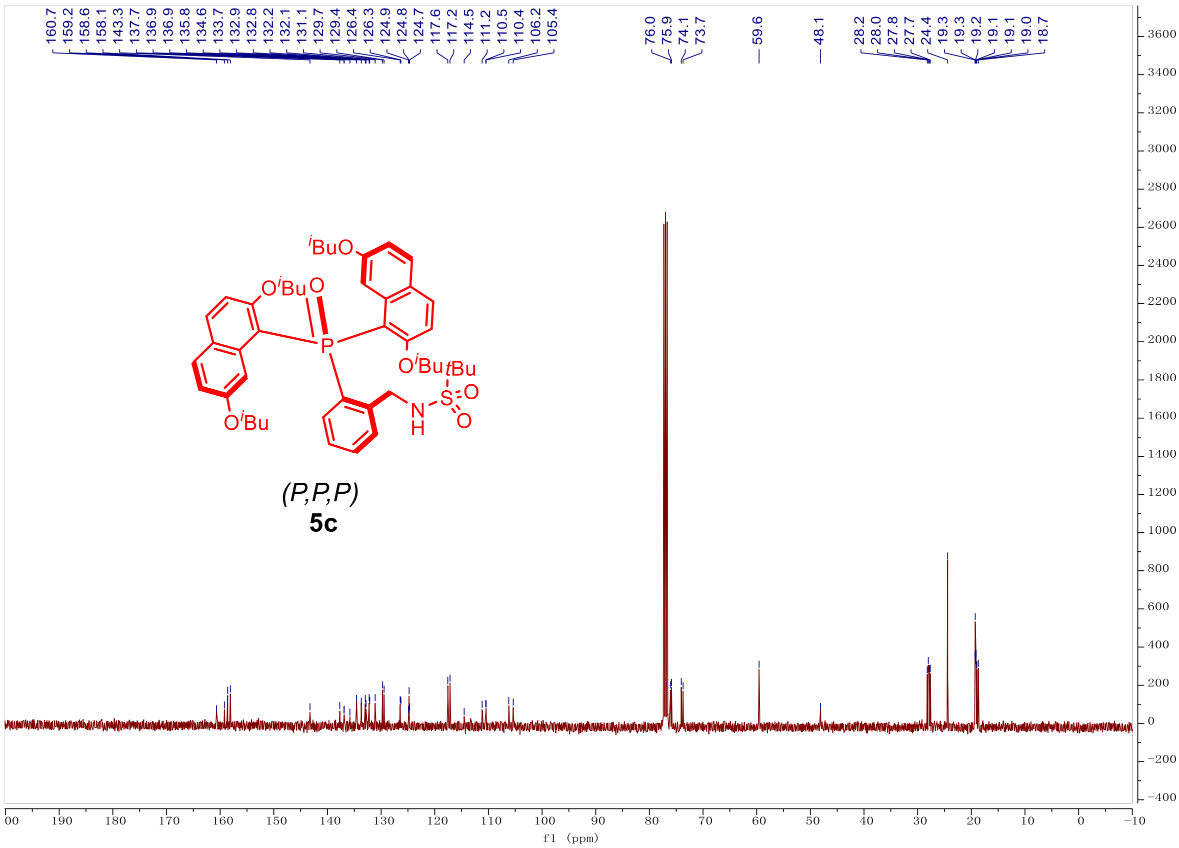
160.7
159.2
158.6
158.1
143.3
137.7
136.9
136.9
135.8
134.6
133.7
132.9
132.8
132.2
132.1
131.1
129.7
129.4
126.4
126.3
124.9
124.8
124.7
117.6
117.2
114.5
111.2
110.5
110.4
106.2
105.4
76.0
75.9
74.1
73.7
59.6
48.1
28.2
28.0
27.8
27.7
24.4
19.3
19.2
19.1
19.1
19.0
18.7
(P,P,P)
5c
f1 (ppm)

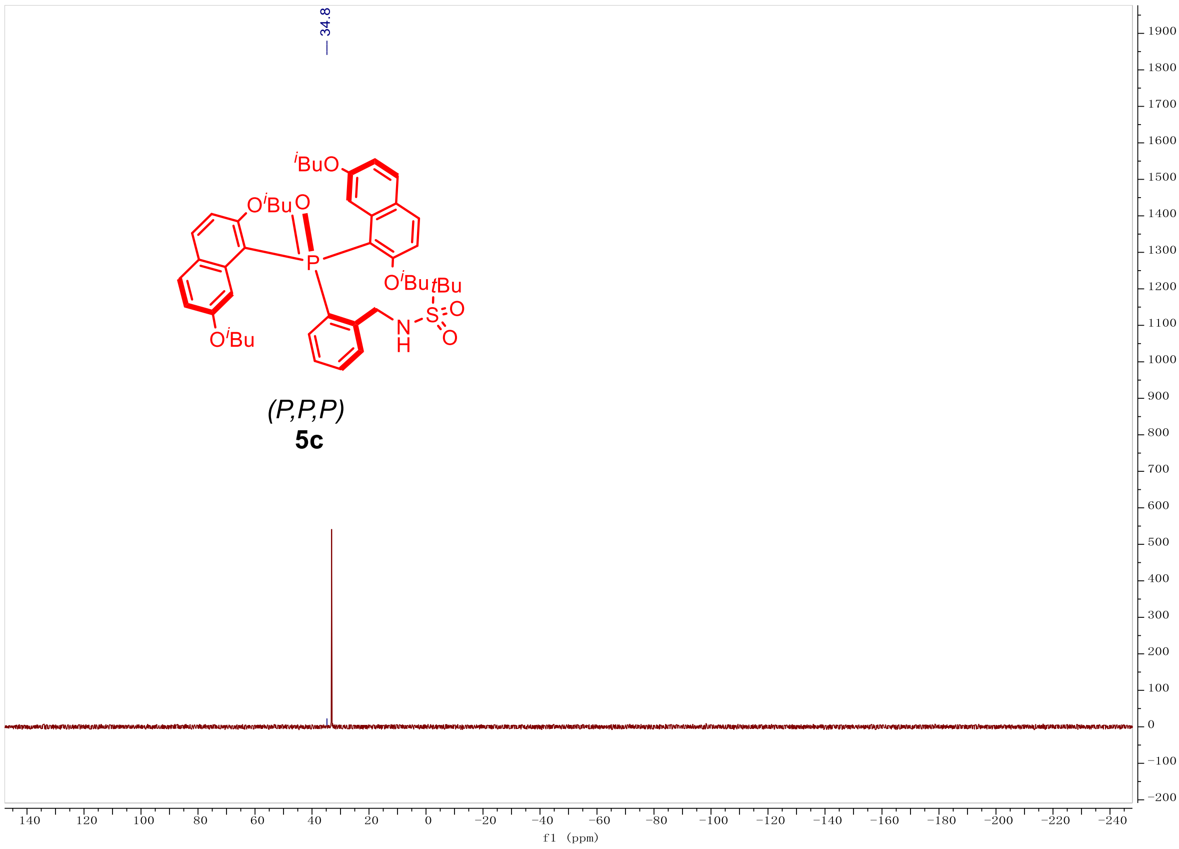
34.8
(P,P,P)
5c
f1 (ppm)

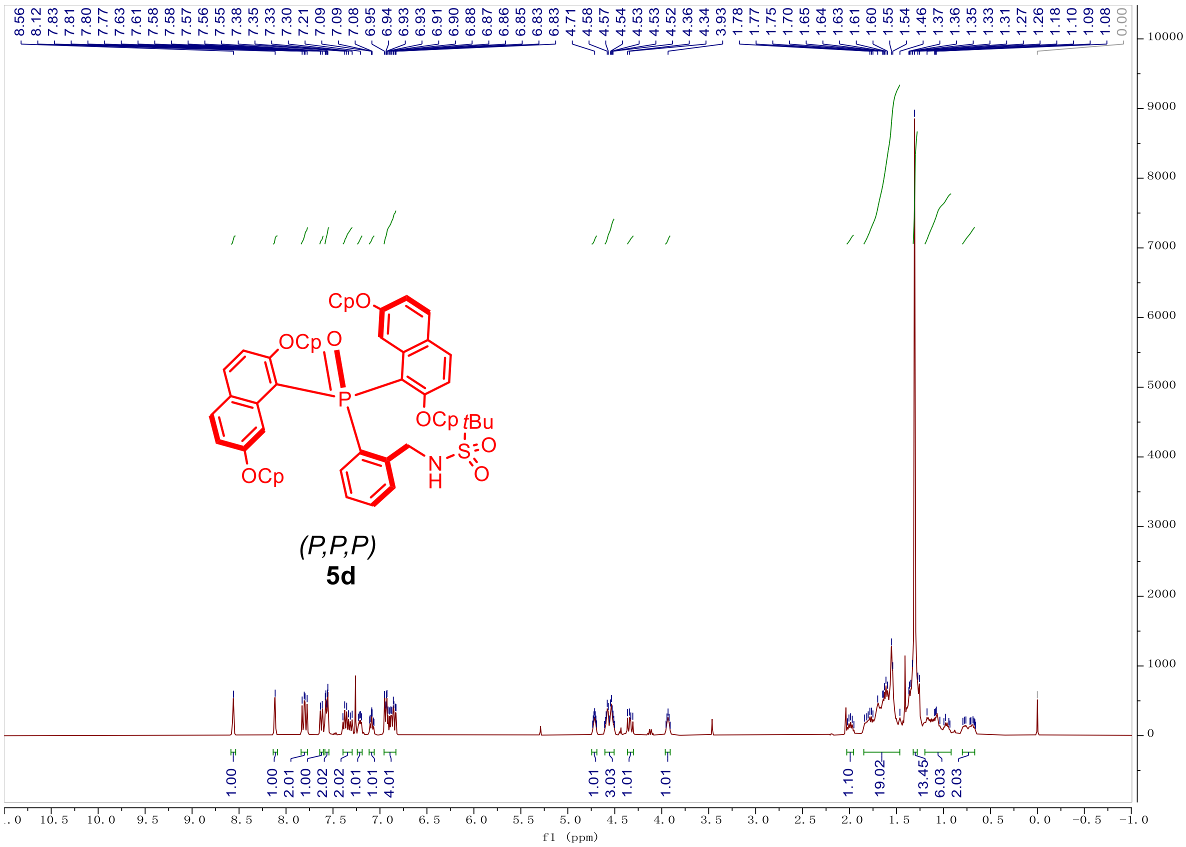
8.56
8.12
7.83
7.81
7.80
7.77
7.63
7.61
7.58
7.58
7.57
7.56
7.55
7.38
7.35
7.33
7.30
7.21
7.09
7.09
7.08
6.95
6.94
6.93
6.93
6.91
6.90
6.88
6.87
6.86
6.85
6.83
6.83
4.71
4.58
4.57
4.54
4.53
4.53
4.52
4.36
4.34
3.93
1.78
1.77
1.75
1.70
1.65
1.64
1.63
1.61
1.60
1.55
1.54
1.46
1.37
1.36
1.35
1.33
1.31
1.27
1.26
1.18
1.10
1.09
1.08
0.00
CpO
OCp
O
P
OCp
tBu
N
H
S
O
O
OCp
(P,P,P)
5d
1.00
1.00
2.01
1.00
2.02
2.02
1.01
1.01
4.01
1.01
3.03
1.01
1.01
1.10
19.02
13.45
6.03
2.03
f1 (ppm)

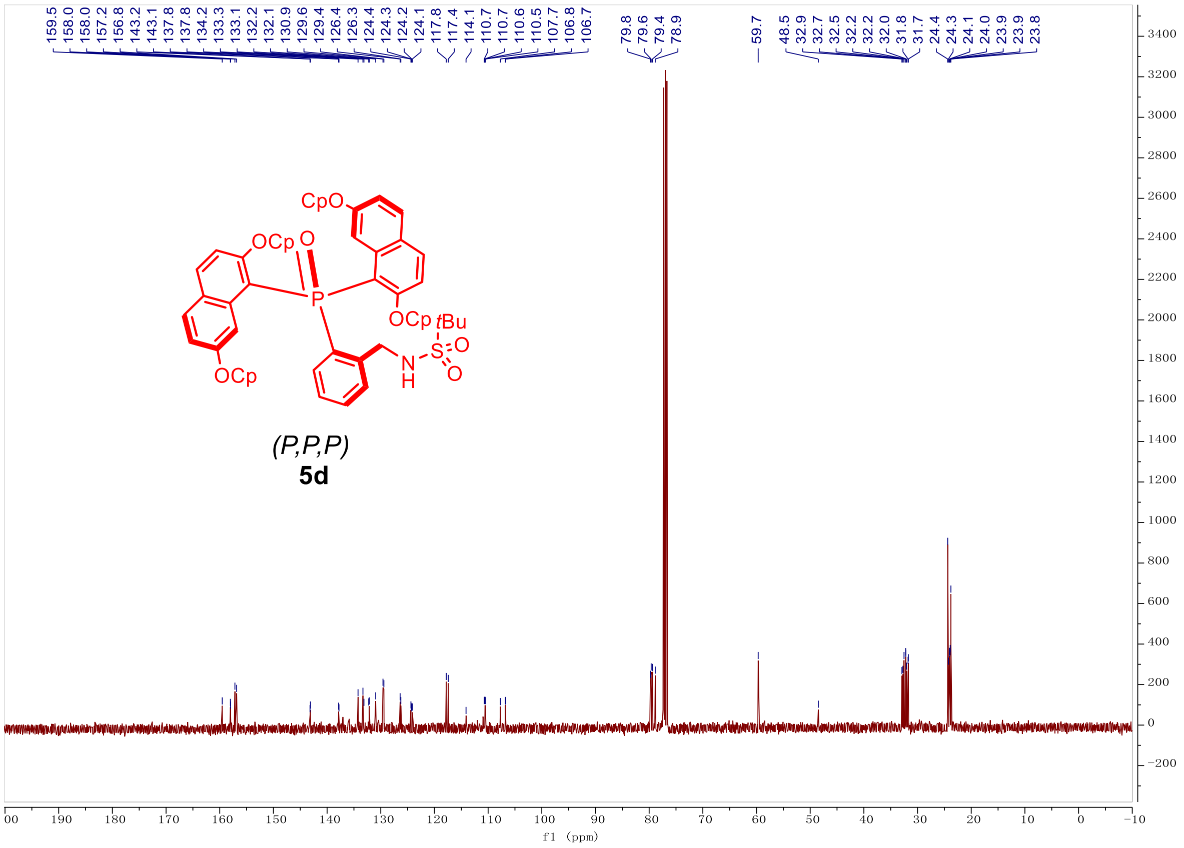
159.5
158.0
158.0
157.2
156.8
143.2
143.1
137.8
137.8
134.2
133.3
133.1
132.2
132.1
130.9
129.6
129.4
126.4
126.3
124.4
124.3
124.2
124.1
117.8
117.4
114.1
110.7
110.7
110.6
110.5
107.7
106.8
106.7
79.8
79.6
79.4
78.9
59.7
48.5
32.9
32.7
32.5
32.2
32.2
32.0
31.8
31.7
24.4
24.3
24.1
24.0
23.9
23.9
23.8
CpO
OCp
O
P
OCp
tBu
N
H
S
O
O
OCp
(P,P,P)
5d
f1 (ppm)

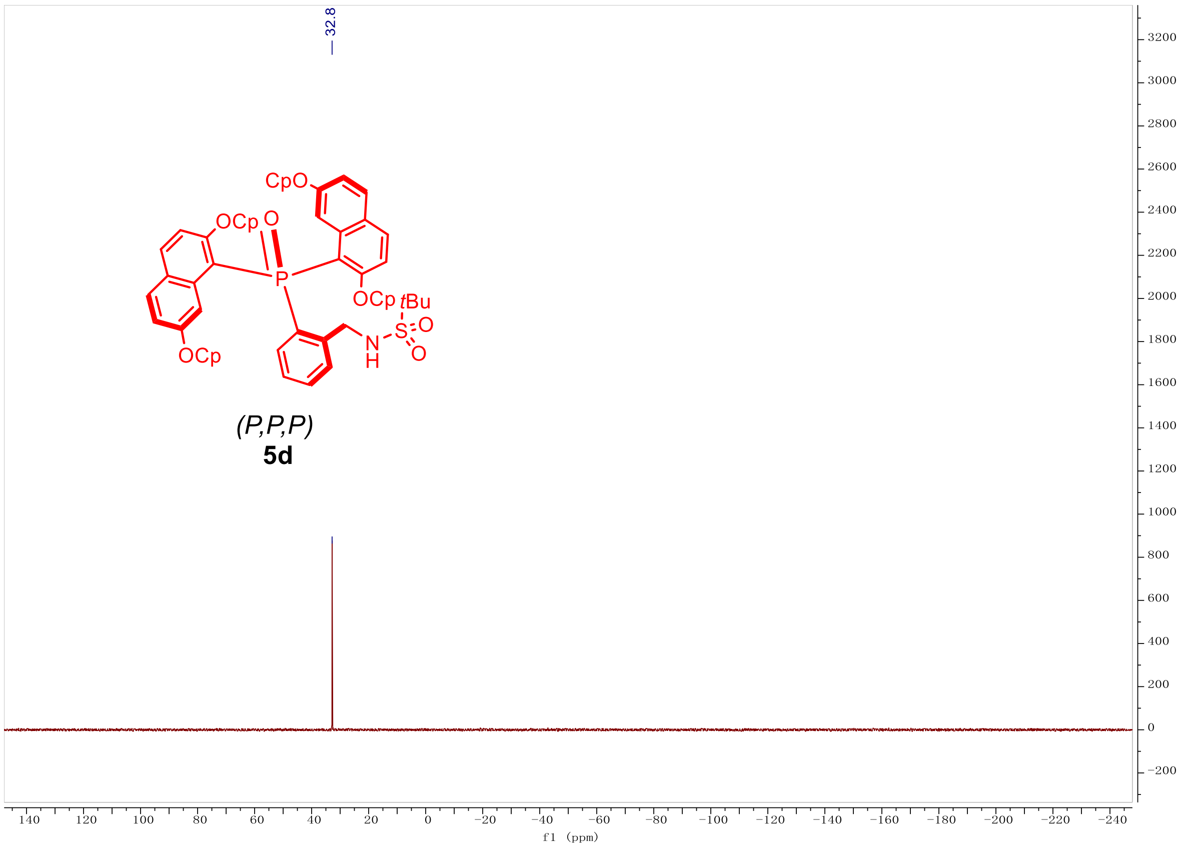

(P,P,P)
5d

2. X-ray Diffraction Data for 4a

Table S1. Crystal data and structure refinement for **4a**.

Identification code	240705b	
Empirical formula	C35.50 H39 Cl N O7 P S	
Formula weight	690.16	
Temperature	293(2) K	
Wavelength	0.71073 Å	
Crystal system	Monoclinic	
Space group	P2(1)	
Unit cell dimensions	a = 10.8152(10) Å	α= 90°.
	b = 18.4090(13) Å	β= 92.353(8)°.
	c = 17.7931(15) Å	γ = 90°.
Volume	3539.6(5) $Å^3$	
Z	4	
Density (calculated)	1.294 Mg/m^3	
Absorption coefficient	0.260 mm^{-1}	
F(000)	1450	
Crystal size	0.20 x 0.18 x 0.10 mm^3	

Theta range for data collection	3.44 to 25.05°.
Index ranges	-9<=h<=12, -21<=k<=21, -21<=l<=21
Reflections collected	17384
Independent reflections	10804 [R(int) = 0.0526]
Completeness to theta = 25.05°	99.5 %
Absorption correction	Semi-empirical from equivalents
Max. and min. transmission	0.9745 and 0.9499
Refinement method	Full-matrix least-squares on F^2
Data / restraints / parameters	10804 / 29 / 860
Goodness-of-fit on F^2	1.019
Final R indices [I>2sigma(I)]	R1 = 0.0765, wR2 = 0.1731
R indices (all data)	R1 = 0.1112, wR2 = 0.1977
Absolute structure parameter	0.03(11)
Largest diff. peak and hole	0.384 and -0.262 e.$Å^{-3}$

Table S2. Atomic coordinates (x 10^4) and equivalent isotropic displacement parameters ($Å^2$x 10^3) for 240705b. U(eq) is defined as one third of the trace of the orthogonalized U^{ij} tensor.

	x	y	z	U(eq)
N(1)	7766(5)	9341(3)	593(3)	56(1)
N(2)	7399(5)	6021(3)	5544(3)	58(1)
O(1)	5838(4)	8040(2)	1477(2)	53(1)
O(2)	4248(5)	6760(3)	-332(3)	64(1)
O(3)	3027(6)	8008(4)	3419(3)	98(2)
O(4)	6507(5)	7436(3)	-741(3)	69(1)
O(5)	6506(7)	5916(3)	3023(3)	89(2)
O(6)	9366(5)	10084(3)	1167(3)	91(2)
O(7)	9640(5)	9609(3)	-87(3)	70(1)
O(8)	9281(4)	7252(2)	6367(2)	52(1)
O(9)	8528(5)	7892(2)	4114(3)	70(1)
O(10)	8518(7)	9318(3)	7935(3)	92(2)
O(11)	10806(5)	8551(2)	4616(3)	62(1)
O(12)	12070(6)	7303(4)	8430(3)	103(2)
O(13)	5966(6)	5291(4)	6213(3)	100(2)
O(14)	5402(5)	5679(3)	4946(3)	73(1)
P(1)	5377(1)	7683(1)	775(1)	45(1)
P(2)	9705(2)	7624(1)	5673(1)	45(1)
S(1)	9222(2)	9505(1)	656(1)	59(1)
S(2)	5968(2)	5840(1)	5664(1)	60(1)
Cl(1)	3469(15)	4284(7)	7861(8)	136(2)
Cl(2)	1552(14)	5102(9)	7143(8)	170(3)
Cl(3)	9429(9)	5835(4)	6707(5)	136(2)
Cl(4)	8312(11)	5686(7)	8053(6)	170(3)
Cl(5)	3832(12)	4484(6)	8117(6)	136(2)
Cl(6)	5975(11)	5196(7)	7828(6)	170(3)
C(1)	7271(6)	8784(3)	61(4)	61(2)
C(2)	5935(6)	8885(3)	-120(4)	53(2)
C(3)	5014(6)	8400(3)	96(4)	46(1)
C(4)	3799(7)	8522(4)	-105(4)	60(2)
C(5)	3481(8)	9158(4)	-521(4)	77(2)
C(6)	4355(8)	9626(4)	-729(4)	74(2)
C(7)	5596(8)	9488(4)	-562(4)	68(2)
C(8)	3957(5)	7166(3)	883(3)	41(1)

C(9)	3616(6)	6696(3)	297(3)	51(2)
C(10)	2608(7)	6210(4)	342(5)	67(2)
C(11)	1991(8)	6204(4)	986(5)	78(2)
C(12)	2248(6)	6682(4)	1591(4)	66(2)
C(13)	3259(6)	7169(3)	1556(3)	47(2)
C(14)	3529(6)	7636(4)	2173(3)	57(2)
C(15)	2815(7)	7620(5)	2792(4)	65(2)
C(16)	1821(9)	7143(6)	2830(5)	89(3)
C(17)	1520(8)	6699(5)	2245(5)	80(2)
C(18)	4267(9)	6189(5)	-861(5)	90(3)
C(19)	4048(11)	8473(7)	3438(6)	124(4)
C(20)	6532(5)	7024(3)	489(3)	42(1)
C(21)	7010(7)	6984(3)	-216(4)	59(2)
C(22)	7985(7)	6493(4)	-364(5)	69(2)
C(23)	8471(7)	6066(4)	195(5)	74(2)
C(24)	8004(7)	6065(4)	906(5)	65(2)
C(25)	7005(6)	6535(3)	1063(4)	51(2)
C(26)	6491(6)	6478(3)	1794(4)	58(2)
C(27)	6970(8)	6000(4)	2325(5)	76(2)
C(28)	7980(8)	5560(4)	2161(6)	81(2)
C(29)	8443(7)	5595(4)	1481(6)	78(2)
C(30)	7030(10)	7469(6)	-1465(4)	106(3)
C(31)	5386(9)	6286(5)	3166(5)	91(3)
C(32)	10081(8)	8728(5)	1054(5)	80(2)
C(33)	9361(11)	8473(8)	1739(6)	150(6)
C(34)	11363(10)	9005(7)	1257(6)	141(5)
C(35)	10184(9)	8157(5)	479(6)	110(3)
C(36)	7766(6)	6519(3)	4944(4)	56(2)
C(37)	9101(6)	6417(3)	4776(3)	47(2)
C(38)	10028(6)	6913(3)	4991(3)	46(1)
C(39)	11232(7)	6775(3)	4839(4)	58(2)
C(40)	11525(8)	6152(4)	4439(4)	70(2)
C(41)	10669(8)	5682(4)	4220(4)	71(2)
C(42)	9449(7)	5810(3)	4353(4)	60(2)
C(43)	8528(6)	8288(3)	5376(4)	48(2)
C(44)	8042(6)	8336(3)	4639(4)	55(2)
C(45)	7065(7)	8830(4)	4462(5)	73(2)
C(46)	6610(8)	9250(4)	5005(6)	78(2)
C(47)	7039(7)	9228(3)	5747(5)	64(2)

C(48)	8055(6)	8756(3)	5935(4)	54(2)
C(49)	8564(7)	8779(3)	6685(4)	64(2)
C(50)	8105(8)	9253(4)	7206(5)	72(2)
C(51)	7081(9)	9704(5)	7005(6)	92(3)
C(52)	6580(8)	9683(4)	6292(6)	82(2)
C(53)	7967(9)	7831(5)	3388(4)	94(3)
C(54)	9613(9)	8918(5)	8141(4)	87(3)
C(55)	11128(5)	8146(3)	5842(3)	42(1)
C(56)	11447(6)	8599(3)	5265(4)	54(2)
C(57)	12506(7)	9080(4)	5386(5)	68(2)
C(58)	13149(8)	9073(4)	6021(5)	76(2)
C(59)	12882(7)	8607(4)	6604(4)	64(2)
C(60)	11857(6)	8129(3)	6530(4)	52(2)
C(61)	11583(6)	7670(4)	7144(4)	60(2)
C(62)	12290(7)	7692(5)	7802(4)	72(2)
C(63)	13338(9)	8138(6)	7857(5)	87(3)
C(64)	13617(8)	8596(5)	7282(5)	88(3)
C(65)	10792(8)	9127(4)	4088(4)	78(2)
C(66)	5176(8)	6622(5)	6042(4)	77(2)
C(67)	4999(9)	7190(6)	5439(5)	104(3)
C(68)	5950(11)	6945(7)	6688(5)	132(4)
C(69)	3929(9)	6344(6)	6300(6)	109(3)
C(70)	11012(10)	6850(7)	8420(5)	112(3)
C(71)	2300(60)	4280(20)	7170(40)	360(30)
C(72)	8380(50)	6230(20)	7272(18)	360(30)
C(73)	5330(30)	4610(30)	8450(20)	360(30)

Table S3. Bond lengths [Å] and angles [°] for 240705b.

N(1)-C(1)	1.481(8)
N(1)-S(1)	1.603(6)
N(1)-H(1)	0.9000
N(2)-C(36)	1.474(8)
N(2)-S(2)	1.606(6)
N(2)-H(2)	0.9000
O(1)-P(1)	1.480(4)
O(2)-C(9)	1.340(7)
O(2)-C(18)	1.413(9)
O(3)-C(15)	1.336(9)
O(3)-C(19)	1.397(12)
O(4)-C(21)	1.350(8)
O(4)-C(30)	1.429(9)
O(5)-C(27)	1.368(10)
O(5)-C(31)	1.423(11)
O(6)-S(1)	1.405(6)
O(7)-S(1)	1.427(5)
O(8)-P(2)	1.500(4)
O(9)-C(44)	1.362(8)
O(9)-C(53)	1.409(8)
O(10)-C(50)	1.358(9)
O(10)-C(54)	1.430(11)
O(11)-C(56)	1.325(8)
O(11)-C(65)	1.416(8)
O(12)-C(62)	1.358(9)
O(12)-C(70)	1.415(12)
O(13)-S(2)	1.407(6)
O(14)-S(2)	1.425(5)
P(1)-C(3)	1.822(6)
P(1)-C(8)	1.824(6)
P(1)-C(20)	1.827(6)
P(2)-C(38)	1.827(6)
P(2)-C(43)	1.828(6)
P(2)-C(55)	1.829(6)
S(1)-C(32)	1.833(8)
S(2)-C(66)	1.818(8)
Cl(1)-C(71)	1.720(10)

Cl(2)-C(71)	1.716(10)
Cl(3)-C(72)	1.707(10)
Cl(4)-C(72)	1.714(10)
Cl(5)-C(73)	1.721(10)
Cl(6)-C(73)	1.719(10)
C(1)-C(2)	1.479(9)
C(1)-H(1C)	0.9700
C(1)-H(1D)	0.9700
C(2)-C(7)	1.400(10)
C(2)-C(3)	1.403(9)
C(3)-C(4)	1.366(9)
C(4)-C(5)	1.419(10)
C(4)-H(4)	0.9300
C(5)-C(6)	1.342(11)
C(5)-H(5)	0.9300
C(6)-C(7)	1.386(11)
C(6)-H(6)	0.9300
C(7)-H(7)	0.9300
C(8)-C(9)	1.392(8)
C(8)-C(13)	1.442(8)
C(9)-C(10)	1.415(9)
C(10)-C(11)	1.348(10)
C(10)-H(10)	0.9300
C(11)-C(12)	1.409(10)
C(11)-H(11)	0.9300
C(12)-C(13)	1.418(9)
C(12)-C(17)	1.433(11)
C(13)-C(14)	1.415(9)
C(14)-C(15)	1.371(9)
C(14)-H(14)	0.9300
C(15)-C(16)	1.393(12)
C(16)-C(17)	1.352(12)
C(16)-H(16)	0.9300
C(17)-H(17)	0.9300
C(18)-H(18A)	0.9600
C(18)-H(18B)	0.9600
C(18)-H(18C)	0.9600
C(19)-H(19A)	0.9600
C(19)-H(19B)	0.9600

C(19)-H(19C)	0.9600
C(20)-C(21)	1.378(9)
C(20)-C(25)	1.440(8)
C(21)-C(22)	1.423(10)
C(22)-C(23)	1.356(10)
C(22)-H(22)	0.9300
C(23)-C(24)	1.382(10)
C(23)-H(23)	0.9300
C(24)-C(29)	1.408(11)
C(24)-C(25)	1.420(9)
C(25)-C(26)	1.440(9)
C(26)-C(27)	1.378(9)
C(26)-H(26)	0.9300
C(27)-C(28)	1.399(11)
C(28)-C(29)	1.329(11)
C(28)-H(28)	0.9300
C(29)-H(29)	0.9300
C(30)-H(30A)	0.9600
C(30)-H(30B)	0.9600
C(30)-H(30C)	0.9600
C(31)-H(31A)	0.9600
C(31)-H(31B)	0.9600
C(31)-H(31C)	0.9600
C(32)-C(35)	1.474(12)
C(32)-C(34)	1.507(14)
C(32)-C(33)	1.545(12)
C(33)-H(33A)	0.9600
C(33)-H(33B)	0.9600
C(33)-H(33C)	0.9600
C(34)-H(34A)	0.9600
C(34)-H(34B)	0.9600
C(34)-H(34C)	0.9600
C(35)-H(35A)	0.9600
C(35)-H(35B)	0.9600
C(35)-H(35C)	0.9600
C(36)-C(37)	1.498(9)
C(36)-H(36A)	0.9700
C(36)-H(36B)	0.9700
C(37)-C(38)	1.398(9)

C(37)-C(42)	1.407(9)
C(38)-C(39)	1.365(9)
C(39)-C(40)	1.393(10)
C(39)-H(39)	0.9300
C(40)-C(41)	1.316(11)
C(40)-H(40)	0.9300
C(41)-C(42)	1.370(10)
C(41)-H(41)	0.9300
C(42)-H(42)	0.9300
C(43)-C(44)	1.396(9)
C(43)-C(48)	1.426(9)
C(44)-C(45)	1.419(10)
C(45)-C(46)	1.346(11)
C(45)-H(45)	0.9300
C(46)-C(47)	1.382(11)
C(46)-H(46)	0.9300
C(47)-C(52)	1.389(11)
C(47)-C(48)	1.430(9)
C(48)-C(49)	1.424(9)
C(49)-C(50)	1.379(10)
C(49)-H(49)	0.9300
C(50)-C(51)	1.418(12)
C(51)-C(52)	1.359(12)
C(51)-H(51)	0.9300
C(52)-H(52)	0.9300
C(53)-H(53A)	0.9600
C(53)-H(53B)	0.9600
C(53)-H(53C)	0.9600
C(54)-H(54A)	0.9600
C(54)-H(54B)	0.9600
C(54)-H(54C)	0.9600
C(55)-C(56)	1.377(8)
C(55)-C(60)	1.430(8)
C(56)-C(57)	1.457(10)
C(57)-C(58)	1.302(10)
C(57)-H(57)	0.9300
C(58)-C(59)	1.387(11)
C(58)-H(58)	0.9300
C(59)-C(60)	1.417(10)

C(59)-C(64)	1.417(11)
C(60)-C(61)	1.422(9)
C(61)-C(62)	1.372(9)
C(61)-H(61)	0.9300
C(62)-C(63)	1.400(12)
C(63)-C(64)	1.368(12)
C(63)-H(63)	0.9300
C(64)-H(64)	0.9300
C(65)-H(65A)	0.9600
C(65)-H(65B)	0.9600
C(65)-H(65C)	0.9600
C(66)-C(67)	1.503(12)
C(66)-C(68)	1.516(12)
C(66)-C(69)	1.531(12)
C(67)-H(67A)	0.9600
C(67)-H(67B)	0.9600
C(67)-H(67C)	0.9600
C(68)-H(68A)	0.9600
C(68)-H(68B)	0.9600
C(68)-H(68C)	0.9600
C(69)-H(69A)	0.9600
C(69)-H(69B)	0.9600
C(69)-H(69C)	0.9600
C(70)-H(70A)	0.9600
C(70)-H(70B)	0.9600
C(70)-H(70C)	0.9600
C(71)-H(71A)	0.9700
C(71)-H(71B)	0.9700
C(72)-H(72A)	0.9700
C(72)-H(72B)	0.9700
C(73)-H(73A)	0.9700
C(73)-H(73B)	0.9700

C(1)-N(1)-S(1)	120.2(4)
C(1)-N(1)-H(1)	106.7
S(1)-N(1)-H(1)	107.1
C(36)-N(2)-S(2)	121.0(4)
C(36)-N(2)-H(2)	106.8
S(2)-N(2)-H(2)	106.6

C(9)-O(2)-C(18)	120.9(6)
C(15)-O(3)-C(19)	117.2(7)
C(21)-O(4)-C(30)	119.2(6)
C(27)-O(5)-C(31)	117.1(6)
C(44)-O(9)-C(53)	120.9(6)
C(50)-O(10)-C(54)	115.8(6)
C(56)-O(11)-C(65)	121.3(5)
C(62)-O(12)-C(70)	118.0(7)
O(1)-P(1)-C(3)	107.1(3)
O(1)-P(1)-C(8)	113.5(3)
C(3)-P(1)-C(8)	106.7(3)
O(1)-P(1)-C(20)	108.6(3)
C(3)-P(1)-C(20)	115.3(3)
C(8)-P(1)-C(20)	105.7(3)
O(8)-P(2)-C(38)	107.1(3)
O(8)-P(2)-C(43)	108.1(3)
C(38)-P(2)-C(43)	115.9(3)
O(8)-P(2)-C(55)	112.8(3)
C(38)-P(2)-C(55)	107.7(3)
C(43)-P(2)-C(55)	105.5(3)
O(6)-S(1)-O(7)	117.8(4)
O(6)-S(1)-N(1)	105.7(3)
O(7)-S(1)-N(1)	108.0(3)
O(6)-S(1)-C(32)	107.5(4)
O(7)-S(1)-C(32)	106.8(4)
N(1)-S(1)-C(32)	111.2(3)
O(13)-S(2)-O(14)	117.4(4)
O(13)-S(2)-N(2)	105.7(3)
O(14)-S(2)-N(2)	107.7(3)
O(13)-S(2)-C(66)	107.4(4)
O(14)-S(2)-C(66)	107.8(4)
N(2)-S(2)-C(66)	110.9(4)
C(2)-C(1)-N(1)	112.1(5)
C(2)-C(1)-H(1C)	109.2
N(1)-C(1)-H(1C)	109.2
C(2)-C(1)-H(1D)	109.2
N(1)-C(1)-H(1D)	109.2
H(1C)-C(1)-H(1D)	107.9
C(7)-C(2)-C(3)	119.1(6)

C(7)-C(2)-C(1)	116.9(6)
C(3)-C(2)-C(1)	124.0(6)
C(4)-C(3)-C(2)	120.6(6)
C(4)-C(3)-P(1)	118.2(5)
C(2)-C(3)-P(1)	120.5(5)
C(3)-C(4)-C(5)	118.8(7)
C(3)-C(4)-H(4)	120.6
C(5)-C(4)-H(4)	120.6
C(6)-C(5)-C(4)	121.0(8)
C(6)-C(5)-H(5)	119.5
C(4)-C(5)-H(5)	119.5
C(5)-C(6)-C(7)	120.7(7)
C(5)-C(6)-H(6)	119.7
C(7)-C(6)-H(6)	119.7
C(6)-C(7)-C(2)	119.6(7)
C(6)-C(7)-H(7)	120.2
C(2)-C(7)-H(7)	120.2
C(9)-C(8)-C(13)	119.6(5)
C(9)-C(8)-P(1)	116.4(4)
C(13)-C(8)-P(1)	123.8(4)
O(2)-C(9)-C(8)	116.2(6)
O(2)-C(9)-C(10)	121.6(6)
C(8)-C(9)-C(10)	122.1(6)
C(11)-C(10)-C(9)	117.5(7)
C(11)-C(10)-H(10)	121.2
C(9)-C(10)-H(10)	121.2
C(10)-C(11)-C(12)	123.7(7)
C(10)-C(11)-H(11)	118.1
C(12)-C(11)-H(11)	118.1
C(11)-C(12)-C(13)	119.3(7)
C(11)-C(12)-C(17)	122.5(7)
C(13)-C(12)-C(17)	118.2(7)
C(14)-C(13)-C(12)	119.1(6)
C(14)-C(13)-C(8)	123.3(5)
C(12)-C(13)-C(8)	117.6(6)
C(15)-C(14)-C(13)	120.4(7)
C(15)-C(14)-H(14)	119.8
C(13)-C(14)-H(14)	119.8
O(3)-C(15)-C(14)	125.2(7)

O(3)-C(15)-C(16)	113.8(7)
C(14)-C(15)-C(16)	120.9(8)
C(17)-C(16)-C(15)	120.4(8)
C(17)-C(16)-H(16)	119.8
C(15)-C(16)-H(16)	119.8
C(16)-C(17)-C(12)	121.0(7)
C(16)-C(17)-H(17)	119.5
C(12)-C(17)-H(17)	119.5
O(2)-C(18)-H(18A)	109.5
O(2)-C(18)-H(18B)	109.5
H(18A)-C(18)-H(18B)	109.5
O(2)-C(18)-H(18C)	109.5
H(18A)-C(18)-H(18C)	109.5
H(18B)-C(18)-H(18C)	109.5
O(3)-C(19)-H(19A)	109.5
O(3)-C(19)-H(19B)	109.5
H(19A)-C(19)-H(19B)	109.5
O(3)-C(19)-H(19C)	109.5
H(19A)-C(19)-H(19C)	109.5
H(19B)-C(19)-H(19C)	109.5
C(21)-C(20)-C(25)	118.4(5)
C(21)-C(20)-P(1)	125.1(5)
C(25)-C(20)-P(1)	116.4(4)
O(4)-C(21)-C(20)	116.2(6)
O(4)-C(21)-C(22)	123.0(6)
C(20)-C(21)-C(22)	120.8(7)
C(23)-C(22)-C(21)	120.0(7)
C(23)-C(22)-H(22)	120.0
C(21)-C(22)-H(22)	120.0
C(22)-C(23)-C(24)	121.8(7)
C(22)-C(23)-H(23)	119.1
C(24)-C(23)-H(23)	119.1
C(23)-C(24)-C(29)	122.8(7)
C(23)-C(24)-C(25)	119.3(7)
C(29)-C(24)-C(25)	117.9(7)
C(24)-C(25)-C(26)	117.3(6)
C(24)-C(25)-C(20)	119.6(6)
C(26)-C(25)-C(20)	123.1(6)
C(27)-C(26)-C(25)	121.2(7)

C(27)-C(26)-H(26)	119.4
C(25)-C(26)-H(26)	119.4
O(5)-C(27)-C(26)	123.6(8)
O(5)-C(27)-C(28)	116.3(7)
C(26)-C(27)-C(28)	120.1(8)
C(29)-C(28)-C(27)	119.2(7)
C(29)-C(28)-H(28)	120.4
C(27)-C(28)-H(28)	120.4
C(28)-C(29)-C(24)	124.2(7)
C(28)-C(29)-H(29)	117.9
C(24)-C(29)-H(29)	117.9
O(4)-C(30)-H(30A)	109.5
O(4)-C(30)-H(30B)	109.5
H(30A)-C(30)-H(30B)	109.5
O(4)-C(30)-H(30C)	109.5
H(30A)-C(30)-H(30C)	109.5
H(30B)-C(30)-H(30C)	109.5
O(5)-C(31)-H(31A)	109.5
O(5)-C(31)-H(31B)	109.5
H(31A)-C(31)-H(31B)	109.5
O(5)-C(31)-H(31C)	109.5
H(31A)-C(31)-H(31C)	109.5
H(31B)-C(31)-H(31C)	109.5
C(35)-C(32)-C(34)	108.2(8)
C(35)-C(32)-C(33)	112.7(9)
C(34)-C(32)-C(33)	113.7(9)
C(35)-C(32)-S(1)	109.9(6)
C(34)-C(32)-S(1)	106.0(7)
C(33)-C(32)-S(1)	106.2(6)
C(32)-C(33)-H(33A)	109.5
C(32)-C(33)-H(33B)	109.5
H(33A)-C(33)-H(33B)	109.5
C(32)-C(33)-H(33C)	109.5
H(33A)-C(33)-H(33C)	109.5
H(33B)-C(33)-H(33C)	109.5
C(32)-C(34)-H(34A)	109.5
C(32)-C(34)-H(34B)	109.5
H(34A)-C(34)-H(34B)	109.5
C(32)-C(34)-H(34C)	109.5

H(34A)-C(34)-H(34C)	109.5
H(34B)-C(34)-H(34C)	109.5
C(32)-C(35)-H(35A)	109.5
C(32)-C(35)-H(35B)	109.5
H(35A)-C(35)-H(35B)	109.5
C(32)-C(35)-H(35C)	109.5
H(35A)-C(35)-H(35C)	109.5
H(35B)-C(35)-H(35C)	109.5
N(2)-C(36)-C(37)	111.0(5)
N(2)-C(36)-H(36A)	109.4
C(37)-C(36)-H(36A)	109.4
N(2)-C(36)-H(36B)	109.4
C(37)-C(36)-H(36B)	109.4
H(36A)-C(36)-H(36B)	108.0
C(38)-C(37)-C(42)	117.4(6)
C(38)-C(37)-C(36)	123.4(6)
C(42)-C(37)-C(36)	119.2(5)
C(39)-C(38)-C(37)	120.3(6)
C(39)-C(38)-P(2)	118.3(5)
C(37)-C(38)-P(2)	119.6(5)
C(38)-C(39)-C(40)	119.5(7)
C(38)-C(39)-H(39)	120.2
C(40)-C(39)-H(39)	120.2
C(41)-C(40)-C(39)	121.4(8)
C(41)-C(40)-H(40)	119.3
C(39)-C(40)-H(40)	119.3
C(40)-C(41)-C(42)	120.4(7)
C(40)-C(41)-H(41)	119.8
C(42)-C(41)-H(41)	119.8
C(41)-C(42)-C(37)	120.7(6)
C(41)-C(42)-H(42)	119.6
C(37)-C(42)-H(42)	119.6
C(44)-C(43)-C(48)	119.0(6)
C(44)-C(43)-P(2)	123.0(5)
C(48)-C(43)-P(2)	117.9(5)
O(9)-C(44)-C(43)	117.7(6)
O(9)-C(44)-C(45)	122.5(7)
C(43)-C(44)-C(45)	119.7(7)
C(46)-C(45)-C(44)	120.1(7)

C(46)-C(45)-H(45)	120.0
C(44)-C(45)-H(45)	120.0
C(45)-C(46)-C(47)	123.3(7)
C(45)-C(46)-H(46)	118.3
C(47)-C(46)-H(46)	118.3
C(46)-C(47)-C(52)	122.2(7)
C(46)-C(47)-C(48)	117.8(7)
C(52)-C(47)-C(48)	119.8(8)
C(49)-C(48)-C(43)	122.5(6)
C(49)-C(48)-C(47)	117.7(6)
C(43)-C(48)-C(47)	119.9(7)
C(50)-C(49)-C(48)	120.9(7)
C(50)-C(49)-H(49)	119.5
C(48)-C(49)-H(49)	119.5
O(10)-C(50)-C(49)	125.7(8)
O(10)-C(50)-C(51)	114.3(8)
C(49)-C(50)-C(51)	119.9(8)
C(52)-C(51)-C(50)	119.8(8)
C(52)-C(51)-H(51)	120.1
C(50)-C(51)-H(51)	120.1
C(51)-C(52)-C(47)	121.9(8)
C(51)-C(52)-H(52)	119.1
C(47)-C(52)-H(52)	119.1
O(9)-C(53)-H(53A)	109.5
O(9)-C(53)-H(53B)	109.5
H(53A)-C(53)-H(53B)	109.5
O(9)-C(53)-H(53C)	109.5
H(53A)-C(53)-H(53C)	109.5
H(53B)-C(53)-H(53C)	109.5
O(10)-C(54)-H(54A)	109.5
O(10)-C(54)-H(54B)	109.5
H(54A)-C(54)-H(54B)	109.5
O(10)-C(54)-H(54C)	109.5
H(54A)-C(54)-H(54C)	109.5
H(54B)-C(54)-H(54C)	109.5
C(56)-C(55)-C(60)	120.4(6)
C(56)-C(55)-P(2)	115.5(5)
C(60)-C(55)-P(2)	124.1(4)
O(11)-C(56)-C(55)	118.1(6)

O(11)-C(56)-C(57)	123.3(6)
C(55)-C(56)-C(57)	118.5(7)
C(58)-C(57)-C(56)	120.7(7)
C(58)-C(57)-H(57)	119.6
C(56)-C(57)-H(57)	119.6
C(57)-C(58)-C(59)	122.4(7)
C(57)-C(58)-H(58)	118.8
C(59)-C(58)-H(58)	118.8
C(58)-C(59)-C(60)	120.0(7)
C(58)-C(59)-C(64)	121.3(8)
C(60)-C(59)-C(64)	118.7(7)
C(59)-C(60)-C(61)	119.0(6)
C(59)-C(60)-C(55)	117.9(6)
C(61)-C(60)-C(55)	123.1(6)
C(62)-C(61)-C(60)	120.7(7)
C(62)-C(61)-H(61)	119.6
C(60)-C(61)-H(61)	119.6
O(12)-C(62)-C(61)	125.2(8)
O(12)-C(62)-C(63)	114.9(7)
C(61)-C(62)-C(63)	119.9(8)
C(64)-C(63)-C(62)	120.8(7)
C(64)-C(63)-H(63)	119.6
C(62)-C(63)-H(63)	119.6
C(63)-C(64)-C(59)	120.7(8)
C(63)-C(64)-H(64)	119.6
C(59)-C(64)-H(64)	119.6
O(11)-C(65)-H(65A)	109.5
O(11)-C(65)-H(65B)	109.5
H(65A)-C(65)-H(65B)	109.5
O(11)-C(65)-H(65C)	109.5
H(65A)-C(65)-H(65C)	109.5
H(65B)-C(65)-H(65C)	109.5
C(67)-C(66)-C(68)	108.6(9)
C(67)-C(66)-C(69)	111.0(8)
C(68)-C(66)-C(69)	111.6(7)
C(67)-C(66)-S(2)	109.6(5)
C(68)-C(66)-S(2)	109.7(6)
C(69)-C(66)-S(2)	106.3(6)
C(66)-C(67)-H(67A)	109.5

C(66)-C(67)-H(67B)	109.5
H(67A)-C(67)-H(67B)	109.5
C(66)-C(67)-H(67C)	109.5
H(67A)-C(67)-H(67C)	109.5
H(67B)-C(67)-H(67C)	109.5
C(66)-C(68)-H(68A)	109.5
C(66)-C(68)-H(68B)	109.5
H(68A)-C(68)-H(68B)	109.5
C(66)-C(68)-H(68C)	109.5
H(68A)-C(68)-H(68C)	109.5
H(68B)-C(68)-H(68C)	109.5
C(66)-C(69)-H(69A)	109.5
C(66)-C(69)-H(69B)	109.5
H(69A)-C(69)-H(69B)	109.5
C(66)-C(69)-H(69C)	109.5
H(69A)-C(69)-H(69C)	109.5
H(69B)-C(69)-H(69C)	109.5
O(12)-C(70)-H(70A)	109.5
O(12)-C(70)-H(70B)	109.5
H(70A)-C(70)-H(70B)	109.5
O(12)-C(70)-H(70C)	109.5
H(70A)-C(70)-H(70C)	109.5
H(70B)-C(70)-H(70C)	109.5
Cl(2)-C(71)-Cl(1)	110.6(11)
Cl(2)-C(71)-H(71A)	109.5
Cl(1)-C(71)-H(71A)	109.5
Cl(2)-C(71)-H(71B)	109.5
Cl(1)-C(71)-H(71B)	109.5
H(71A)-C(71)-H(71B)	108.1
Cl(3)-C(72)-Cl(4)	106.5(9)
Cl(3)-C(72)-H(72A)	110.4
Cl(4)-C(72)-H(72A)	110.4
Cl(3)-C(72)-H(72B)	110.4
Cl(4)-C(72)-H(72B)	110.4
H(72A)-C(72)-H(72B)	108.6
Cl(6)-C(73)-Cl(5)	105.1(10)
Cl(6)-C(73)-H(73A)	110.7
Cl(5)-C(73)-H(73A)	110.7
Cl(6)-C(73)-H(73B)	110.7

Cl(5)-C(73)-H(73B)	110.7
H(73A)-C(73)-H(73B)	108.8

Symmetry transformations used to generate equivalent atoms:

Table S4. Anisotropic displacement parameters ($Å^2 x 10^3$) for 240705b. The anisotropic displacement factor exponent takes the form: $-2\pi^2[h^2 a^{*2}U^{11} + ... + 2 h k a^* b^* U^{12}]$

	U^{11}	U^{22}	U^{33}	U^{23}	U^{13}	U^{12}
N(1)	45(4)	57(3)	68(3)	6(3)	8(3)	12(3)
N(2)	35(3)	64(4)	74(3)	3(3)	-1(3)	12(3)
O(1)	43(3)	40(2)	77(3)	-13(2)	-2(2)	3(2)
O(2)	64(3)	57(3)	72(3)	-14(2)	-1(2)	-10(2)
O(3)	94(5)	124(5)	78(4)	-21(4)	21(3)	0(4)
O(4)	76(4)	63(3)	69(3)	-1(2)	14(2)	5(2)
O(5)	109(5)	80(4)	77(4)	13(3)	-16(3)	9(4)
O(6)	75(4)	96(4)	100(4)	-32(3)	-16(3)	5(3)
O(7)	69(4)	69(3)	72(3)	10(3)	6(2)	-19(3)
O(8)	40(3)	46(2)	70(3)	6(2)	3(2)	4(2)
O(9)	74(4)	50(3)	85(3)	5(3)	-9(3)	10(2)
O(10)	112(5)	74(4)	92(4)	-13(3)	29(4)	10(4)
O(11)	67(3)	52(3)	66(3)	9(2)	4(2)	-16(2)
O(12)	96(5)	131(5)	82(4)	30(4)	-19(3)	-3(4)
O(13)	96(5)	99(4)	108(4)	33(4)	30(4)	11(4)
O(14)	67(4)	78(3)	75(3)	-13(3)	12(2)	-29(3)
P(1)	37(1)	31(1)	66(1)	-3(1)	0(1)	2(1)
P(2)	37(1)	30(1)	67(1)	3(1)	1(1)	1(1)
S(1)	52(1)	53(1)	70(1)	-2(1)	-3(1)	1(1)
S(2)	55(1)	51(1)	77(1)	2(1)	13(1)	2(1)
Cl(1)	169(6)	88(4)	148(5)	6(3)	-10(4)	-15(4)
Cl(2)	173(7)	166(6)	171(5)	25(5)	26(5)	21(5)
Cl(3)	169(6)	88(4)	148(5)	6(3)	-10(4)	-15(4)
Cl(4)	173(7)	166(6)	171(5)	25(5)	26(5)	21(5)
Cl(5)	169(6)	88(4)	148(5)	6(3)	-10(4)	-15(4)
Cl(6)	173(7)	166(6)	171(5)	25(5)	26(5)	21(5)
C(1)	49(5)	34(3)	101(5)	-14(3)	15(4)	-3(3)
C(2)	49(4)	34(3)	77(4)	0(3)	9(3)	0(3)
C(3)	36(4)	28(3)	74(4)	-6(3)	2(3)	2(3)
C(4)	50(5)	47(4)	83(5)	6(3)	-6(3)	16(3)
C(5)	78(6)	62(5)	89(5)	15(4)	-16(4)	25(5)
C(6)	73(6)	44(4)	104(6)	12(4)	8(5)	10(4)
C(7)	63(5)	44(4)	97(5)	-2(4)	-1(4)	-8(4)
C(8)	34(4)	31(3)	58(3)	-4(3)	-2(3)	-1(2)

C(9)	47(4)	42(3)	63(4)	5(3)	-3(3)	6(3)
C(10)	49(5)	59(4)	91(5)	-5(4)	-9(4)	-21(4)
C(11)	71(6)	59(5)	104(6)	12(5)	-8(5)	-19(4)
C(12)	44(4)	59(4)	94(5)	21(4)	1(4)	-8(3)
C(13)	41(4)	37(3)	63(4)	12(3)	-1(3)	-2(3)
C(14)	41(4)	59(4)	71(4)	1(4)	0(3)	1(3)
C(15)	60(5)	73(4)	63(4)	10(4)	10(3)	11(4)
C(16)	73(7)	105(7)	90(6)	28(6)	20(5)	-3(5)
C(17)	58(5)	85(6)	98(6)	23(5)	21(4)	-21(4)
C(18)	93(7)	79(6)	97(6)	-27(5)	3(5)	1(5)
C(19)	126(10)	136(9)	113(7)	-62(7)	32(7)	-23(8)
C(20)	23(3)	31(3)	71(4)	-12(3)	-1(3)	4(2)
C(21)	56(5)	35(3)	87(5)	-2(3)	12(4)	-7(3)
C(22)	53(5)	66(5)	90(5)	-15(4)	16(4)	8(4)
C(23)	58(5)	50(4)	114(7)	-17(4)	0(5)	24(4)
C(24)	52(5)	44(4)	98(6)	-15(4)	-11(4)	3(3)
C(25)	46(4)	20(3)	88(5)	1(3)	-7(3)	-2(3)
C(26)	46(4)	42(3)	83(5)	1(3)	-14(4)	1(3)
C(27)	83(6)	53(4)	89(6)	-2(4)	-38(5)	2(4)
C(28)	82(6)	47(4)	113(7)	9(5)	-26(5)	10(4)
C(29)	57(5)	44(4)	131(7)	-6(5)	-16(5)	15(4)
C(30)	121(9)	115(8)	83(5)	10(6)	26(5)	19(6)
C(31)	92(8)	89(6)	91(6)	27(5)	-5(5)	-26(6)
C(32)	53(5)	98(6)	89(5)	25(5)	2(4)	17(5)
C(33)	134(11)	190(13)	129(8)	83(9)	40(7)	85(10)
C(34)	86(8)	183(12)	150(9)	-29(9)	-57(7)	61(8)
C(35)	62(6)	77(6)	191(10)	10(7)	-2(6)	27(5)
C(36)	37(4)	35(3)	95(5)	7(3)	-6(3)	-10(3)
C(37)	39(4)	36(3)	65(4)	3(3)	-10(3)	-2(3)
C(38)	38(4)	37(3)	62(3)	1(3)	-2(3)	5(3)
C(39)	49(4)	40(3)	84(4)	3(3)	6(3)	0(3)
C(40)	71(6)	50(4)	90(5)	-6(4)	21(4)	7(4)
C(41)	80(6)	40(4)	95(5)	-13(4)	7(4)	5(4)
C(42)	49(5)	35(3)	96(5)	-2(3)	-2(4)	-9(3)
C(43)	34(4)	30(3)	79(4)	-1(3)	-1(3)	1(3)
C(44)	40(4)	41(4)	83(5)	6(3)	-2(3)	-13(3)
C(45)	61(5)	51(4)	105(6)	23(4)	-25(4)	-1(4)
C(46)	67(6)	30(4)	137(7)	-1(4)	-1(5)	15(3)
C(47)	47(5)	31(3)	112(6)	3(4)	0(4)	2(3)

C(48)	36(4)	35(3)	92(5)	4(3)	16(3)	1(3)
C(49)	71(5)	39(3)	84(5)	-1(4)	18(4)	10(3)
C(50)	76(6)	50(4)	93(6)	0(4)	27(5)	4(4)
C(51)	80(7)	67(5)	131(8)	-9(5)	38(6)	18(5)
C(52)	55(5)	48(4)	143(8)	2(5)	6(5)	14(4)
C(53)	99(7)	107(7)	76(5)	4(5)	-23(5)	6(6)
C(54)	100(8)	88(6)	75(5)	-20(5)	13(5)	-15(6)
C(55)	34(4)	35(3)	58(3)	-3(3)	0(3)	0(3)
C(56)	52(4)	35(3)	77(4)	-2(3)	16(4)	-4(3)
C(57)	58(5)	52(4)	94(6)	4(4)	16(4)	-9(4)
C(58)	55(5)	56(4)	116(7)	-16(5)	10(5)	-17(4)
C(59)	60(5)	55(4)	76(4)	-20(4)	2(4)	-5(4)
C(60)	40(4)	47(3)	69(4)	-9(3)	3(3)	-1(3)
C(61)	50(4)	53(4)	76(4)	8(4)	-4(3)	0(3)
C(62)	64(5)	80(5)	72(5)	-1(5)	-12(4)	1(5)
C(63)	76(7)	112(7)	72(5)	-19(5)	-29(5)	9(6)
C(64)	67(6)	103(7)	93(6)	-41(6)	-17(5)	-17(5)
C(65)	87(6)	70(5)	77(5)	26(4)	8(4)	-4(4)
C(66)	62(5)	94(6)	75(5)	-19(5)	9(4)	23(4)
C(67)	77(7)	91(6)	144(8)	12(6)	19(6)	28(5)
C(68)	131(10)	144(10)	121(7)	-81(8)	6(7)	-1(8)
C(69)	80(7)	119(8)	132(8)	-3(7)	39(6)	12(6)
C(70)	100(9)	128(9)	107(7)	40(6)	-5(6)	-13(7)
C(71)	510(70)	400(60)	170(30)	-10(40)	-150(40)	110(60)
C(72)	510(70)	400(60)	170(30)	-10(40)	-150(40)	110(60)
C(73)	510(70)	400(60)	170(30)	-10(40)	-150(40)	110(60)

Table S5. Hydrogen coordinates (x 10^4) and isotropic displacement parameters ($Å^2$x 10^3) for 240705b.

	x	y	z	U(eq)
H(1)	7537	9208	1053	68
H(2)	7704	6207	5981	70
H(1C)	7720	8808	-400	73
H(1D)	7405	8306	279	73
H(4)	3192	8194	28	72
H(5)	2655	9251	-651	93
H(6)	4126	10047	-987	88
H(7)	6200	9794	-742	82
H(10)	2379	5906	-57	80
H(11)	1361	5866	1033	94
H(14)	4194	7955	2158	69
H(16)	1361	7129	3260	107
H(17)	829	6400	2268	96
H(18A)	4761	6329	-1273	134
H(18B)	3438	6087	-1045	134
H(18C)	4615	5762	-625	134
H(19A)	4104	8720	3914	186
H(19B)	3953	8824	3040	186
H(19C)	4788	8196	3374	186
H(22)	8290	6464	-845	83
H(23)	9137	5765	96	89
H(26)	5823	6770	1910	69
H(28)	8324	5248	2523	98
H(29)	9098	5288	1379	93
H(30A)	6575	7812	-1775	159
H(30B)	6987	6998	-1696	159
H(30C)	7879	7620	-1411	159
H(31A)	5154	6188	3671	137
H(31B)	4743	6122	2818	137
H(31C)	5504	6800	3105	137
H(33A)	9320	8862	2096	225
H(33B)	9778	8065	1970	225
H(33C)	8539	8333	1575	225

H(34A)	11316	9378	1632	212
H(34B)	11725	9200	817	212
H(34C)	11866	8612	1452	212
H(35A)	9376	7973	342	165
H(35B)	10693	7769	678	165
H(35C)	10552	8357	43	165
H(36A)	7630	7017	5099	67
H(36B)	7256	6428	4492	67
H(39)	11853	7095	5002	69
H(40)	12344	6067	4324	84
H(41)	10892	5259	3972	86
H(42)	8845	5493	4162	72
H(45)	6739	8863	3972	88
H(46)	5974	9572	4873	93
H(49)	9215	8472	6826	77
H(51)	6754	10012	7359	110
H(52)	5910	9982	6166	98
H(53A)	8431	7497	3096	142
H(53B)	7953	8298	3149	142
H(53C)	7136	7655	3425	142
H(54A)	9827	8999	8663	131
H(54B)	10279	9077	7841	131
H(54C)	9467	8409	8057	131
H(57)	12724	9396	5006	81
H(58)	13811	9393	6085	91
H(61)	10916	7352	7100	72
H(63)	13852	8123	8289	105
H(64)	14296	8905	7338	106
H(65A)	10281	8995	3655	117
H(65B)	11620	9219	3937	117
H(65C)	10466	9556	4313	117
H(67A)	4509	6992	5026	155
H(67B)	5791	7336	5267	155
H(67C)	4583	7603	5640	155
H(68A)	6070	6588	7077	198
H(68B)	5531	7359	6884	198
H(68C)	6739	7092	6512	198
H(69A)	4066	5984	6685	164
H(69B)	3473	6132	5881	164

H(69C)	3467	6741	6497	164
H(70A)	10289	7134	8293	168
H(70B)	10931	6636	8907	168
H(70C)	11097	6474	8053	168
H(71A)	2652	4184	6688	435
H(71B)	1720	3897	7274	435
H(72A)	7574	6256	7013	435
H(72B)	8641	6714	7413	435
H(73A)	5348	4817	8955	435
H(73B)	5776	4151	8472	435

Table S6. Torsion angles [°] for 240705b.

C(1)-N(1)-S(1)-O(6)	176.4(5)
C(1)-N(1)-S(1)-O(7)	49.5(5)
C(1)-N(1)-S(1)-C(32)	-67.3(6)
C(36)-N(2)-S(2)-O(13)	173.1(5)
C(36)-N(2)-S(2)-O(14)	46.9(6)
C(36)-N(2)-S(2)-C(66)	-70.9(6)
S(1)-N(1)-C(1)-C(2)	-159.6(5)
N(1)-C(1)-C(2)-C(7)	70.1(8)
N(1)-C(1)-C(2)-C(3)	-112.7(7)
C(7)-C(2)-C(3)-C(4)	-1.5(9)
C(1)-C(2)-C(3)-C(4)	-178.7(6)
C(7)-C(2)-C(3)-P(1)	-171.3(5)
C(1)-C(2)-C(3)-P(1)	11.5(8)
O(1)-P(1)-C(3)-C(4)	-110.6(5)
C(8)-P(1)-C(3)-C(4)	11.3(6)
C(20)-P(1)-C(3)-C(4)	128.4(5)
O(1)-P(1)-C(3)-C(2)	59.5(5)
C(8)-P(1)-C(3)-C(2)	-178.6(5)
C(20)-P(1)-C(3)-C(2)	-61.5(5)
C(2)-C(3)-C(4)-C(5)	-1.6(10)
P(1)-C(3)-C(4)-C(5)	168.5(5)
C(3)-C(4)-C(5)-C(6)	1.4(11)
C(4)-C(5)-C(6)-C(7)	2.0(12)
C(5)-C(6)-C(7)-C(2)	-5.1(11)
C(3)-C(2)-C(7)-C(6)	4.8(10)
C(1)-C(2)-C(7)-C(6)	-177.8(6)
O(1)-P(1)-C(8)-C(9)	-166.9(4)
C(3)-P(1)-C(8)-C(9)	75.4(5)
C(20)-P(1)-C(8)-C(9)	-47.9(5)
O(1)-P(1)-C(8)-C(13)	7.0(6)
C(3)-P(1)-C(8)-C(13)	-110.7(5)
C(20)-P(1)-C(8)-C(13)	126.0(5)
C(18)-O(2)-C(9)-C(8)	160.2(6)
C(18)-O(2)-C(9)-C(10)	-23.9(9)
C(13)-C(8)-C(9)-O(2)	175.2(5)
P(1)-C(8)-C(9)-O(2)	-10.6(7)
C(13)-C(8)-C(9)-C(10)	-0.7(9)

P(1)-C(8)-C(9)-C(10)	173.5(5)
O(2)-C(9)-C(10)-C(11)	-177.1(7)
C(8)-C(9)-C(10)-C(11)	-1.4(10)
C(9)-C(10)-C(11)-C(12)	4.0(12)
C(10)-C(11)-C(12)-C(13)	-4.3(12)
C(10)-C(11)-C(12)-C(17)	175.4(8)
C(11)-C(12)-C(13)-C(14)	-178.6(6)
C(17)-C(12)-C(13)-C(14)	1.7(10)
C(11)-C(12)-C(13)-C(8)	2.0(9)
C(17)-C(12)-C(13)-C(8)	-177.8(6)
C(9)-C(8)-C(13)-C(14)	-179.0(6)
P(1)-C(8)-C(13)-C(14)	7.2(8)
C(9)-C(8)-C(13)-C(12)	0.4(8)
P(1)-C(8)-C(13)-C(12)	-173.3(5)
C(12)-C(13)-C(14)-C(15)	-0.5(9)
C(8)-C(13)-C(14)-C(15)	178.9(6)
C(19)-O(3)-C(15)-C(14)	1.0(13)
C(19)-O(3)-C(15)-C(16)	176.9(9)
C(13)-C(14)-C(15)-O(3)	176.2(7)
C(13)-C(14)-C(15)-C(16)	0.5(11)
O(3)-C(15)-C(16)-C(17)	-178.0(8)
C(14)-C(15)-C(16)-C(17)	-1.9(13)
C(15)-C(16)-C(17)-C(12)	3.1(13)
C(11)-C(12)-C(17)-C(16)	177.2(8)
C(13)-C(12)-C(17)-C(16)	-3.0(12)
O(1)-P(1)-C(20)-C(21)	-128.4(5)
C(3)-P(1)-C(20)-C(21)	-8.3(6)
C(8)-P(1)-C(20)-C(21)	109.3(5)
O(1)-P(1)-C(20)-C(25)	48.7(5)
C(3)-P(1)-C(20)-C(25)	168.9(4)
C(8)-P(1)-C(20)-C(25)	-73.5(5)
C(30)-O(4)-C(21)-C(20)	174.6(7)
C(30)-O(4)-C(21)-C(22)	-5.0(10)
C(25)-C(20)-C(21)-O(4)	177.7(5)
P(1)-C(20)-C(21)-O(4)	-5.2(8)
C(25)-C(20)-C(21)-C(22)	-2.7(9)
P(1)-C(20)-C(21)-C(22)	174.4(5)
O(4)-C(21)-C(22)-C(23)	178.4(7)
C(20)-C(21)-C(22)-C(23)	-1.2(11)

C(21)-C(22)-C(23)-C(24)	3.3(12)
C(22)-C(23)-C(24)-C(29)	176.0(7)
C(22)-C(23)-C(24)-C(25)	-1.3(11)
C(23)-C(24)-C(25)-C(26)	175.7(6)
C(29)-C(24)-C(25)-C(26)	-1.8(9)
C(23)-C(24)-C(25)-C(20)	-2.6(9)
C(29)-C(24)-C(25)-C(20)	179.9(6)
C(21)-C(20)-C(25)-C(24)	4.6(8)
P(1)-C(20)-C(25)-C(24)	-172.8(5)
C(21)-C(20)-C(25)-C(26)	-173.6(6)
P(1)-C(20)-C(25)-C(26)	9.0(7)
C(24)-C(25)-C(26)-C(27)	1.5(9)
C(20)-C(25)-C(26)-C(27)	179.7(6)
C(31)-O(5)-C(27)-C(26)	7.3(11)
C(31)-O(5)-C(27)-C(28)	-172.1(7)
C(25)-C(26)-C(27)-O(5)	-179.0(6)
C(25)-C(26)-C(27)-C(28)	0.4(10)
O(5)-C(27)-C(28)-C(29)	177.3(7)
C(26)-C(27)-C(28)-C(29)	-2.1(11)
C(27)-C(28)-C(29)-C(24)	1.9(12)
C(23)-C(24)-C(29)-C(28)	-177.2(8)
C(25)-C(24)-C(29)-C(28)	0.2(11)
O(6)-S(1)-C(32)-C(35)	-168.2(6)
O(7)-S(1)-C(32)-C(35)	-40.9(7)
N(1)-S(1)-C(32)-C(35)	76.6(7)
O(6)-S(1)-C(32)-C(34)	-51.5(7)
O(7)-S(1)-C(32)-C(34)	75.7(7)
N(1)-S(1)-C(32)-C(34)	-166.8(6)
O(6)-S(1)-C(32)-C(33)	69.8(8)
O(7)-S(1)-C(32)-C(33)	-163.0(7)
N(1)-S(1)-C(32)-C(33)	-45.5(8)
S(2)-N(2)-C(36)-C(37)	-161.4(5)
N(2)-C(36)-C(37)-C(38)	-106.6(6)
N(2)-C(36)-C(37)-C(42)	76.2(7)
C(42)-C(37)-C(38)-C(39)	-4.8(9)
C(36)-C(37)-C(38)-C(39)	178.0(6)
C(42)-C(37)-C(38)-P(2)	-169.4(5)
C(36)-C(37)-C(38)-P(2)	13.4(8)
O(8)-P(2)-C(38)-C(39)	-108.4(5)

C(43)-P(2)-C(38)-C(39)	130.9(5)
C(55)-P(2)-C(38)-C(39)	13.2(6)
O(8)-P(2)-C(38)-C(37)	56.5(5)
C(43)-P(2)-C(38)-C(37)	-64.1(5)
C(55)-P(2)-C(38)-C(37)	178.1(5)
C(37)-C(38)-C(39)-C(40)	2.8(9)
P(2)-C(38)-C(39)-C(40)	167.7(5)
C(38)-C(39)-C(40)-C(41)	-1.8(11)
C(39)-C(40)-C(41)-C(42)	2.8(12)
C(40)-C(41)-C(42)-C(37)	-4.9(11)
C(38)-C(37)-C(42)-C(41)	5.8(9)
C(36)-C(37)-C(42)-C(41)	-176.9(6)
O(8)-P(2)-C(43)-C(44)	-130.4(5)
C(38)-P(2)-C(43)-C(44)	-10.3(6)
C(55)-P(2)-C(43)-C(44)	108.7(5)
O(8)-P(2)-C(43)-C(48)	46.8(5)
C(38)-P(2)-C(43)-C(48)	166.9(4)
C(55)-P(2)-C(43)-C(48)	-74.1(5)
C(53)-O(9)-C(44)-C(43)	170.5(6)
C(53)-O(9)-C(44)-C(45)	-8.8(10)
C(48)-C(43)-C(44)-O(9)	178.8(5)
P(2)-C(43)-C(44)-O(9)	-4.0(8)
C(48)-C(43)-C(44)-C(45)	-1.8(9)
P(2)-C(43)-C(44)-C(45)	175.3(5)
O(9)-C(44)-C(45)-C(46)	179.3(7)
C(43)-C(44)-C(45)-C(46)	0.0(10)
C(44)-C(45)-C(46)-C(47)	-0.5(12)
C(45)-C(46)-C(47)-C(52)	178.0(7)
C(45)-C(46)-C(47)-C(48)	2.8(11)
C(44)-C(43)-C(48)-C(49)	-175.2(6)
P(2)-C(43)-C(48)-C(49)	7.5(8)
C(44)-C(43)-C(48)-C(47)	4.2(9)
P(2)-C(43)-C(48)-C(47)	-173.1(5)
C(46)-C(47)-C(48)-C(49)	174.7(6)
C(52)-C(47)-C(48)-C(49)	-0.6(10)
C(46)-C(47)-C(48)-C(43)	-4.6(10)
C(52)-C(47)-C(48)-C(43)	-180.0(6)
C(43)-C(48)-C(49)-C(50)	178.2(6)
C(47)-C(48)-C(49)-C(50)	-1.2(10)

C(54)-O(10)-C(50)-C(49)	7.8(11)
C(54)-O(10)-C(50)-C(51)	-174.9(7)
C(48)-C(49)-C(50)-O(10)	179.7(7)
C(48)-C(49)-C(50)-C(51)	2.5(11)
O(10)-C(50)-C(51)-C(52)	-179.5(8)
C(49)-C(50)-C(51)-C(52)	-2.0(12)
C(50)-C(51)-C(52)-C(47)	0.2(13)
C(46)-C(47)-C(52)-C(51)	-174.0(8)
C(48)-C(47)-C(52)-C(51)	1.1(12)
O(8)-P(2)-C(55)-C(56)	-169.1(4)
C(38)-P(2)-C(55)-C(56)	72.9(5)
C(43)-P(2)-C(55)-C(56)	-51.4(5)
O(8)-P(2)-C(55)-C(60)	7.9(6)
C(38)-P(2)-C(55)-C(60)	-110.0(5)
C(43)-P(2)-C(55)-C(60)	125.7(5)
C(65)-O(11)-C(56)-C(55)	159.7(6)
C(65)-O(11)-C(56)-C(57)	-23.3(10)
C(60)-C(55)-C(56)-O(11)	174.4(5)
P(2)-C(55)-C(56)-O(11)	-8.4(7)
C(60)-C(55)-C(56)-C(57)	-2.7(9)
P(2)-C(55)-C(56)-C(57)	174.5(5)
O(11)-C(56)-C(57)-C(58)	-175.8(7)
C(55)-C(56)-C(57)-C(58)	1.2(10)
C(56)-C(57)-C(58)-C(59)	1.1(12)
C(57)-C(58)-C(59)-C(60)	-1.9(11)
C(57)-C(58)-C(59)-C(64)	178.3(8)
C(58)-C(59)-C(60)-C(61)	-178.3(6)
C(64)-C(59)-C(60)-C(61)	1.5(10)
C(58)-C(59)-C(60)-C(55)	0.3(10)
C(64)-C(59)-C(60)-C(55)	-179.9(6)
C(56)-C(55)-C(60)-C(59)	1.9(9)
P(2)-C(55)-C(60)-C(59)	-175.0(5)
C(56)-C(55)-C(60)-C(61)	-179.5(6)
P(2)-C(55)-C(60)-C(61)	3.6(9)
C(59)-C(60)-C(61)-C(62)	0.6(10)
C(55)-C(60)-C(61)-C(62)	-177.9(7)
C(70)-O(12)-C(62)-C(61)	-1.7(13)
C(70)-O(12)-C(62)-C(63)	178.8(8)
C(60)-C(61)-C(62)-O(12)	176.7(7)

C(60)-C(61)-C(62)-C(63)	-3.8(11)
O(12)-C(62)-C(63)-C(64)	-175.4(8)
C(61)-C(62)-C(63)-C(64)	5.0(13)
C(62)-C(63)-C(64)-C(59)	-2.9(14)
C(58)-C(59)-C(64)-C(63)	179.4(8)
C(60)-C(59)-C(64)-C(63)	-0.4(12)
O(13)-S(2)-C(66)-C(67)	-172.4(7)
O(14)-S(2)-C(66)-C(67)	-45.0(7)
N(2)-S(2)-C(66)-C(67)	72.7(7)
O(13)-S(2)-C(66)-C(68)	68.5(8)
O(14)-S(2)-C(66)-C(68)	-164.2(6)
N(2)-S(2)-C(66)-C(68)	-46.5(7)
O(13)-S(2)-C(66)-C(69)	-52.4(7)
O(14)-S(2)-C(66)-C(69)	75.0(6)
N(2)-S(2)-C(66)-C(69)	-167.3(6)

Symmetry transformations used to generate equivalent atoms:

Table S7. Hydrogen bonds for 240705b [Å and °].

D-H...A	d(D-H)	d(H...A)	d(D...A)	<(DHA)
C(72)-H(72A)...S(2)	0.97	3.00	3.86(6)	147.6
C(72)-H(72A)...N(2)	0.97	2.65	3.23(5)	119.1
C(68)-H(68A)...Cl(6)	0.96	2.89	3.805(18)	159.0
C(65)-H(65C)...Cl(3)#1	0.96	2.98	3.453(11)	112.0
C(61)-H(61)...O(8)	0.93	2.16	2.901(8)	135.8
C(54)-H(54A)...O(7)#2	0.96	2.51	3.400(9)	154.6
C(53)-H(53B)...Cl(1)#3	0.96	2.94	3.772(16)	145.3
C(49)-H(49)...O(8)	0.93	2.39	2.977(7)	120.9
C(34)-H(34A)...Cl(4)#1	0.96	2.50	3.344(18)	146.3
C(33)-H(33A)...Cl(2)#3	0.96	2.83	3.75(2)	160.5
C(31)-H(31A)...O(14)	0.96	2.46	3.358(9)	155.9
C(30)-H(30B)...Cl(4)#4	0.96	2.85	3.678(16)	144.7
C(26)-H(26)...O(1)	0.93	2.46	3.008(7)	117.6
C(22)-H(22)...Cl(4)#4	0.93	2.43	3.216(13)	142.3
C(19)-H(19B)...Cl(6)#3	0.96	2.96	3.890(18)	162.3
C(18)-H(18A)...Cl(6)#4	0.96	2.97	3.542(14)	119.7
C(14)-H(14)...O(1)	0.93	2.20	2.929(8)	134.8
N(2)-H(2)...Cl(3)	0.90	2.33	2.974(11)	128.7
N(2)-H(2)...O(8)	0.90	2.64	3.344(7)	135.4
N(1)-H(1)...Cl(5)#3	0.90	2.19	2.941(13)	140.2
N(1)-H(1)...Cl(1)#3	0.90	2.26	3.109(18)	157.2

Symmetry transformations used to generate equivalent atoms:

#1 -x+2,y+1/2,-z+1 #2 x,y,z+1 #3 -x+1,y+1/2,-z+1

#4 x,y,z-1
